# Formononetin ameliorates depression-like behaviors through rebalancing microglia M1/M2 polarization and inhibiting NLRP3 inflammasome: involvement of activating PPARα-mediated autophagy

**DOI:** 10.1186/s10020-025-01217-2

**Published:** 2025-04-24

**Authors:** Shuaijun Peng, Pan Su, Liming Liu, Zibo Li, Yuan Liu, Lei Tian, Ming Bai, Erping Xu, Yucheng Li

**Affiliations:** 1 Collaborative Innovation Center of Research and Development on the Whole Industry Chain of Yu-Yao, Henan Province, Henan University of Chinese Medicine, Zhengzhou, 450046 P.R. China; 2 Academy of Chinese Medical Sciences, Henan University of Chinese Medicine, Zhengzhou, 450046 P.R. China; 3 College of Pharmacy, Henan University of Chinese Medicine, Zhengzhou, 450046 PR China

**Keywords:** Formononetin, Anti-depression, Microglia M1/M2 polarization, NLRP3 inflammasome, PPARα, Autophagy

## Abstract

**Background:**

The dysregulation of neuroinflammation triggered by imbalance of microglia M1/M2 polarization is a key pathogenic factor and closely associated with occurrence of depression. Formononetin (FMN), a natural non-steroidal isoflavonoid, has been confirmed to exhibit remarkable anti-inflammatory efficacy, but the impact of FMN on depression and the underlying antidepressant mechanisms are still not fully understood. This study aimed to investigate whether the antidepressant effect of FMN is involved in modulating microglia polarization, and if so, what are the underlying mechanisms.

**Methods:**

Lipopolysaccharide (LPS)-induced depressive mice were used to study antidepressant mechanisms of FMN. Microglia cell line BV2 stimulated by LPS was employed to investigate pharmacological mechanisms of FMN. Effects of FMN on neuronal damage were detected by H&E, Nissl and Golgi staining. The efficacy of FMN were evaluated by immunostaining and western blots in *vivo* and *vitro*. In addition, molecular docking, luciferase reporter assay, cellular thermal shift assay (CETSA) and drug affinity responsive target stability (DARTS) were used to confirm the direct target of FMN.

**Results:**

Our results showed that FMN significantly reverses depression-like behaviors, alleviates neuroinflammation and neuronal damage, rebalances M1/M2 polarization, inhibits NLRP3 inflammasome and enhances microglial autophagy level in prefrontal cortex of LPS-induced depressive mice. In vitro assays, results unraveled that autophagy inhibitor chloroquine (CQ) blocks effects of FMN on inhibiting NLRP3 inflammasome and rebalancing M1/M2 polarization. Moreover, PPARα is identified as a direct target of FMN and FMN can activate PPARα-mediated autophagy. Furtherly, combination PPARα agonist (WY14643) with FMN had no significant additive effects on inhibiting NLRP3 inflammasome and rebalancing M1/M2 polarization, whereas PPARα antagonist (GW6471) abrogated these pharmacologic effects of FMN in BV2. Importantly, GW6471 exhibited similar pharmacologic effects to abolish antidepressant effect of FMN in LPS-induced depressive mice.

**Conclusion:**

Our study firstly demonstrated that FMN can rebalance microglia M1/M2 polarization and inhibit NLRP3 inflammasome, with the involvement of activating PPARα-mediated autophagy to ameliorate depression-like behaviors, which provides a novel view to elucidate antidepressant mechanisms of FMN and also offers a potential therapeutic target for depression.

**Supplementary Information:**

The online version contains supplementary material available at 10.1186/s10020-025-01217-2.

## Introduction

Depression continues to grow in prevalence, but the underlying mechanisms remain mysterious, making the development of effective depression treatments challenging. Recently, accumulating evidence reveals that neuroinflammation has critical roles in the pathological consequences of stress exposure, and is tightly associated with onset of depression (Cui et al. 2024; Guo et al. 2023; Tayab et al. 2022). Interventions using drug therapy and neuromodulation for depression have been shown to reduce neuroinflammation while relieving symptoms (Cui et al. 2024; Rooney et al. 2020; Wang et al. 2020). Microglia, as the primary resident immune cells, respond to stress-triggered neuroinflammation to exhibit crucial roles in depression (Wang et al. 2022). Studies have revealed inflammatory factors release and microglial activation as the signs of depression (Guo et al. 2023; Nie et al. 2018). The systemic inflammation induced peripheral injection lipopolysaccharide (LPS) and chronic stress could obviously activate microglia and neuroinflammatory response to damage neurons and induce depressive-like behaviors in mice (He et al. 2023; Jia et al. 2021). Typically, activated microglia can be classified as classically activated state (M1 polarization) or alternatively activated state (M2 polarization), which exhibit pro-/anti-inflammatory effects, respectively, and play key roles in maintaining the homeostasis of neuroinflammation (Hu et al. 2015; Orihuela et al. 2016). Numerous studies have reported that the imbalance of microglia polarization in some depression-related brain regions (prefrontal cortex, hippocampus et al.,) to trigger dysregulation of neuroinflammation and induce depression-like behaviors (Duan et al. 2020; Liu et al. 2024; Tao et al. 2021; Zhou et al. 2024). Therefore, above studies indicated that rebalancing microglia M1/M2 polarization to relieve neuroinflammation is an efficient strategy for depression treatment. NLRP3 (Nucleotide-binding domain, leucine-rich repeat, and pyrin domain-containing protein 3) inflammasome is the most representative inflammasome involved in psychological stress-induced inflammation, which has been considered as a key contributor to neuroinflammation and the important causes of exacerbating depressive symptoms (Gao et al. 2023; Xia et al. 2023; Zhao et al. 2024). The NLRP3 inflammasome is highly expressed in microglia, and a lager body of studies have supported that NLRP3 inflammasome acts critical roles in regulating balance of microglia M1/M2 polarization (Li et al. 2024; Zhang et al. 2022b). In addition, inhibition of excessive NLRP3 inflammasome activation to rebalance microglia polarization also has been confirmed as an effective strategy for ameliorating depression-like behaviors (Alcocer-Gomez et al. 2017; Xia et al. 2023; Zhang et al. 2022b). Moreover, increasing studies showed that normal functional microglia autophagy can inhibit NLRP3 inflammasome activity to modulate neuroinflammation, and impairment of microglia autophagy leads to connive excessive NLRP3 inflammasome activation to induce depression-like behaviors (Alcocer-Gomez et al. 2017; Gan et al. 2024; Yu et al. 2021). Hence, these finding suggested that regulation of microglia autophagy-dependent NLRP3 inflammasome inhibition to counteract neuroinflammation may be a novel antidepressant strategy.

The peroxisome proliferator-activated receptor-α (PPARα), as a ligand-activated nuclear receptor transcription factor, plays an important role in activation of microglia autophagy via promoting the expression of autophagy-related target genes (Luo et al. 2020; Xie, Z.S. et al. 2023b). Moreover, some studies have reported that activation of PPARα and enhancement of autophagy significantly suppress NLRP3 inflammasome activity (Marin-Aguilar et al. 2020; Wang et al. 2021). Together, the above studies suggested that PPARα emerges as a promising therapeutic target for depression treatment through its regulation of NLRP3 inflammasome and microglial polarization.

**Fig. 1:**
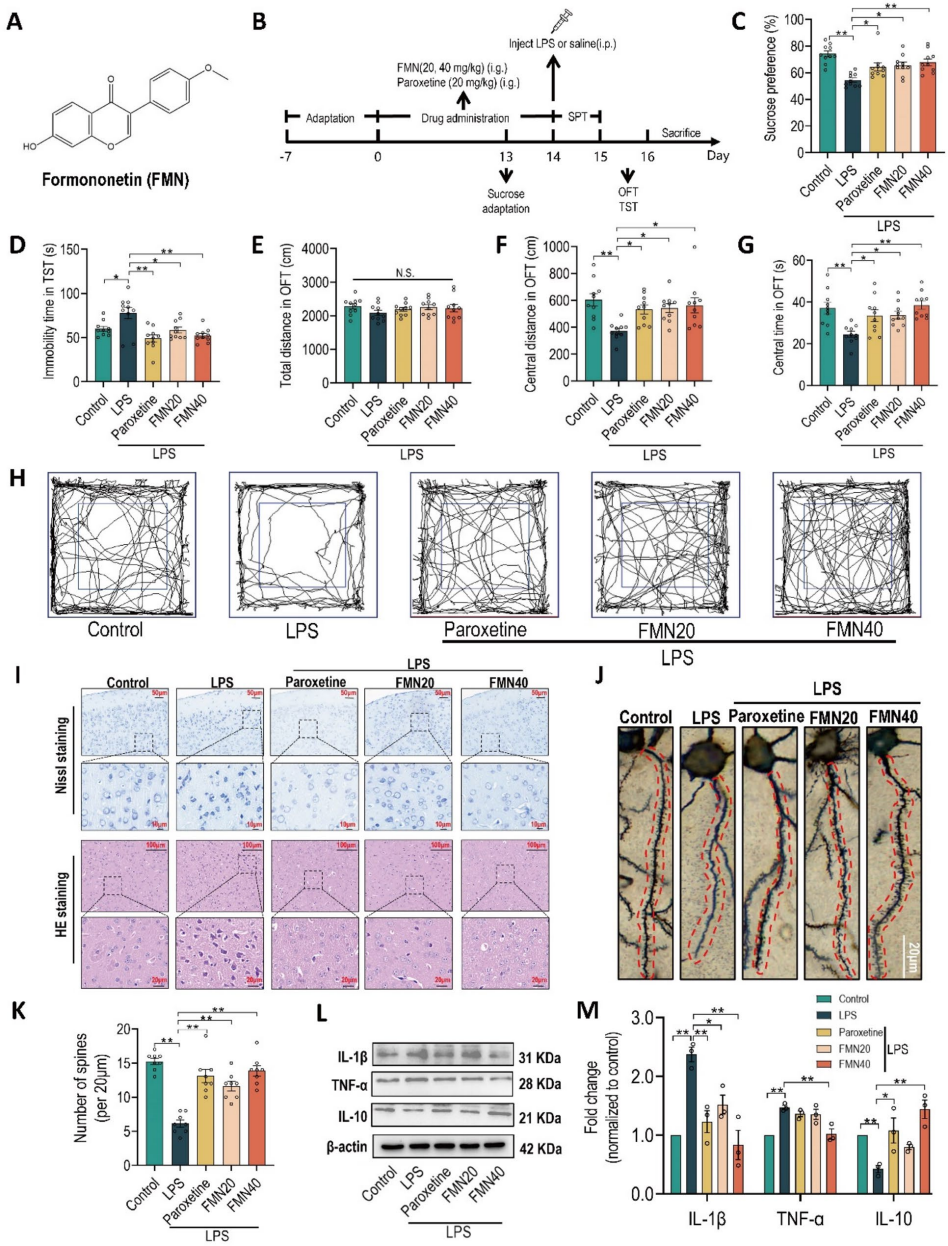
Formononetin ameliorates LPS-induced depression-like behaviors and neuronal injury in mice. **A** Chemical structure of Formononetin (FMN). **B** Schematic diagram of experimental protocols. During the drug administration, paroxetine (20 mg/kg), FMN (20, 40 mg/kg) were given by intragastric (i.g.) administration once a day, the control and LPS groups were gavage with pure water. After the last time drug administration, excepting control group injected with saline, other groups were all intraperitoneally (i.p.) injected with LPS (1 mg/kg). **C** The sucrose preference in SPT. **D** The immobility time in TST. The total distance (**E**), central distance (**F**) and central time (**G**) in central area (blue frame) in OFT. **H** The representative moving track in OFT. **I** Representative images of histological neuronal injury in prefrontal cortex detected by Nissl staining (scale bar, 50 μm) and H&E staining (scale bar, 100 μm), *n* = 3 sections from three mice. **J** Representative images of prefrontal cortical dendritic spine morphology (scale bar, 20 μm). **K** Spine number per 20 μm of dendrites in prefrontal cortex, 8 dendrites from three mice of each group. **L**,** M** Quantification of protein expression levels of TNF-α, IL1-β and IL10 in prefrontal cortex, *n* = 3. The bar graphs were represented as mean ± SEM, and C-G with 10 mice in each group. * *P* < 0.05, ** *P* < 0.01, N.S., not significant

In view of shortcomings of chemical drugs, the unique therapeutic advantage of natural products makes them possible to be an alternative medicament for treating depression (Dai et al. 2022; Xie et al. 2023a). Formononetin (FMN), a natural non-steroidal isoflavonoid (Fig. 1A), is especially rich in Traditional Chinese Medicine *Glycyrrhiza uralensis* (Gancao) and *Astragalus membranaceus* (Huangqi), and has attracted more and more attention because of their anti-inflammatory and neuroprotective efficacy (Singh et al. 2024; Xu et al. 2024). Our previous study for the first time reported that FMN have notable antidepressant effect in corticosterone (CORT)-induced depressive mice, and another study also reported that FMN possesses an ameliorative effect on myocardial infarction-associated depression (Yang et al. 2023; Zhang et al. 2022a). However, the precise antidepressant mechanisms of FMN are still not fully understood. In this study, we investigated whether the antidepressant mechanisms of FMN are involved in rebalancing microglia M1/M2 polarization and inhibiting NLRP3 inflammasome, and further uncovered the underlying molecular mechanisms.

## Materials and methods

### Reagents and chemicals

Formononetin (FMN, purity > 98%, MB1978) was purchased from Dalian Meilun Biotechnology Co., Ltd (Dalian, China). Paroxetine (purity ≥ 98%, P129711) was purchased from Aladdin (Shanghai, China). Lipopolysaccharide (LPS from E, coli 0111: B4, L2630) and Chloroquine diphosphate salt (CQ, C6628) were bought from Sigma-Aldrich (St. Louis, USA). Agonists WY14643 (Pirinixic acid, WY, purity: 99.91%, S8029) and inhibitor GW6471 (GW, purity: 99.48%, S2798) were bought from Selleck Chemicals (Texas, USA). Inhibitor MK-886 (purity: 99.77%, HY-14166) and Minocycline (Mino, HY-17412 A) were purchased from MedChemExpress (MCE, New Jersey, USA). Dimethyl sulfoxide (DMSO, purity > 99.5%, D8371) and BCA Protein Assay Kit (PC0020) were purchased from Beijing Solarbio Science & Technology Co., Ltd (Beijing, China). The Cell Counting Kit-8 (CCK-8, BMU106) was obtained from Abbkine Scientific Co., Ltd (Wuhan, China). The Firefly Luciferase Reporter Gene Assay Kit (RG005) was obtained from Beyotime Biotechnology (Shanghai China). The Endo-Free Plasmid Midi Kit (CW2150S) was obtained from Cwbio (Jiangsu, China).

### Antibodies

The antibodies used for western blot and immunofluorescence were CD68 (1:1000 for WB and 1:200 for IF, ABclonal, A15037), CD206 (1:1000 for WB and 1:500 for IF, Proteintech, 18704-1-AP), NLRP3 (1:1000 for WB and 1:500 for IF, Proteintech, 19771-1-AP), LC3B (1:1000 for WB and 1:500 for IF, Proteintech, 14600-1-AP), SQSTM1 (1:4000 for WB, Sigma-Aldrich, P0067), IL-1β (1:1000 for WB, Servicebio, GB11113), TNF-α (1:1000 for WB, Servicebio, GB113968), IL-10 (1:1000 for WB, Servicebio, GB11108), Iba-1 (1:200 for IF, Servicebio, GB12105) and PPARα (1:1000 for WB and 1:200 for IF, ABclonal, A18252). β-actin (1:50000 for WB, ABclonal, AC026) and GAPDH (1:30000 for WB, Aksomics, KC-5G5).

### Animals and drug administration

The male ICR mice (18–22 g, SPF grade) were obtained from Beijing Vital River Laboratory Animal Technology Co., Ltd and fed in a stable environment (temperature 23–25 ℃, humidity 50–60%) with free access to diet and water and maintained on a constant 12 h light / dark cycle. After one week of adaptation, the experimental mice were randomly divided into five groups (*n* = 10): Control, LPS (1 mg/kg), LPS + Paroxetine (20 mg/kg), LPS + FMN (20 mg/kg) and LPS + FMN (40 mg/kg). The doses of FMN were chosen according to our previous study (Zhang et al. 2022a). To further investigate the roles of PPARα in pharmacologic effects of FMN in *vivo*, the second experimental mice were divided into five groups (*n* = 8): Control, LPS (1 mg/kg), LPS + WY14643 (10 mg/kg), LPS + FMN (40 mg/kg) and LPS + FMN (40 mg/kg) + GW6471 (2 mg/kg). WY14643 (PPARα agonist) as the positive control and GW6471 as the PPARα antagonist to investigate its inhibitory effect on pharmacologic efficacies of FMN.

FMN and paroxetine were dissolved in normal saline and were administered by oral gavage for 14 days, WY14643 or GW6471 was dissolved in 0.5% DMSO, subsequently diluted in normal saline with indicated concentrations, then was injected intraperitoneally after 30 min of FMN administration for 14 consecutive days. LPS was dissolved in sterile normal saline (0.9% w/v NaCl) and performed a single intraperitoneal injection approximately after 1 h of last drug administration on the 14th day.

### Cell culture and treatment

BV2 cells were purchased from Bena Culture Collection (BNCC337749, Beijing, China), HEK-293T cells were purchased from Procell Life Science & Technology Co., Ltd (CL-0005, Wuhan, China). Both of cells were cultured in high-glucose Dulbecco’s modified Eagle’s medium (DMEM, Cytiva, Logan, USA) supplemented with 10% fetal bovine serum (FBS, BI, Kibbutz, IL) and 1% penicillin (100 mg/mL)-streptomycin (100 mg/mL) and incubated at 37 ℃ in a humidified incubator with 5% CO_2_. The BV2 were pre-incubated with various concentrations of FMN (5, 10, 20, 30 µM), Mino (20 µM), CQ (20 µM), WY14643 (20 µM), GW6471 (5 µM) alone or in combination for 1 h respectively, then incubated with or without LPS (1 µg/mL) for 24 h. Above drugs all were dissolved in 0.5% DMSO to form a stock solution, then further diluted to experimental concentrations by using DMEM.

### Behaviors tests

#### Sucrose preference test (SPT)

Before the test, mice were individual housed and provided with two bottles—one containing pure water and the other 1% sucrose solution for 24 h. The positions of the two bottles were switched every 12 h to prevent positional bias After adaptation, the mice were water-deprived for 12 h before intraperitoneal LPS injection (1 mg/kg). Subsequently, each mouse was randomly given access to pure water and 1% sucrose solution for 24 h, with bottle positions alternated every 12 h. Consumption of both liquids was measured and sucrose preference was calculated as: Sucrose preference (%) = sucrose consumption / (sucrose consumption + water consumption) × 100%.

#### Open field test (OFT)

Mice were placed in the center of an open field arena (100 cm ×100 cm × 50 cm) and allowed to explore freely for 6 min. Following a 1-min habituation period, the movement trajectory of each mouse was recorded by SMART 3.0 behavioral analysis system during the next 5 min, and then cleaned the area with 75% ethanol to remove residual odors after each test.

#### Tail suspension test (TST)

The TST was conducted following the OFT. Briefly, Mice were suspended by tape approximately 1 cm from the tip of the tail, and their behavior was video-recorded for 4 min after a 2-min acclimation period. The immobility durations over the 4 min were recorded by SMART 3.0 behavioral analysis system.

### H&E and nissl staining

Freshly dissected brain tissues were immediately fixed in tissue fixative for more than 24 h, then paraffin-embedded and sectioned at 4 μm for subsequent analyses. H&E staining: the tissue sections were deparaffinized with xylene, rehydrated with gradient ethanol, then the sections were incubated with hematoxylin solution for 5 min, washed with distilled water, next immersed in eosin dye for 15 s. After dyeing, the sections from three mice were dehydrated with absolute ethanol, normal butanol and xylene, sealed with neutral gum. Finally, sections of PFC were imaged under an optical microscope (Nikon, Tokyo, Japan).

Nissl staining: the tissue sections were also deparaffinized with xylene, rehydrated with gradient ethanol. After fixing in fixed solution for 15 min, the sections were washed with distilled water and incubated with dye solution for 5 min, then dehydrated, transparentized and sealed. Next, the Nissl-positive cell within PFC were observed by an optical microscope and photographed.

### Immunofluorescent staining

For tissue sections immunofluorescence staining: sections underwent antigen retrieval in citric acid buffer, followed by 30-min blocking with 3% BSA. Afterwards, the sections were incubated with the mixed reagents of primary antibodies against Iba-1, CD68, CD206, NLRP3, LC3B and PPARα overnight at 4 ℃. The next day, the sections were washed three times with PBS (pH 7.4) and 5 min each time. After three 5-min PBS washes (pH 7.4), sections were incubated with Cy3- or FITC-conjugated secondary antibodies for 1 h at room temperature. Finally, ProLong Gold Antifade Reagent with DAPI (Cell Signaling Technology, MA, USA) was dripped into the sections and incubated at room temperature for 10 min in the dark. The fluorescence signals were recorded by a fluorescence microscope (Nikon, Tokyo, Japan) and the number of positive cells Iba-1^+^, Iba-1^+^-CD68, Iba-1^+^-CD206, Iba-1^+^-NLRP3, Iba-1^+^-LC3B and Iba-1^+^-PPARα within three PFC sections of three mice of each group were counted by Image-Pro Plus 6 (Media Cybernetics, Silver Spring, MD, USA).

For BV2 cells immunofluorescence staining: BV2 cells were fixed with 4% paraformaldehyde, permeabilized with 0.1% Triton X-100 and blocked with 5% goat serum for 30 min, after overnight incubation at 4 °C with primary antibodies (CD68, CD206, NLRP3, LC3B, PPARα), cells were treated with Cy3- or FITC-conjugated secondary antibodies for 1 h at room temperature. Finally, the ProLong Gold Antifade Reagent with DAPI was added to prevent fluorescence signal quenching. The signals were recorded by fluorescence microscope and the fluorescence intensity from three cell individual repeated assays were calculated by Image-Pro Plus 6.

### Western blot analysis

The PFC brain tissues or BV2 cells were homogenized in RIPA Lysis Buffer (Cwbio, CW2333S, Beijing, China) containing 0.5% Protease Inhibitor Cocktail Set III, EDTA-Free (Sigma-Aldrich, 539134, St. Louis, USA). After centrifugation (12,000 g, 20 min, 4 °C), supernatants were collected, protein concentration was determined using a BCA assay, followed by SDS-PAGE separation and transfer to PVDF membranes (Millipore, Billerica, USA), incubated with primary antibodies against CD68, CD206, NLRP3, LC3B, SQSTM1, IL-1β, TNF-α, IL-10, PPARα, β-actin and GAPDH overnight at 4 ℃. Subsequently, washed the membrane with PBS containing 0.1% Tween-20 (PBST) three times, 5 min each time, then incubated with HRP-conjugated secondary antibodies for 1 h at room temperature. All animal data have three independent repetitions with random mice from each group. All cell data also have independent repetitions with three batches cell. The membranes were scanned by an imaging system (Bio-Rad, Hercules, USA) and Image-Pro Plus 6 was used to analyze the optical density of the bands.

### Golgi staining

The brain tissues were immersed in the Golgi stain fixing solution (Servicebio, G1069-1, Wuhan, China) for 14 days at room temperature and with solution changes every 2 days, then discarded dying solution and replaced it with the tissue treatment solution for 3 days at 4 ℃. Subsequently, the brain tissues were cut into 60 μm slices. The slices were washed with ultra-pure water, incubated with Golgi developer solution for 30 min. Eight dendrites were randomly selected from three mice of each group for observation, and the dendritic spines within PFC region were observed using the Case Viewer system (3DHISTECH, Budapest, Hungary), and the spine number was were analyzed using Image-Pro Plus 6.

### Cell viability assay

BV2 cells were seeded in 96-well plates (1 × 10^4^ cells/well) and incubated for 12 h, then FMN (5, 10, 20, 30, 40, 50 µM) pre-incubated for 1 h, and incubated with or without LPS (1 µg/mL) for 24 h. Then the culture medium was replaced with CCK solution and incubated for 1 h at 37 ℃. Cell viability was calculated from the optical density at 450 nm using multimode microplate reader (Agilent, Santa Clara, USA).

### Luciferase reporter gene assay

HEK-293T cells were seeded in 24-well plates (1 × 10⁵ cells/well) for 12 h. Before transfection, medium was replaced with fresh complete medium, then co-transfected the PPAR promoter reporter vector J3-TKLuc (500 ng) and pSG5-PPARα (500 ng) with Neofect DNA transfection reagent (Neofect, Beijing, China) for 24 h. After incubated with WY14643 (1 µM), FMN (10, 20, 30 µM) and MK886 (30 µM) for 16 h, collected cells and detected luciferase activity using Firefly Luciferase Reporter Gene Assay Kit. The plasmid vectors used for transfection were gifted from professor Xie at Henan University of Chinese Medicine (Xie, Z.S. et al. 2023b).

### Molecular docking

The 3D structure of formononetin (FMN, CID: 5280378), WY14643 (WY, CID: 5694) was obtained from Pubchem (https://pubchem.ncbi.nlm.nih.gov/). The 3D structure of PPARα (PDB number: LI7G) and other candidates were retrieved from the protein database (https://www.rcsb.org/), then the water molecules and the original ligands were removed through PyMOL. Subsequently, molecular docking was performed with AutoDock Vina using FMN, WY14643 and target proteins, with results visualized in PyMOL. The theoretical binding energy between FMN or WY14643 and PPARα and other candidates was calculated via AutoDock Vina.

### Cellular thermal shift assay (CETSA)

BV2 cells were cultured in 10 cm dishes for two days, cellular proteins were extracted and normalized to 3 mg/mL. For the temperature-dependent thermal shift assay: proteins solution was incubated with 100 µM of FMN or DMSO for 1.5 h at room temperature, then heated the mixed solution at each temperature point from 45 to 60 °C for 5 min, centrifuged the mixed solution at 13,000 g for 15 min at 4 °C to separate the supernatant and precipitate. Afterwards analyzed PPARα using western blot. For the concentration-dependent thermal shift assay: proteins solution was incubated with different concentrations of FMN (25, 50, 100, 200 µM) for 1.5 h at room temperature, then heated the mixed solution at 55 °C for 5 min, the analysis of PPARα as described above and the GAPDH as the internal reference.

### Drug affinity responsive target stability (DARTS)

BV2 cells were cultured in 10 cm dishes for 48 h, and protein lysates were extracted and quantified to 1 mg/mL. The proteins solution was incubated with various concentrations of FMN (0, 25, 50, 100, 200, 400 µM) for 1 h at room temperature. Subsequently, the samples were incubated with TNC buffer (50 mM Tris-HCl, pH 8.0, 50 mM NaCl, 10 mM CaCl_2_) or pronase (0.01 mg/mL, 1:500 dilution) for 15 min at room temperature. Reactions were stopped by adding loading buffer and boiling. The PPARα expression was detected by western blot.

### Statistical analysis

Statistical analysis was carried out by SPSS 20.0 (Armonk, NY, USA). All data were represented as the mean ± SEM and the Figures were drawn by GraphPad Prism 8 (San Diego, USA). One-way analysis of variance (ANOVA) followed by Tukey’s post-hoc test was employed for multiple comparisons. And the CETSA and DARTS were carried out the student’s unpaired t test. Statistical significance was defined for *P* values < 0.05.

## Results

### Formononetin ameliorates LPS-induced depression-like behaviors and neuronal injury in mice

Peripheral LPS challenge via elicit liable neuroinflammation is widely employed to induce depression-like behaviors (Ali et al. 2020). Herein, SPT, OFT, and TST were used to assess the antidepressive effect of formononetin (FMN) in LPS-induced depressive model mice. As shown in Figs. 1B-C, the sucrose preference was significantly recovered by treatment with FMN (20, 40 mg/kg) and paroxetine (20 mg/kg) after LPS challenge. Moreover, TST revealed that treatment with FMN and paroxetine reduced immobility time after LPS challenge (Fig. 1D). In addition, during OFT, there were no significant differences among the groups regarding locomotor activities (total distance) (Fig. 1E). A substantial decrease in the central distance and central time was observed in the LPS-treated group, and the decrease was significantly prevented by treating with FMN and paroxetine (Figs. 1F-H). Collectively, these data indicated that FMN significantly ameliorates LPS-induced depression-like behaviors in mice.

Depressive disorders are most consistently associated with impairment in the prefrontal cortex (PFC), the brain region responsible for emotion regulation (Pizzagalli and Roberts 2022). Therefore, we following focused on the PFC to assess the effect of FMN on the neuroinflammatory injury induced by LPS. As shown in Fig. 1I, the Nissl and H&E staining both exhibited that neurological injury induced by LPS, which can be significantly alleviated by FMN treatment, showing that more cells with intact cellular membrane, clear nucleus and relieved swelling. Dendritic spine loss in PFC correlates with neuronal function, which is also tightly associated with depression (Castren 2013). Our results exerted that the number of dendritic spines of PFC was reduced in LPS group, which was significantly relieved by FMN treatment (Figs. 1J, K). In addition, FMN remarkably decreased the expression of pro-inflammatory cytokines (TNF-α, IL1-β) induced by LPS, and also prominently increased the expression of anti-inflammatory cytokine (IL10) (Figs. 1L, M). Together, our data demonstrated that FMN possesses the ability of inhibiting neuroinflammation to alleviate neuronal injury.

### Formononetin rebalances microglial M1/M2 polarization, inhibits NLRP3 inflammasome and enhances autophagy in prefrontal cortex

**Fig. 2:**
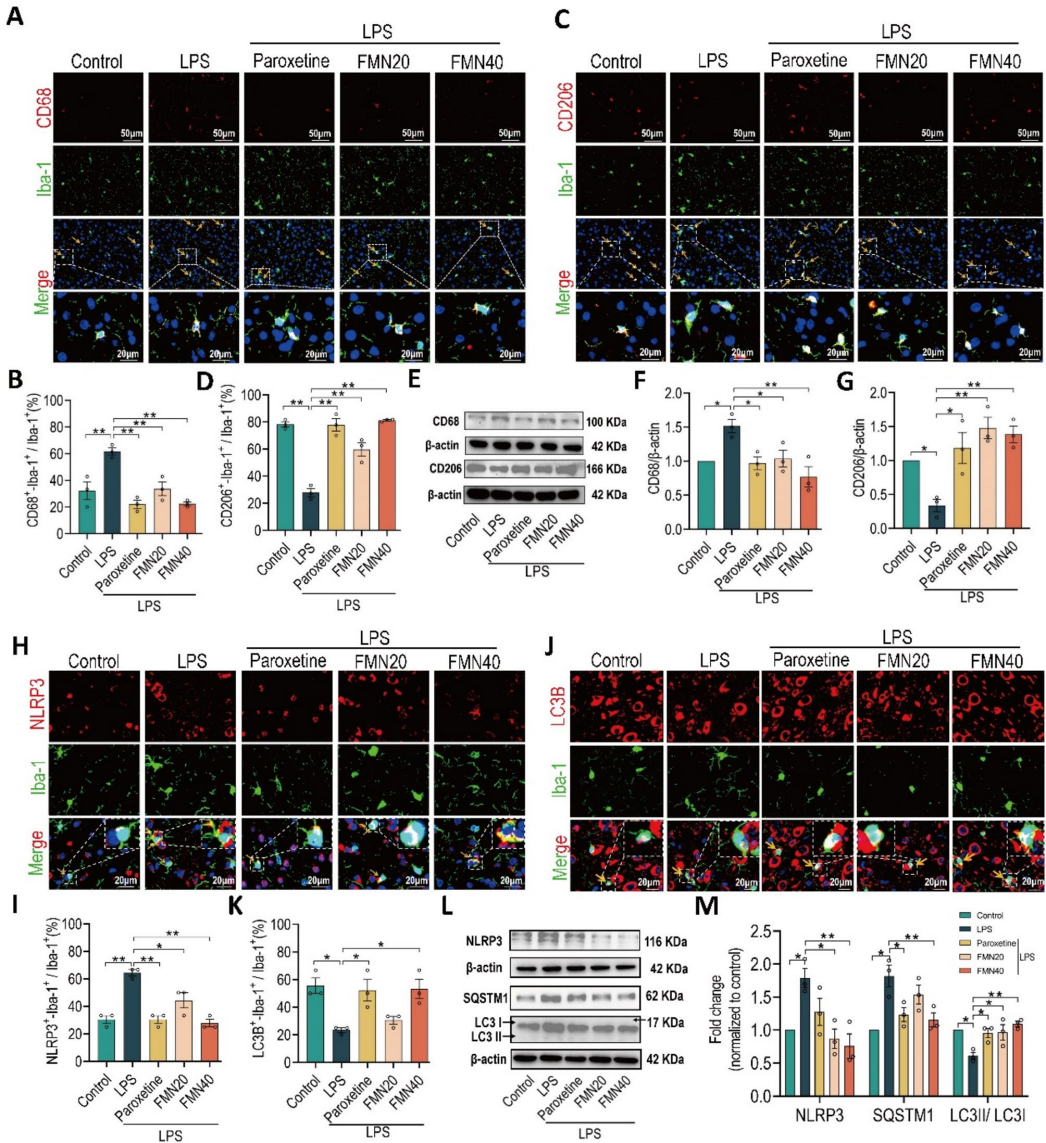
Formononetin rebalances microglia M1/M2 polarization, inhibits NLRP3 inflammasome and enhances autophagy in the PFC. **A**,** C** Representative immunofluorescence images co-staining with Iba-1(Green) and CD68 (Red, M1 marker) or CD206 (Red, M2 marker), scale bar: 50 μm, the bottom images (scale bar: 20 μm) were magnified from the corresponding white dotted frame regions, the yellow arrows represent the M1 or M2 polarization microglia. **B**,** D** Quantification of the percentage of Iba-1^+^/CD68^+^ cells or Iba-1^+^/CD206^+^ cells among Iba-1^+^ cells, *n* = 3. **E**-**G** Quantification of protein expression levels of CD68 and CD206, *n* = 3. **H**,** J** Representative immunofluorescence images co-staining with Iba-1(Green) and NLRP3 (Red) or LC3B (Red, autophagic marker), scale bar: 20 μm, the inner images were magnified from the corresponding white dotted frame regions, the yellow arrows represent the microglia co-expressing with NLRP3 or LC3B. **I**,** K** Quantification of the percentage of Iba-1^+^/NLRP3^+^ cells or Iba-1^+^/LC3B^+^ cells among Iba-1^+^ cells, *n* = 3. **L**,** M** Quantification of protein expression levels of the NLRP3 and SQSTM1, LC3II/I, *n* = 3. The bar graphs were represented as mean ± SEM. * *P* < 0.05, ** *P* < 0.01

Consideration of the imbalance of microglia M1/M2 polarization is the key contributor to develop neuroinflammation, we used double immunofluorescence staining with pan microglial marker (Iba-1) and M1 polarization marker (CD68) or M2 polarization marker (CD206) to determine the effect of FMN on microglia M1/M2 polarization in the PFC. As shown in Figs. 2A-D, LPS challenge significantly increased the percentage of Iba-1^+^/CD68^+^ cells (M1 polarization) among Iba-1^+^ cells, whereas decreased the percentage of Iba-1^+^/CD206^+^ cells (M2 polarization) among Iba-1^+^ cells. However, FMN remarkably decreased the percentage of M1 polarization (Figs. 2A, B) and increased the percentage of M2 polarization (Figs. 2C, D) to counter LPS challenge. In addition, FMN also significantly decreased the protein expression of CD68 while increased CD206 (Figs. 2E-G). These results indicated that FMN can rebalance microglia polarization via inhibiting M1 polarization and promoting M2 polarization in the PFC of LPS-induced depressive mice.

The NLRP3 inflammasome acts the critical roles in regulation of microglia M1/M2 polarization, so we also used double immunofluorescence staining with Iba-1 and NLRP3 to determine the effect of FMN on microglia NLRP3 inflammasome in the PFC. Our results showed that the percentage of Iba-1^+^/NLRP3^+^ cells among Iba-1^+^ cells were significantly increased in LPS group, which was remarkably inhibited by FMN (Figs. 2H, I). As NLRP3 inflammasome inhibition is closed to the microglia autophagy, we furtherly used the double immunofluorescence staining with Iba-1 and LC3B (an autophagic marker) to determine effect of FMN on microglia autophagy level in the PFC. As shown in Figs. 2J, K, the decrease of percentage of Iba-1^+^/LC3B^+^ cells among Iba-1^+^ cells induced by LPS were significantly reversed by FMN. In addition, FMN also prominently decreased the protein expression of NLRP3, SQSTM1 while increased the ratio of LC3II/I in the PFC (Figs. 2L, M). Collectively, these findings indicated that FMN possessed the ability of inhibiting NLRP3 inflammasome and enhancing microglial autophagy.

### Autophagy antagonist blocks the pharmacologic effects of formononetin on microglial M1/M2 polarization and NLRP3 inflammasome in BV2

**Fig. 3:**
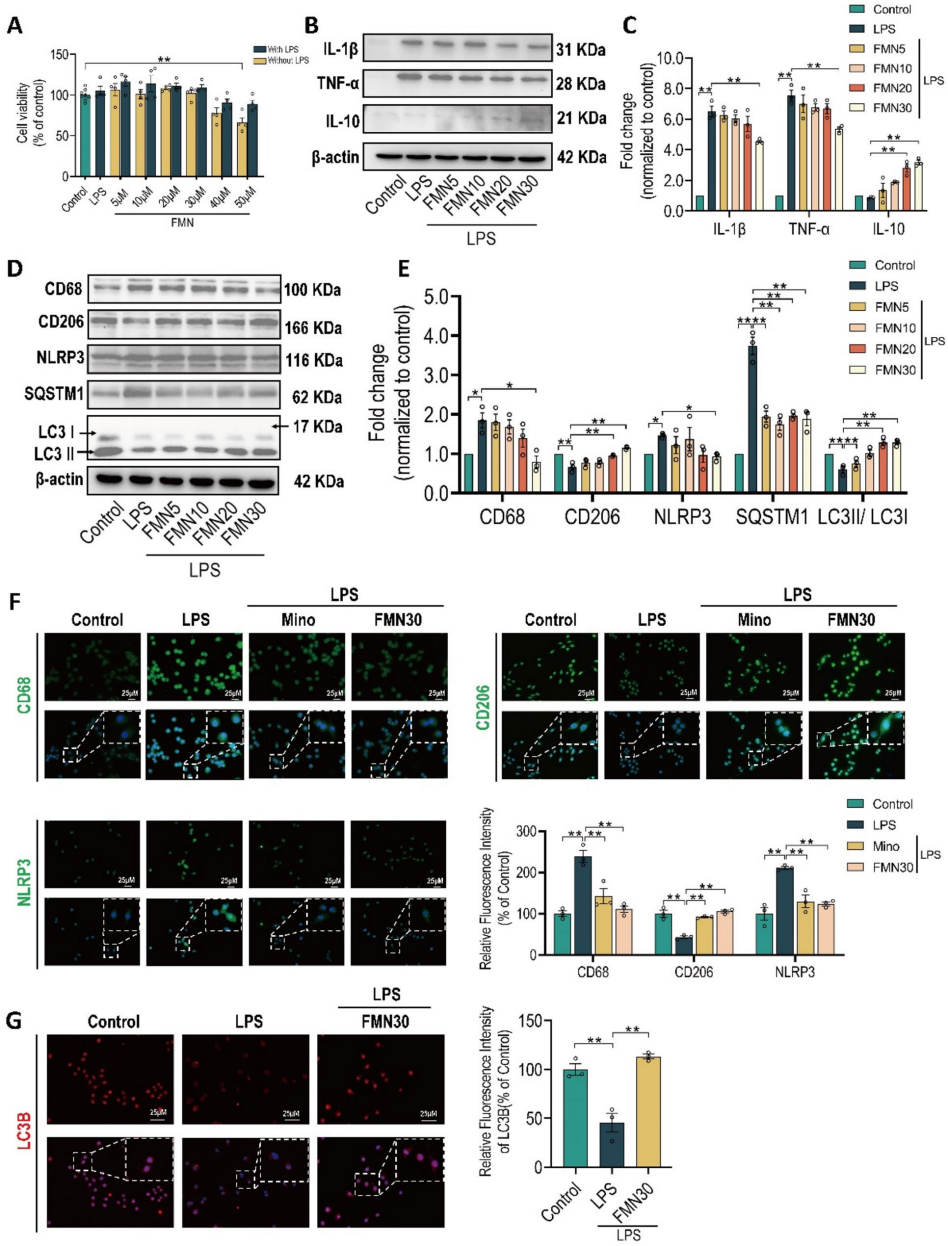
Pharmacologic effects of formononetin in BV2. BV2 cells were pre-treated with FMN and positive control minocycline (Mino, 20 µM) for 1 h and then incubated with or without LPS (1 µg/mL) for 24 h. **A** Cell viability of BV2 pre-incubated with different concentrations of FMN with or without LPS stimulation, was determined by CCK8 assay, *n* = 4 independent repetitions. **B**,** C** Quantification of protein expression levels of TNF-α, IL1-β and IL10, *n* = 3. **D**,** E** Quantification of protein expression levels of CD68, CD206, NLRP3, SQSTM1 and LC3II/I, *n* = 3. **F**,** G** Representative immunofluorescence images for respectively staining with CD68, CD206, NLRP3 and LC3B in BV2, and immunofluorescence intensity analysis for corresponding protein marker, Scale bar: 25 μm, *n* = 3. The bar graphs were represented as mean ± SEM. * *P* < 0.05, ** *P* < 0.01

In order to uncover the underlying connections among these pharmacologic effects of FMN, we firstly used typical microglia cell line BV2 to further assess pharmacologic effect of FMN in *vitro* model induced by LPS (1 µg/mL). The cell viability of different concentrations of FMN under condition with or without LPS stimulation were detected by CCK-8 assay, to scan the suitable concentration. As shown in Fig. 3A, the concentrations within 30 µM of FMN all exerted no cytotoxicity to cells with or without LPS. FMN could significantly inhibit the protein expression of TNF-α and IL1-β while increase IL10 (Figs. 3B, C). Moreover, FMN remarkably suppressed the protein expression of CD68 and NLRP3, while increased CD206 (Figs. 3D, E). In immunofluorescence assays (Fig. 3F), FMN also significantly decreased the fluorescence intensity of CD68 and NLRP3 and increased the fluorescence intensity of CD206. In addition, FMN could prominently decrease the protein expression of SQSTM1 and increase the ratio of LC3II/I (Figs. 3D, E), and also increase the fluorescence intensity of LC3B (Fig. 3G). These findings indicated that FMN also exhibits pharmacologic effects on rebalancing M1/M2 polarization, inhibiting NLRP3 inflammasome and enhancing autophagy level in BV2.

**Fig. 4:**
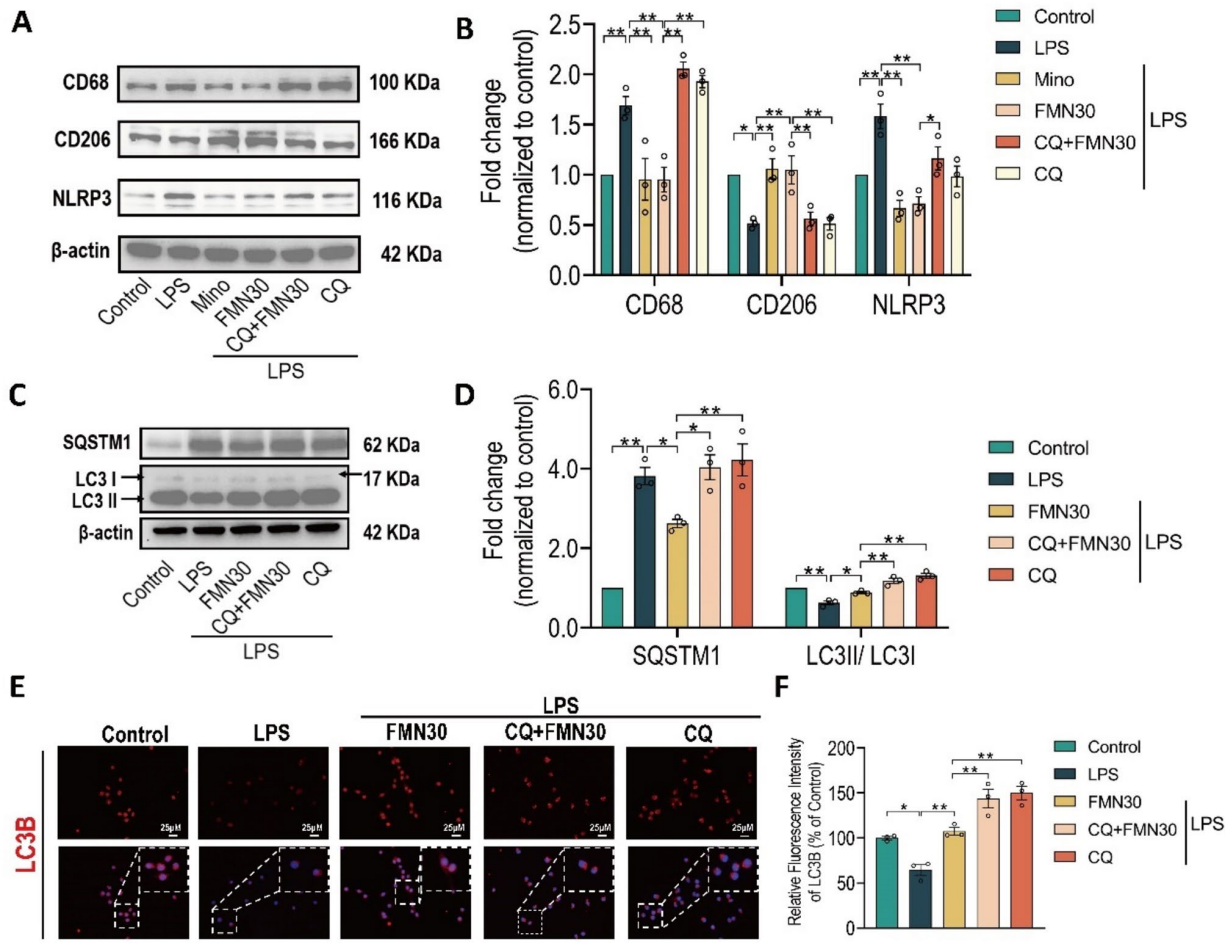
Autophagy antagonist blocks the pharmacologic effects of formononetin in BV2. BV2 cells were pre-treated with FMN (30 µM), chloroquine (CQ, 20 µM) and minocycline (Mino, 20 µM) for 1 h and then incubated with LPS (1 µg/mL) for 24 h. **A**,** B** Quantification of protein expression levels of CD68, CD206 and NLRP3, *n* = 3. **C**,** D** Quantification of protein expression levels of SQSTM1 and LC3II/I, *n* = 3. **E**,** F** Representative immunofluorescence images for staining with LC3B in BV2 pre-incubated with indicated drugs, and immunofluorescence intensity analysis for LC3B, Scale bar: 25 μm, *n* = 3. The bar graphs were represented as mean ± SEM. * *P* < 0.05, ** *P* < 0.01

Next, to determine whether the effects of FMN on rebalancing M1/M2 polarization and inhibiting NLRP3 inflammasome are dependent on enhancing autophagy, we simultaneously pre-incubated autophagy antagonist chloroquine (CQ) with FMN before LPS stimulation. As shown in Figs. 4A, B, the pharmacologic effects of FMN on CD68, NLRP3 and CD206 were significantly blocked by pre-incubation of CQ. The immunofluorescence assays with intensity analysis still exerted the similar effects (Fig. S1). In addition, the analysis of protein expression (SQSTM1 and LC3II/I) (Figs. 4C, D) and fluorescence intensity (LC3B) (Figs. 4E, F) both showed that CQ also blocks the effect of FMN on enhancing autophagy. Above results confirmed that FMN rebalances microglia polarization and inhibits NLRP3 inflammasome via promoting autophagy.

### PPARα is identified as a target of formononetin

**Fig. 5:**
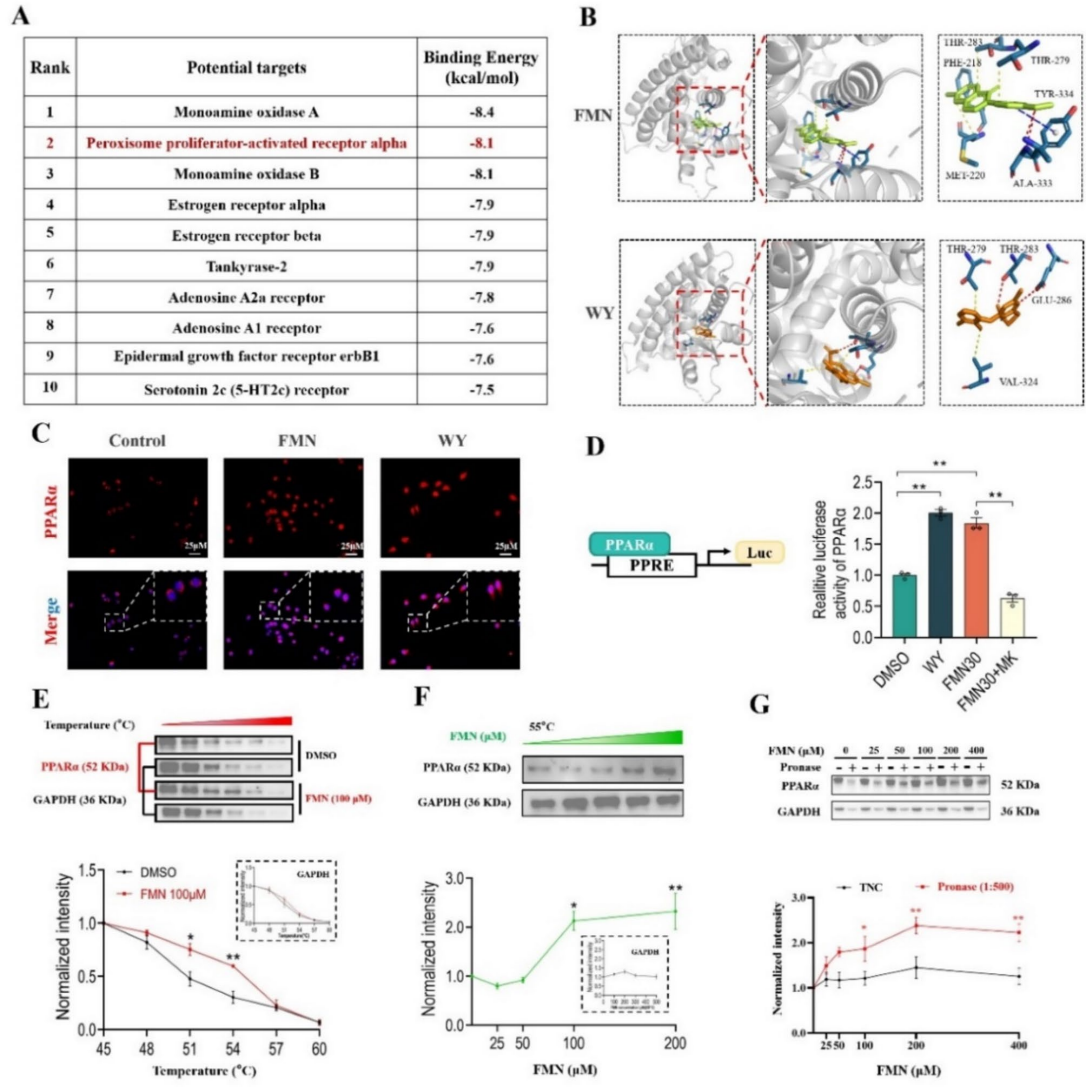
PPARα was identified as a target of formononetin. **A** Ranking of molecular docking between FMN and its potential targets. **B** Molecular docking of FMN and WY14643 with PPARα (LI7G). Red line: hydrogen bonds; blue line: Pi-stacking bonds; yellow line: hydrophobic bonds. **C** Immunofluorescence staining of PPARα in BV2 cells pre-treated with FMN (30 µM) or WY14643 (WY, 20 µM). Scale bar: 25 μm. **D** Luciferase reporter assay showing PPARα activity. HEK293T cells were co-transfected with full-length PPARα and PPRE-J3-TKluc reporter, followed by treatment with FMN (30 µM), WY14643 (1 µM), or MK886 (MK, 30 µM). **E** Thermal stability of PPARα in the presence or absence of FMN. **F** Thermal stability of PPARα at 55℃ with increasing FMN concentrations. The inner figures are the qualification analysis of GAPDH in E and F. **G** Pronase resistance of PPARα with different concentrations of FMN (1:500 pronase). The bar graphs were represented as mean ± SEM, *n* = 3 independent repetitions. * *P* < 0.05, ** *P* < 0.01

To identify the direct target of FMN, we firstly carried out the structure-based virtual screening using Swiss-Target-Prediction to predict the potential direct molecular targets of FMN, and the top twenty candidates were listed in Fig. S2. Then, these candidates were subjected to molecular docking with FMN using AutoDock and Pymol, then the top ten binding energies were ranked in Fig. 5A. The peroxisome proliferator-activated receptor-α (PPARα) attracted our attentions because of its higher binding energy and crucial roles in regulation of microglial polarization (Li et al. 2023) and NLRP3 inflammasome inhibition (Marin-Aguilar et al. 2020). Importantly, molecular docking with PPARα showed that PPARα agonist (WY14643) binds the similar regions to the FMN, and also has the similar binding amino acid residues (Fig. 5B). Moreover, the immunofluorescence assays showed that FMN and WY14643 both promote nuclear translocation of PPARα (Fig. 5C). We used the luciferase reporter system containing PPARα binding element to further investigate the effect of FMN on nuclear translocation of PPARα in HEK293T cells. As shown in the Fig. 5D, the WY14643 and FMN both significantly increased the luciferase activity and the increase could be blocked by PPARα selective antagonist (MK-886). Additionally, FMN increased the thermal stability of PPARα (Figs. 5E, F) and enhanced its resistance to pronase in a concentration-dependent manner (Fig. 5G). Taken together, these findings suggested that PPARα is a direct target of FMN.

### The roles of PPARα in pharmacologic effects of formononetin in BV2

**Fig. 6:**
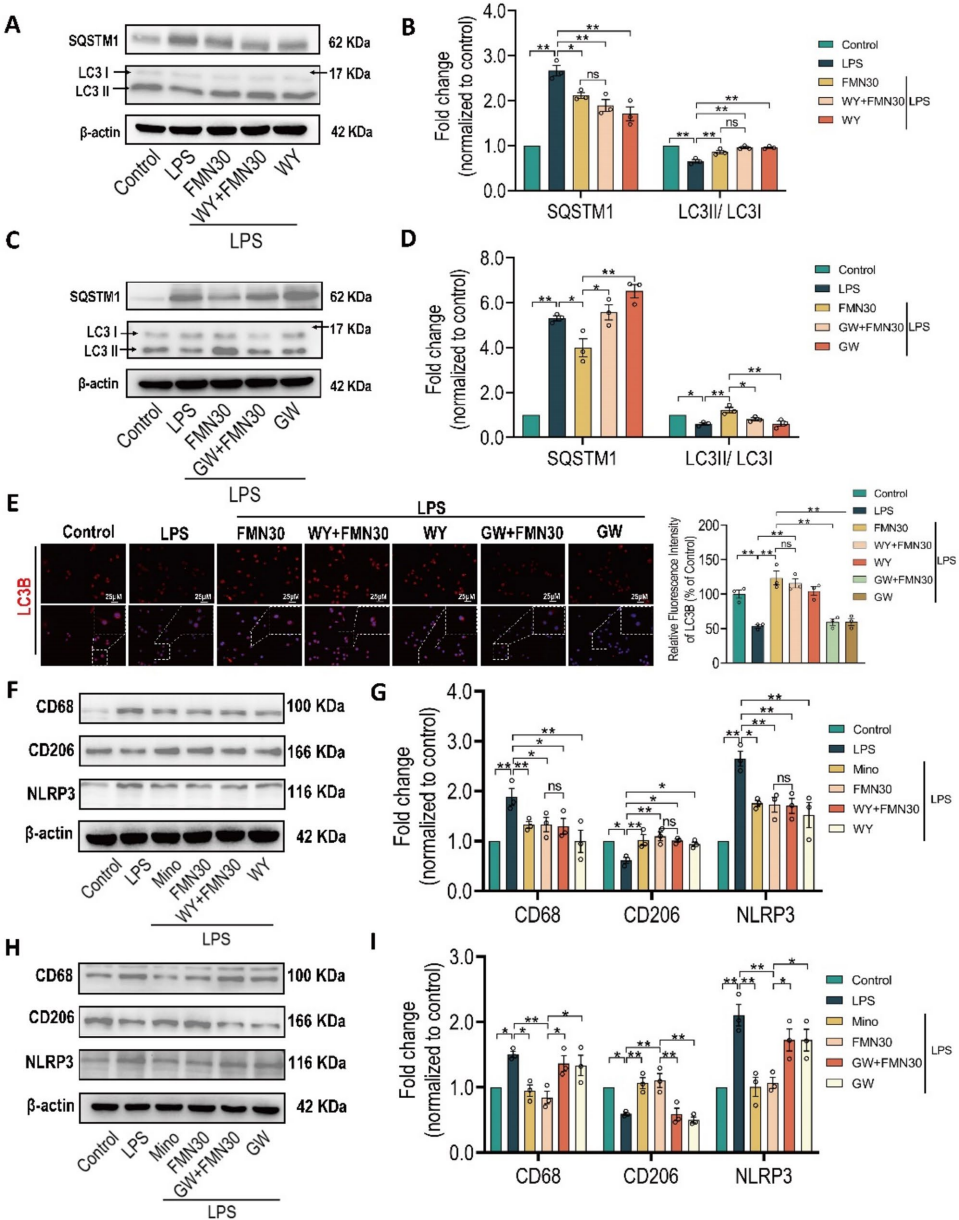
Role of PPARα agonist/antagonist in pharmacologic effects of formononetin. BV2 cells were pre-treated with FMN (30 µM), minocycline (Mino, 20 µM), WY14643 (WY, 20 µM) and GW6471 (GW, 5 µM) for 1 h and then incubated with LPS (1 ug/mL) for 24 h. Quantification of protein expression levels of SQSTM1 and LC3II/I in BV2 with simultaneous pre-incubation with WY and FMN (**A**,** B**), GW and FMN (**C**,** D**), *n* = 3. **E** Representative immunofluorescence images for staining with LC3B in BV2 pre-incubated with indicated drugs, and immunofluorescence intensity analysis, Scale bar: 25 μm, *n* = 3. Quantification of protein expression levels of CD68, CD206 and NLRP3 in BV2 with simultaneously pre-incubation with WY and FMN (**F**,** G**), GW and FMN (**H**,** I**), *n* = 3. The bar graphs were represented as mean ± SEM. * *P* < 0.05, ** *P* < 0.01, n.s., not significant

Due to above data indicated that the effects of FMN on rebalancing microglia polarization and inhibiting NLRP3 inflammasome are dependent on enhancement of autophagy, so the following question is whether FMN promotes autophagy via directly activating PPARα, to determine the roles of PPARα in effect of FMN on autophagy, the PPARα agonist (WY14643) or PPARα antagonist (GW6471) were simultaneously pre-incubated with FMN before LPS stimulation in BV2. As shown in Figs. 6A, B, FMN and WY both decreased the protein expression of SQSTM1 and increased the ratio of LC3II/I to enhanced autophagic level. Importantly, simultaneous pre-incubation with WY14643 and FMN had no significant additive effect on enhancement of autophagic level (Figs. 6A, B). Moreover, pre-incubation of GW6471 remarkably blocked the effect of FMN on enhancement of autophagic level (Figs. 6C, D). In addition, the immunofluorescence assays also showed that WY14643 along with FMN has no additive effect on increase of LC3B intensity, but GW6471 blocks the increase of LC3B intensity by FMN (Fig. 6E). These results overwhelmingly supported that enhancement of microglia autophagic level by FMN is dependent on directly activating PPARα.

As shown in Figs. 6F-G, FMN and WY both decreased the protein expression of CD68, NLRP3 while increased CD206. Interestingly, simultaneous pre-incubation with WY14643 and FMN still had no significant additive effect (Figs. 6F-G). Moreover, pre-incubation of GW6471 remarkably blocked the pharmacologic effects of FMN on regulation of microglia polarization and NLRP3 inflammasome (Figs. 6H, I). Furtherly, the immunofluorescence assays also showed that pre-incubation of WY14643 or GW6471 along with FMN has similar results to western blot (Fig. S3). Taken together, our results overwhelmingly supported that FMN rebalances microglia M1/M2 polarization and inhibits NLRP3 inflammasome via directly activating PPARα-mediated autophagy.

### PPARα antagonist abolishes the antidepressant effect of formononetin in LPS-induced depressive mice

**Fig. 7:**
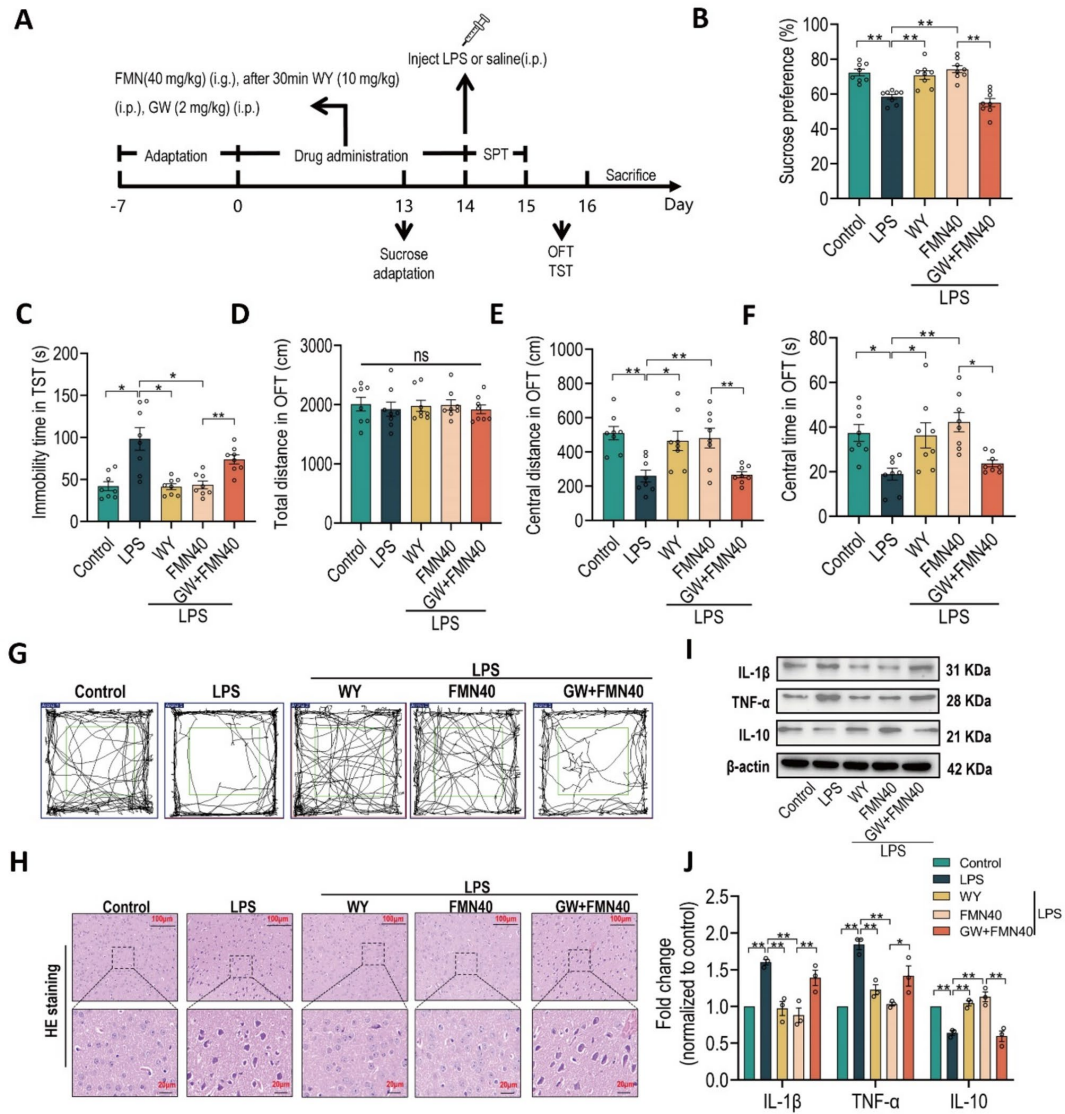
PPARα antagonist abolishes the antidepressant effect of formononetin in LPS-induced depressive mice. **A** Schematic diagram of experimental protocols with 8 mice in each group. During the drug administration, FMN group and GW + FMN group were given by intragastric (i.g.) administration with FMN (40 mg/kg) once a day, other groups (Control, LPS and WY) all were given by intragastric administration with pure water. After 30 min, WY and GW + FMN groups were intraperitoneally (i.p.) injected with WY14643 (10 mg/kg) and GW6471 (2 mg/kg) respectively once a day, other groups (Control, LPS and FMN) all were intraperitoneally injected with saline. Then after the last time drug administration, excepting control group injecting with saline, other groups were all intraperitoneally injected with LPS (1 mg/kg). **B** The sucrose preference in SPT. **C** The immobility time in TST. The total distance (**D**), central distance (**E**) and central time (**F**) in central area (green frame) in OFT. **G** The representative moving track in OFT. **H** Representative images of histological neuronal injury in prefrontal cortex detected by H&E staining (scale bar, 100 μm), *n* = 3 sections from three mice. **I**,** J** Quantification of protein expression levels of TNF-α, IL1-β and IL10 in prefrontal cortex, *n* = 3. The bar graphs were represented as mean ± SEM, B-F with 8 mice in each group * *P* < 0.05, ** *P* < 0.01, n.s., not significant

To further validate the roles of PPARα in antidepressant effect of FMN, we treated PPARα antagonist GW6471 along with FMN (40 mg/kg, the more effective dose) in LPS-induced depressive mice, then carried out SPT, OFT, and TST to assess depression-like behaviors (Fig. 7A). As shown in Fig. 7B, the treatment of FMN and positive control WY14643 (10 mg/kg, PPARα agonist) both significantly recovered the decreased of sucrose preference induced by LPS challenge. Moreover, FMN and WY14643 both remarkably reversed the increase of immobility time in TST (Fig. 7C) and the decrease of central distance and central time in OFT (Figs. 7D-G). Importantly, compared with individual FMN, treatment of GW6471 (2 mg/kg) along with FMN significantly decreased sucrose preference in SPT (Fig. 7B), increased immobility time in TST (Fig. 7C) and decreased central distance and central time in OFT (Figs. 7D-G). In addition, the H&E staining exhibited that neurological injury in PFC induced by LPS can be significantly alleviated by FMN and WY14643 treatment, but GW6471 blocked the effect of FMN (Fig. 7H). The increase of IL10 and decrease of TNF-α and IL1-β also were blocked by GW6471 (Figs. 7I-J). Overall, these findings indicated that the antidepressant effects and alleviation of neuroinflammation of FMN are prominently abolished by treatment GW6471 in LPS-induced depressive mice.

### PPARα antagonist abrogates the pharmacologic effects of formononetin in prefrontal cortex

**Fig. 8:**
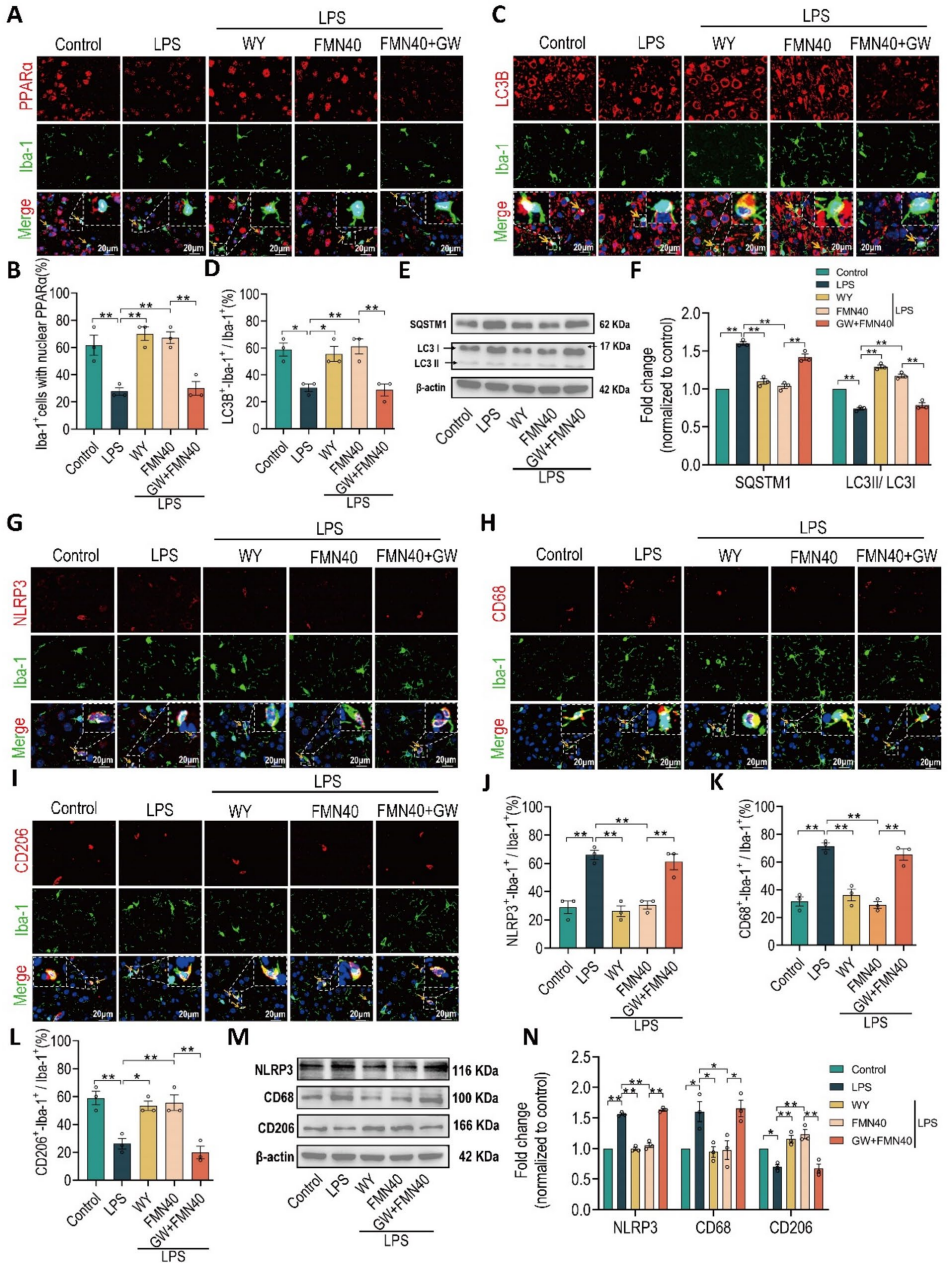
PPARα antagonist abrogates the pharmacologic effects of formononetin in prefrontal cortex. **A**,** C** Representative immunofluorescence images co-staining with Iba-1 (Green) and PPARα (Red) or LC3B (Red), scale bar: 20 μm, the inner images were magnified from the corresponding white dotted frame regions, the yellow arrows represent the microglia co-expressing with LC3B or nuclear translocation of PPARα. **B**,** D** Quantification of the percentage of Iba-1^+^/PPARα^+^ cells or Iba-1^+^/LC3B^+^ cells among Iba-1^+^ cells, *n* = 3. **E**,** F** Quantification of protein expression levels of the SQSTM1, LC3II/I, *n* = 3. **G-I** Representative immunofluorescence images co-staining with Iba-1 (Green) and NLRP3, CD68 (Red) or CD206 (Red), scale bar: 20 μm, the inner images were magnified from the corresponding white dotted frame regions, the yellow arrows represent the microglia co-expressing NLRP3, M1 or M2 polarization microglia. **J-L** Quantification of the percentage of Iba-1^+^/NLRP3^+^ cells, Iba-1^+^/CD68^+^ cells or Iba-1^+^/CD206^+^ cells among Iba-1^+^ cells, *n* = 3. **M**,** N** Quantification of protein expression levels of the NLRP3, CD68 and CD206, *n* = 3. The bar graphs were represented as mean ± SEM. * *P* < 0.05, ** *P* < 0.01

Above data indicated that the pharmacologic effects of FMN are dependent on activating PPARα in BV2, therefore, to further validate the roles of PPARα in pharmacologic effect of FMN in prefrontal cortex of LPS-induced depressive mice, we also treated PPARα antagonist GW6471 (2 mg/kg) along with FMN (40 mg/kg) to detect corresponding pharmacologic indexs in *vivo*. As shown in Figs. 8A, B, WY14643 (10 mg/kg) and FMN both promoted microglia nuclear translocation of PPARα, whereas GW6471 blocked the promotion of PPARα nuclear translocation of FMN in prefrontal cortex. Immunofluorescence data of prefrontal cortex staining with LC3B (Figs. 8C, D) indicated that GW6471 significantly blocks the enhancement of microglia autophagy by FMN. Compared with individual FMN, the data of protein expression of SQSTM1 and LC3II/I indicated that GW6471 along with FMN also decrease the microglia autophagy level in prefrontal cortex (Figs. 8E-F). Moreover, immunofluorescence data of prefrontal cortex staining with NLRP3 (Figs. 8G, J), CD68 (Figs. 8H, K) and CD206 (Figs. 8I, L) also indicated that GW6471 abrogates the effects of FMN on inhibiting NLRP3 inflammasome, decreasing microglia M1 polarization and increasing microglia M2 polarization in prefrontal cortex. In addition, compared with individual FMN, GW6471 along with FMN also decreased protein expression of CD206 while increased NLRP3 and CD68 (Figs. 8M, N). Taken together, these findings overwhelmingly demonstrated that PPARα antagonist prominently abrogates pharmacologic effects of FMN on inhibiting NLRP3 inflammasome and rebalancing microglia M1/M2 polarization in PFC of LPS-induced depressive mice.

## Discussion

Until now, clinical antidepressants, including selective serotonin reuptake inhibitors (SSRIs), monoamine oxidase inhibitors (MAOIs) and tricyclic antidepressants (TCAs), were initially discovered by modulating monoamine neurotransmission (Wong and Licinio 2004). However, numerous researches have reported that only one-third of depressive patients have beneficial treatment response to these antidepressants complicating by serious side-effects of nausea, vomiting, sleep dysregulation and sexual dysfunction (Wang et al. 2019). Therefore, seeking safer and more effective alternatives is an urgent demand for treatment of depression. In recent years, the therapeutic properties of Traditional Chinese Medicine (TCM), including compound Chinese medicine and major bioactive components, have attracted considerable attention in treating depression (Feng et al. 2022). Formononetin (FMN), is especially rich in Traditional Chinese Medicine *Glycyrrhiza uralensis* and *Astragalus membranaceus*, and has attracted more and more attention because of its anti-inflammatory and neuroprotective effects (Tian et al. 2022). In present study, we aimed to assess the antidepressive effect of FMN and deeply investigate its antidepressive mechanisms in LPS-induced depressive model.

Recent years, the alleviation of neuroinflammation could ameliorate depressive-like behaviors via various mechanisms has been demonstrated by accumulating evidence (Guo et al. 2023; Nettis and Pariante 2020). Moreover, as an endotoxin isolated from bacteria, the peripheral LPS challenge can elicit liable and persistent neuroinflammation, and be frequently used to construct neuroinflammation-induced depressive model (Zhao et al. 2019). Due to paroxetine can alleviate neuroinflammation to ameliorate LPS-induced depression-like behaviors (Ito et al. 2025; Ohgi et al. 2013; Su et al. 2024; Yao et al. 2015), so we used the paroxetine as the positive control in this study. Our data showed the injection of LPS also induced remarkable depression-like behaviors and neuroinflammatory neuronal damage, whereas FMN and paroxetine administration both significantly alleviated neuroinflammation of prefrontal cortex and ameliorated depression-like behaviors. In addition, FMN also decreased pro-inflammatory cytokines and increased anti-inflammatory cytokine. We also detected the microglia polarization because of its critical roles in regulation of neuroinflammation-induced depression (Jia et al. 2021). The present data showed that FMN can rebalance microglia M1/M2 polarization in prefrontal cortex and BV2. The NLRP3 inflammasome is sensitive to psychological stressors and is a crucial contributor for regulation of microglia polarization (Jie et al. 2022; Wang et al. 2022; Zhang et al. 2022b). The results showed FMN can significantly inhibit NLRP3 inflammasome activation in prefrontal cortex and BV2. Indeed, some antidepressants also exhibited the ability of rebalancing M1/M2 polarization and suppressing NLRP3 inflammasome in depressive model mice (Alcocer-Gomez et al. 2017; Jia et al. 2021). Taken together, our data supported that FMN exerted antidepressant efficacy via rebalancing microglia M1/M2 polarization and inhibiting NLRP3 inflammasome activation.

Due to the critical roles of autophagy in modulation of microglia M1/M2 polarization and NLRP3 inflammasome (Kang et al. 2024), the microglia autophagy attracted more and more attention to some investigations involving in antidepressant mechanisms (Tang et al. 2021; Xia et al. 2023). Our results indicated that FMN also improves microglia autophagy in prefrontal cortex and BV2. Importantly, the autophagy inhibitor chloroquine (CQ) significantly blocked the effect of FMN on rebalancing microglia M1/M2 polarization and inhibiting NLRP3 inflammasome in BV2. In fact, promotion of microglia autophagy exactly reversed depression-like behaviors in various depressive model (Su et al. 2023; Tan et al. 2018). These data suggested that FMN rebalancing microglia M1/M2 polarization and inhibiting NLRP3 inflammasome activation via improving autophagy.

Furtherly, we attempted to find out how FMN improves microglia autophagy to exert its pharmacologic effects. Firstly, PPARα was identified as the direct target of FMN via various methods. Considering of the key roles of PPARα in regulation of autophagy through modulating expression of autophagy-related genes, we deeply investigated whether FMN directly activates PPARα to improve autophagy. Our results showed that PPARα antagonist significantly blocks the effect of FMN on enhancing autophagy in BV2. Moreover, the PPARα antagonist also abrogated the effects of FMN on rebalancing microglia M1/M2 polarization and inhibiting NLRP3 inflammasome in BV2. In fact, activation of PPARα can modulate microglia inflammatory responses have been reported in some studies (Li et al. 2023; Marin-Aguilar et al. 2020; Xiong et al. 2024). Importantly, our data showed that the PPARα antagonist still blocks the effects of FMN on suppressing neuroinflammation, rebalancing microglia polarization, inhibiting NLRP3 inflammasome and improving autophagy to abolish antidepressant efficacy of FMN in LPS-induced depressive mice. In addition, we found that the effect of FMN on PPARα in PFC not just in microglia, suggested that FMN also can regulate PPARα in other cell types. Actually, besides neuroinflammation, the PPARα acts key roles in regulation of lipid metabolism, astrocyte also plays critical roles in development of depression via regulation of lipid metabolism (Huang et al. 2017; Lam et al. 2021). So, whether FMN possesses other mechanisms for antidepressant effect need to further investigate. Collectively, our results overwhelmingly supported that FMN exerts its pharmacologic effects via directly activating PPARα-mediated autophagy.

## Conclusions

**Fig. 9:**
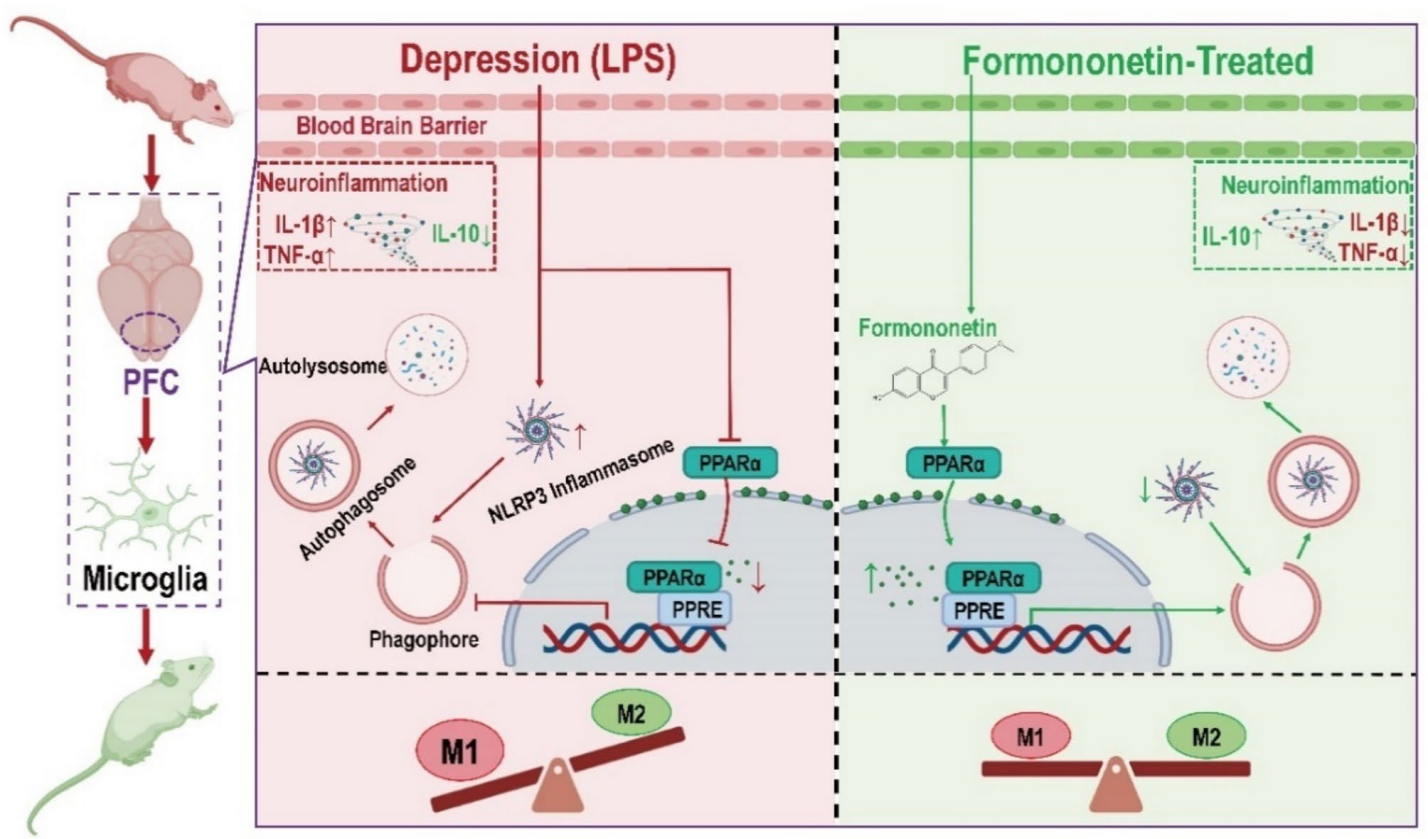
Proposed antidepressant mechanism of formononetin (FMN) in LPS-induced depressive model. In depression state (LPS stimulation), NLPR3 inflammasome was excessively activated, and the inhibition of NLRP3 inflammasome depended on microglia autophagy was weakened because of suppression of autophagy level mediated by translocation of PPARα, then leading to imbalance microglia M1/M2 polarization, further resulting in dysregulation of neuroinflammation in prefrontal cortex, thereby inducing depression-like behaviors. In FMN-treated state, FMN promoted translocation of PPARα and directly activated PPARα-mediated autophagy to inhibit NLRP3 inflammasome and rebalance microglia M1/M2 polarization, then restore homeostasis of neuroinflammation to exhibit antidepressant efficacy

In conclusion, we identified PPARα as a direct target of FMN, and our data firstly indicated that FMN ameliorates LPS-induced depression-like behaviors through rebalancing microglia M1/M2 polarization and inhibiting NLRP3 inflammasome, with involvement of activating PPARα-mediated autophagy (Fig. 9). Overall, this study reveals a novel antidepressant mechanism of FMN in terms of neuroinflammation regulated by microglia polarization and NLRP3, and also provides a potential therapeutic target for depression treatment.

## Electronic supplementary material

Below is the link to the electronic supplementary material.

Supplementary Material 1

Supplementary Material 2

Supplementary Material 3

Supplementary Material 4

## Data Availability

The raw data materials related to this study are available from the corresponding author upon reasonable request.

## Abbreviations

NLRP3: Nucleotide-binding domain, Leucine-rich Repeat, and Pyrin domain (PYD)-containing Protein 3
FMN: Formononetin
CQ: Chloroquine
LPS: Lipopolysaccharide
PPARα: Peroxisome proliferator-activated receptor-α
DMSO: Dimethyl sulfoxide
CORT: Corticosterone
CCK-8: Cell counting kit-8
CD68: Cluster of differentiation 68
CD206: Cluster of differentiation 206
LC3B: Microtubule-associated protein 1 A/1B-light chain 3B
SQSTM1: Sequestosome-1
IL-1β: Interleukin-1β
TNF-α: Tumor necrosis factor-α
IL-10: Interleukin-10
SPT: Sucrose preference test
OFT: Open field test
TST: Tail suspension test
H&E: staining Hematoxylin-eosin staining
PFC: Prefrontal cortex
WB: Western blot
IF: Immunofluorescent
CETSA: Cellular thermal shift assay
GAPDH: Glyceraldehyde-3-phosphate dehydrogenase
SSRIs: Selective serotonin reuptake inhibitors
MAOIs: Monoamine oxidases inhibitors
TCAs: Tricyclic antidepressants
TCM: Traditional Chinese Medicine
