# Supplementary material for: Formononetin ameliorates depression-like behaviors through rebalancing microglia M1/M2 polarization and inhibiting NLRP3 inflammasome: involvement of activating PPARα-mediated autophagy
Source: Mol Med. 2025 Apr 24;31:153. doi: 10.1186/s10020-025-01217-2 (PMC12023581; doi:10.1186/s10020-025-01217-2)
Supplement: Supplementary file 1 — Supplementary Material 1 [file 10020_2025_1217_MOESM1_ESM.docx]

**Supplementary Figures**

**Formononetin ameliorates depression-like behaviors through rebalancing microglia M1/M2 polarization and inhibiting NLRP3 inflammasome: involvement of activating PPARα-mediated autophagy**

Shuaijun Peng^1,3,#^, Pan Su^1,2,#,*^, Liming Liu^1,3^, Zibo Li^1,2^, Yuan Liu ^1,3^, Lei Tian^1,2^, Ming Bai^1,2^, Erping Xu^1,2,*^, Yucheng Li^1,2,*^

^1^Collaborative Innovation Center of Research and Development on the Whole Industry Chain of Yu-Yao, Henan Province; Henan University of Chinese Medicine, Zhengzhou 450046, P.R. China

^2^Academy of Chinese Medical Sciences, Henan University of Chinese Medicine, Zhengzhou 450046, P.R. China

^3^College of Pharmacy, Henan University of Chinese Medicine, Zhengzhou 450046, PR China

^#^ These authors contributed equally to this work, E-mail addresses:

[ShuaijunPeng1214@126.com](mailto:ShuaijunPeng1214@126.com), [Supan@hactcm.edu.cn](mailto:Supan@hactcm.edu.cn)

*Corresponding authors at: Academy of Chinese Medical Sciences, Henan University of Chinese Medicine, Zhengzhou 450046, P.R. China, E-mail addresses: [Supan@hactcm.edu.cn](mailto:Supan@hactcm.edu.cn), [Xuerping0371@163.com](mailto:Xuerping0371@163.com), [Liyucheng@hactcm.edu.cn](mailto:Liyucheng@hactcm.edu.cn)

**Supplementary Figure-1**


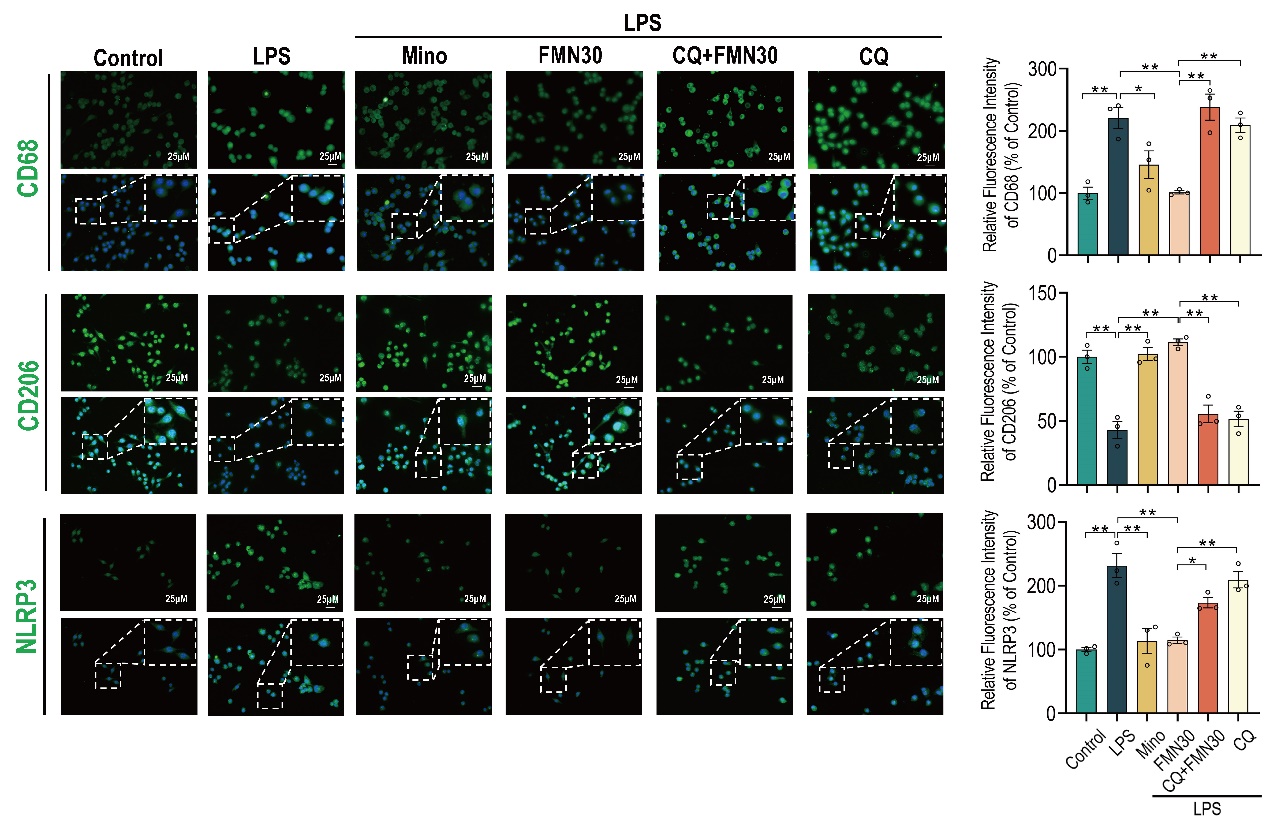


**Fig. S1.** Immunofluorescence analysis for the effect of autophagy antagonist on pharmacologic effects of formononetin. BV2 cells were pre-treated with FMN (30 μM), chloroquine (CQ, 20 μM) and minocycline (Mino, 20 μM) for 1 h and then incubated with LPS (1 μg/mL) for 24 h. Representative immunofluorescence images for respectively staining with CD68, CD206 and NLRP3 in BV2 pre-incubated with indicated drugs, and immunofluorescence intensity analysis for corresponding marker, Scale bar: 25 μm, n=3. The bar graphs were represented as mean ± SEM. * *P* < 0.05, ** *P* < 0.01.

**Supplementary Figure-2**


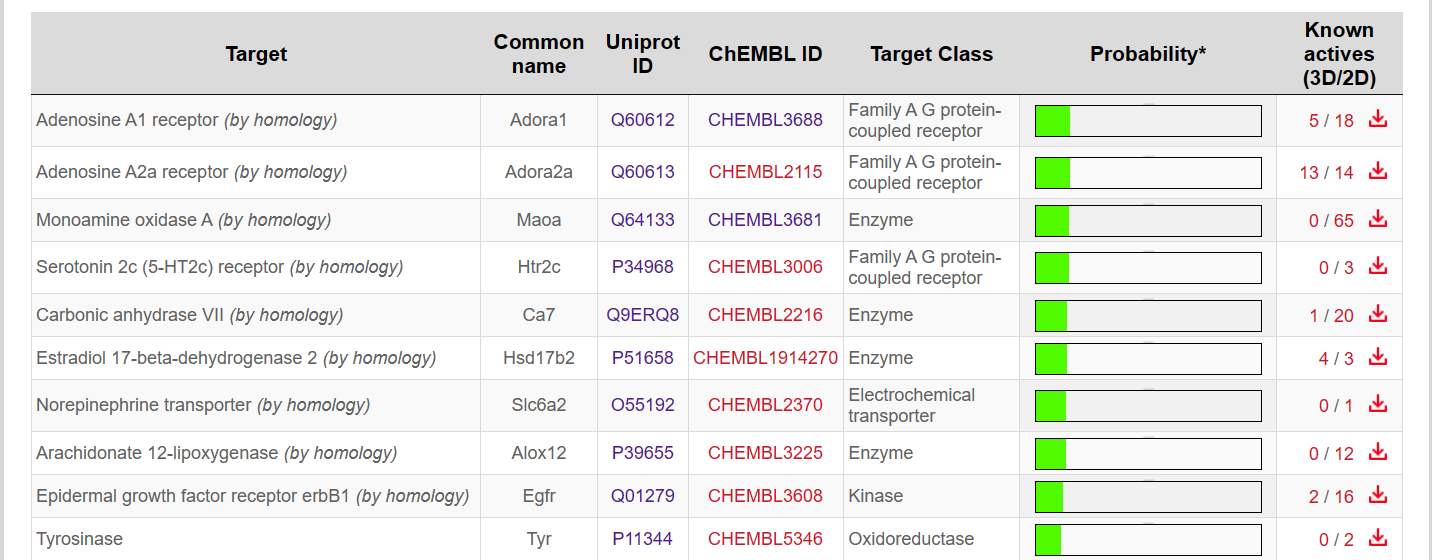

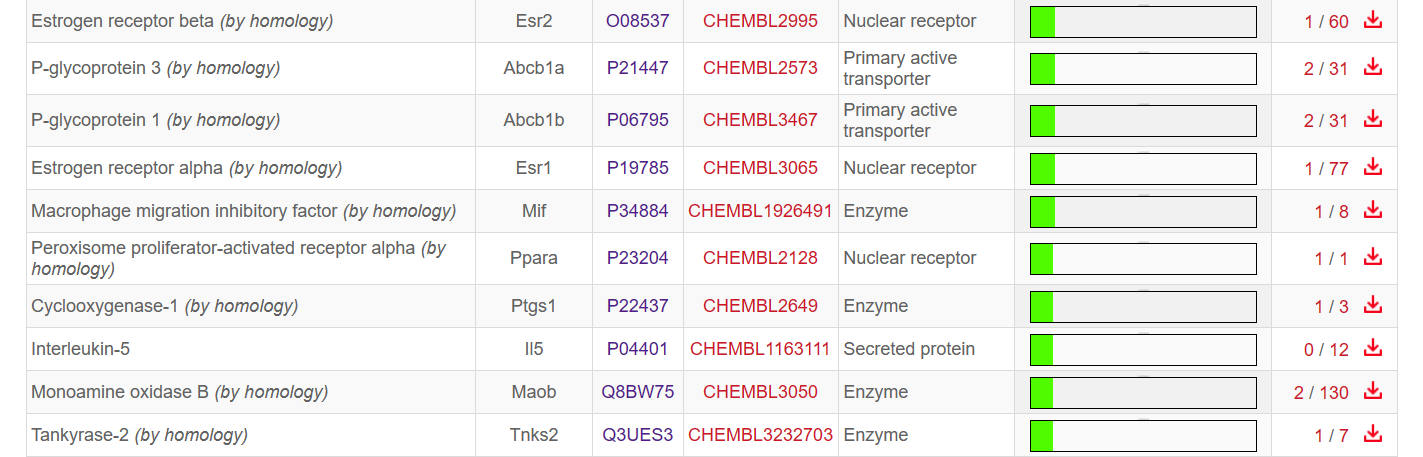


**Fig. S2.** Top 20 potential targets of FMN analyzed using the Swiss-Target-Prediction tool (<http://swisstargetprediction.ch>).

**Supplementary Figure-3**


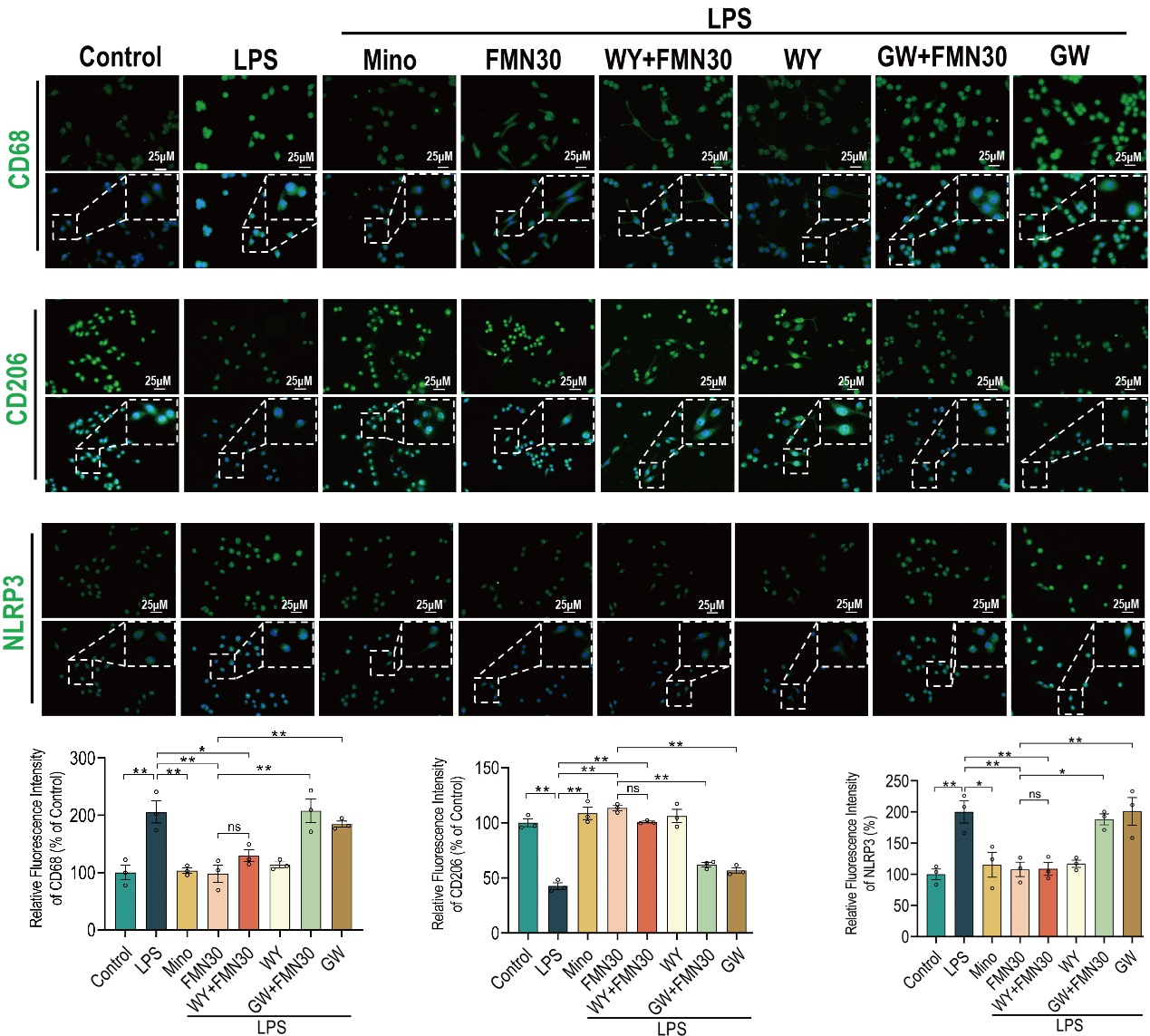


**Fig. S3.** Immunofluorescence analysis for the effect of PPARα agonist/antagonist on pharmacologic effects of formononetin. BV2 cells were pre-treated with FMN (30 μM), minocycline (Mino, 20 μM), WY14643 (WY, 20 μM) and GW6471 (GW, 5 μM) for 1 h and then incubated with LPS (1 ug/mL) for 24 h. Representative immunofluorescence images for respectively staining with CD68, CD206 and NLRP3 in BV2 pre-incubated with indicated drugs, and immunofluorescence intensity analysis for corresponding marker, Scale bar: 25 μm, n=3. The bar graphs were represented as mean ± SEM. * *P* < 0.05, ** *P* < 0.01, n.s., not significant.
