# Supplementary material for: Formononetin ameliorates depression-like behaviors through rebalancing microglia M1/M2 polarization and inhibiting NLRP3 inflammasome: involvement of activating PPARα-mediated autophagy
Source: Mol Med. 2025 Apr 24;31:153. doi: 10.1186/s10020-025-01217-2 (PMC12023581; doi:10.1186/s10020-025-01217-2)

Fig1 L

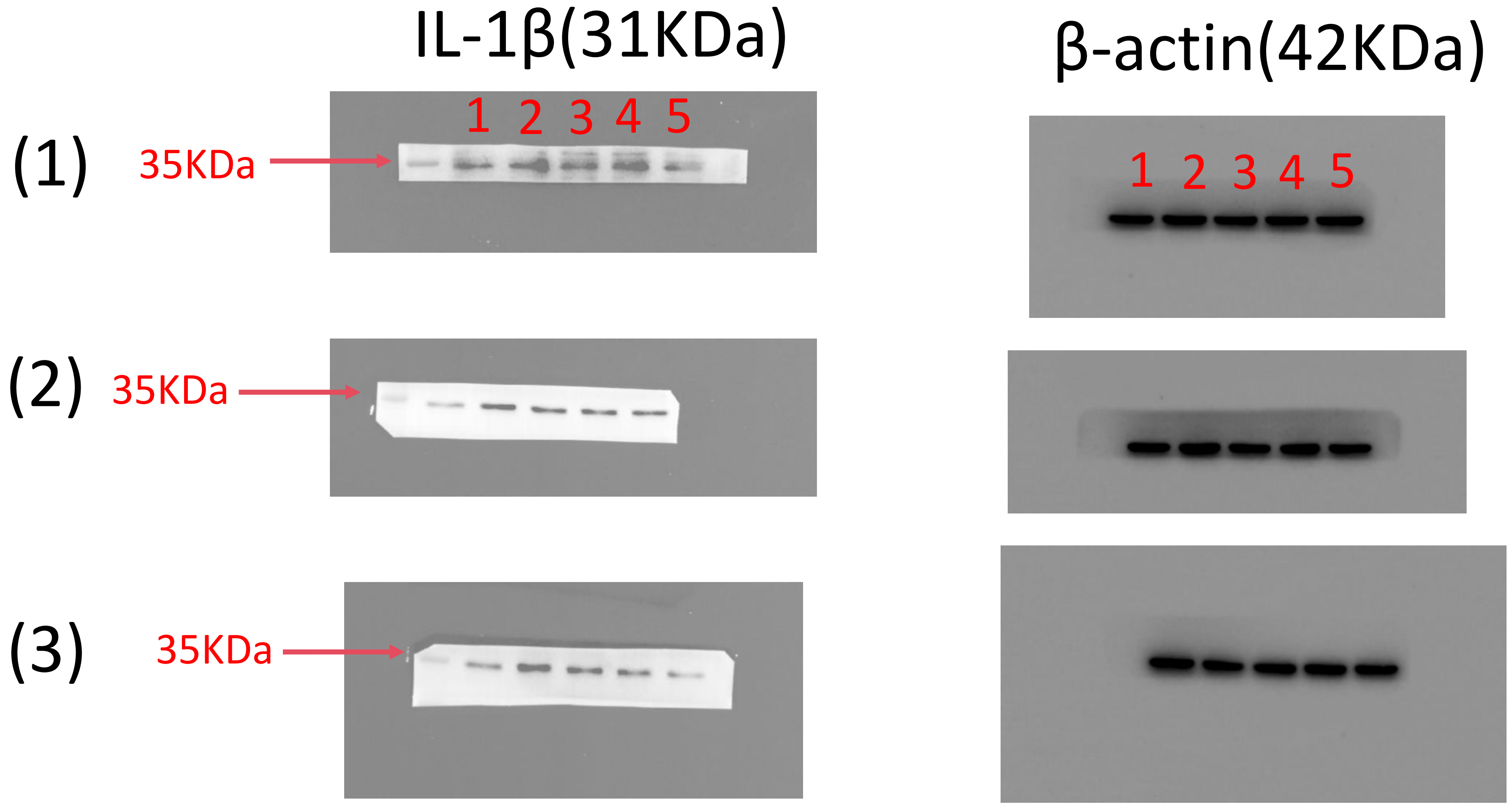

1: Control
2: LPS
3: LPS+Paroxetine (20mg/kg)
4: LPS+FMN(20mg/kg)
5: LPS+FMN(40mg/kg)

The protein band marker(PR1920) was purchased from the Solarbio (Beijing, China)

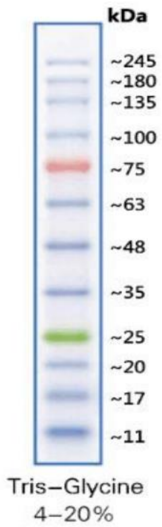

Fig1 L

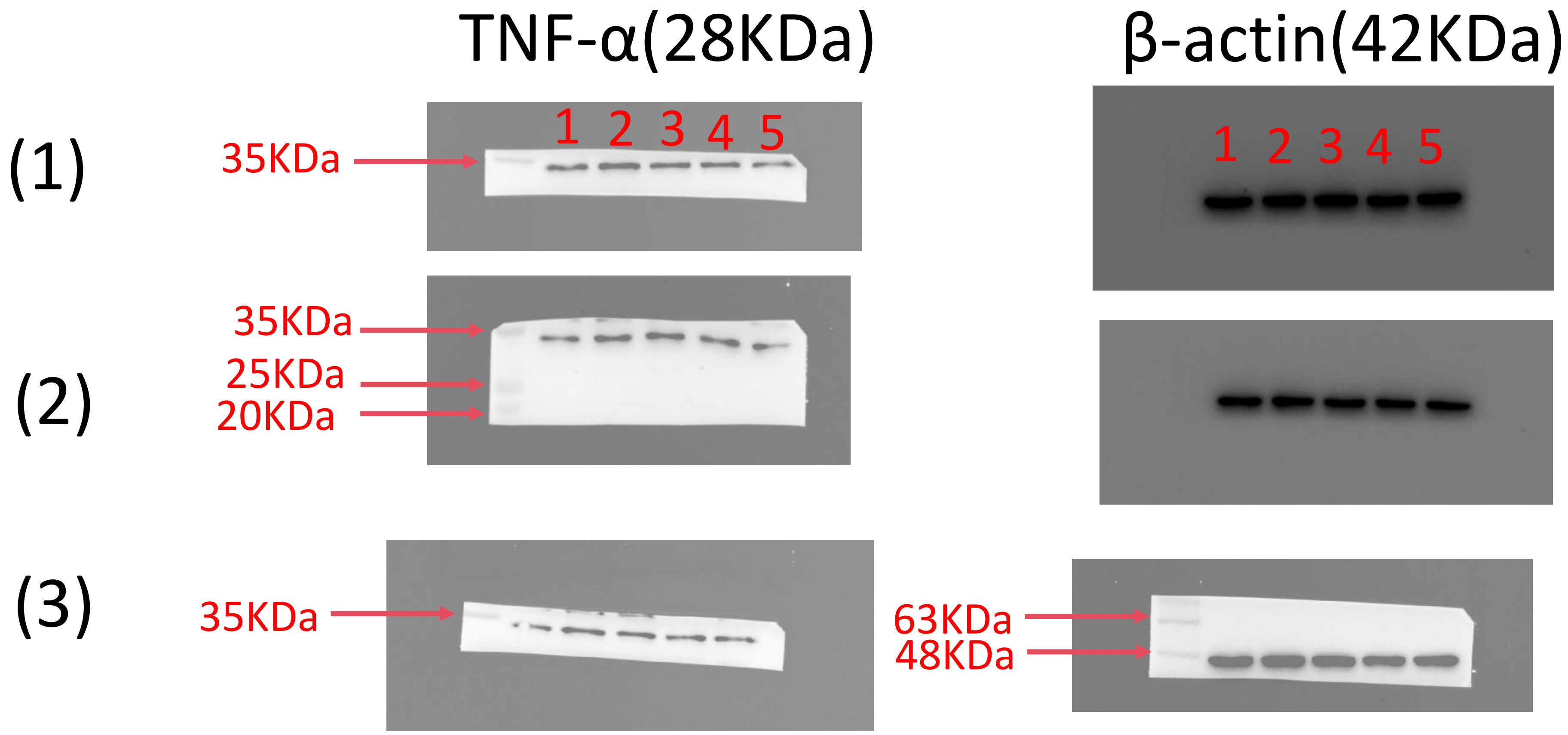

1: Control
2: LPS
3: LPS+Paroxetine (20mg/kg)
4: LPS+FMN(20mg/kg)
5: LPS+FMN(40mg/kg)

The protein band marker(PR1920) was purchased from the Solarbio (Beijing, China)

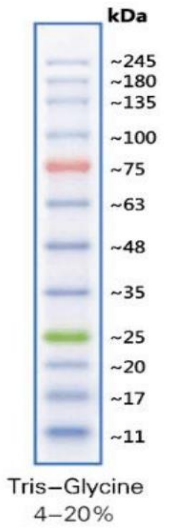

Fig1 L

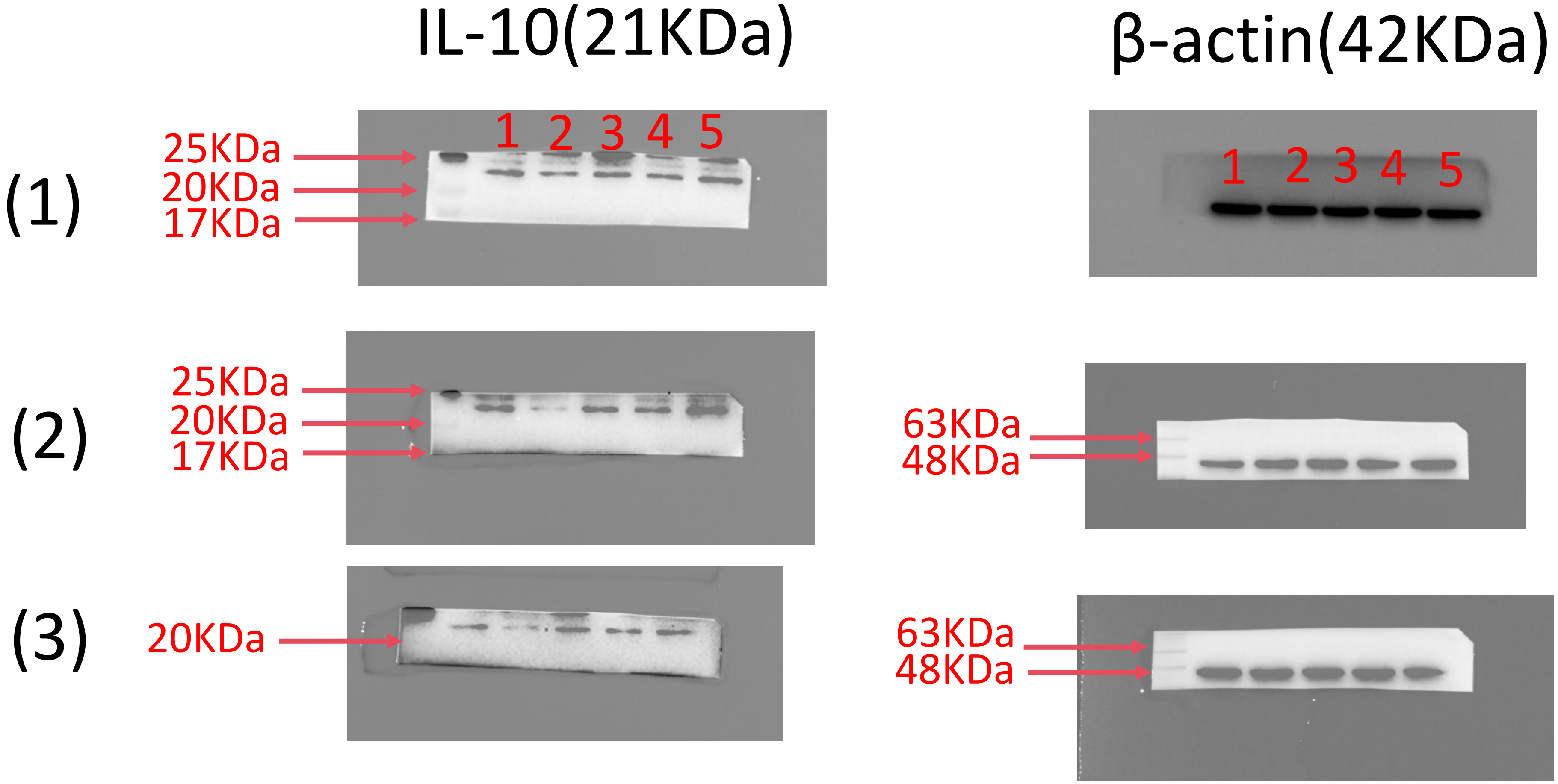

1: Control
2: LPS
3: LPS+Paroxetine (20mg/kg)
4: LPS+FMN(20mg/kg)
5: LPS+FMN(40mg/kg)

The protein band marker(PR1920) was purchased from the Solarbio (Beijing, China)

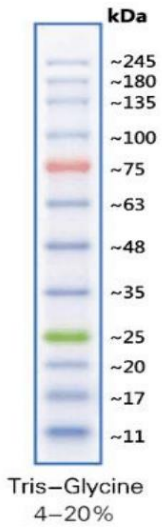

Fig2 E

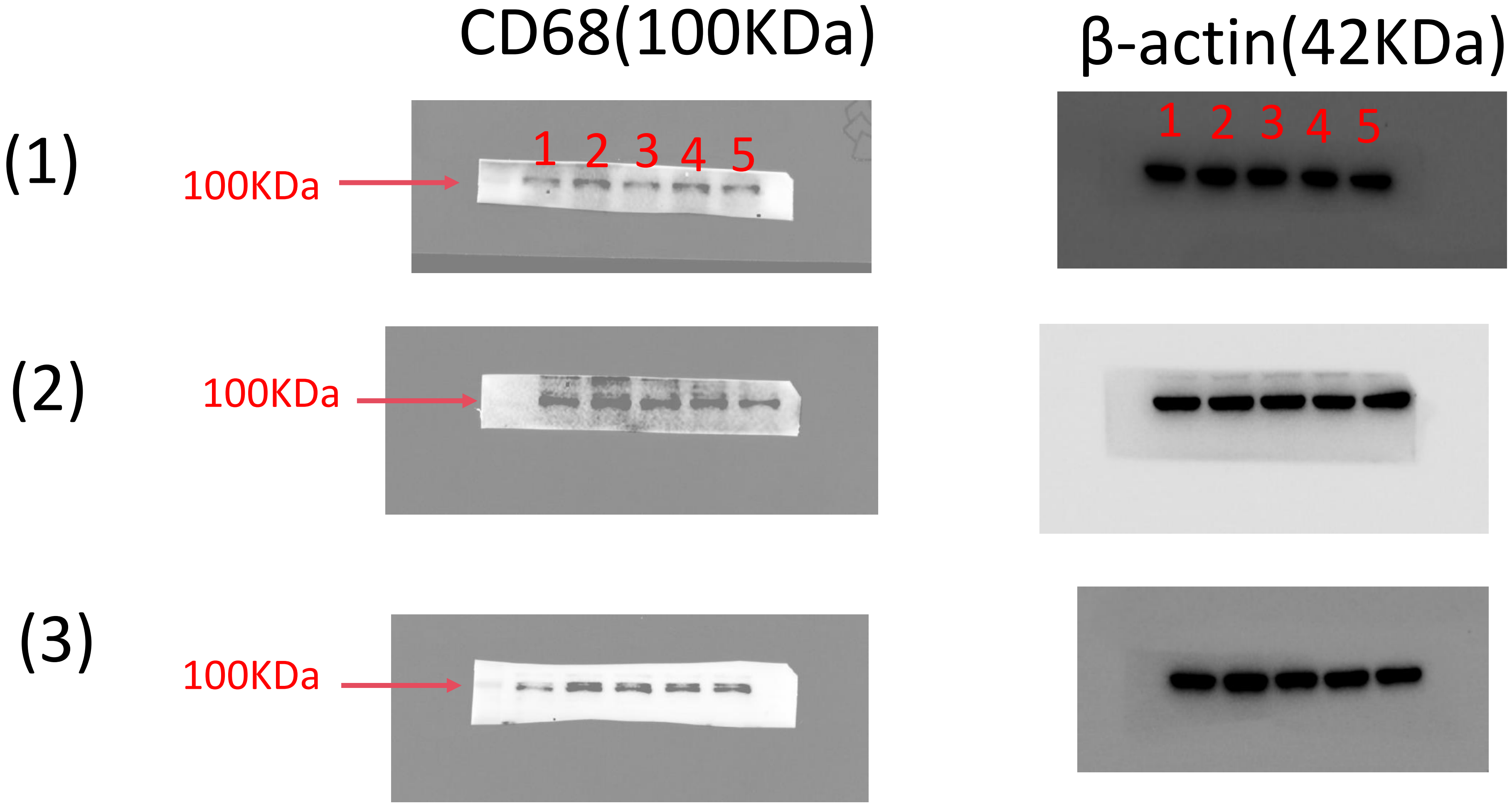

1: Control
2: LPS
3: LPS+Paroxetine (20mg/kg)
4: LPS+FMN(20mg/kg)
5: LPS+FMN(40mg/kg)

The protein band marker(PR1920) was purchased from the Solarbio (Beijing, China)

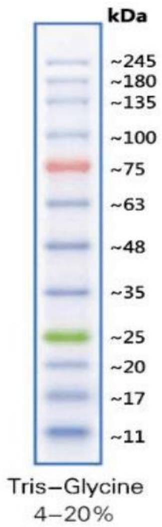

Fig2 E

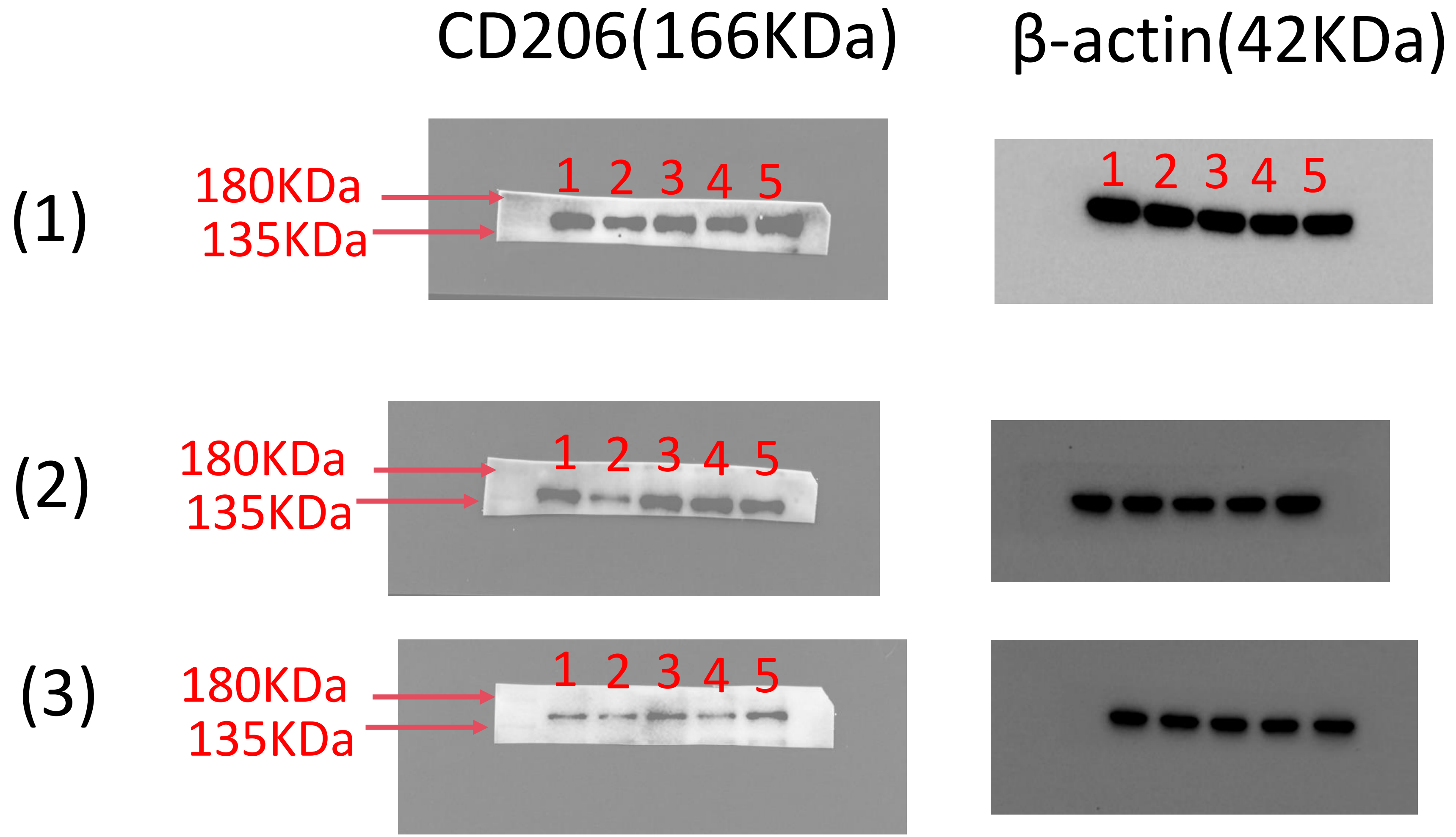

1: Control
2: LPS
3: LPS+Paroxetine (20mg/kg)
4: LPS+FMN(20mg/kg)
5: LPS+FMN(40mg/kg)

The protein band marker(PR1920) was purchased from the Solarbio (Beijing, China)

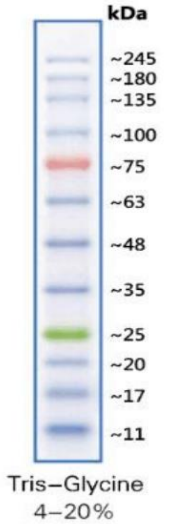

Fig2 L

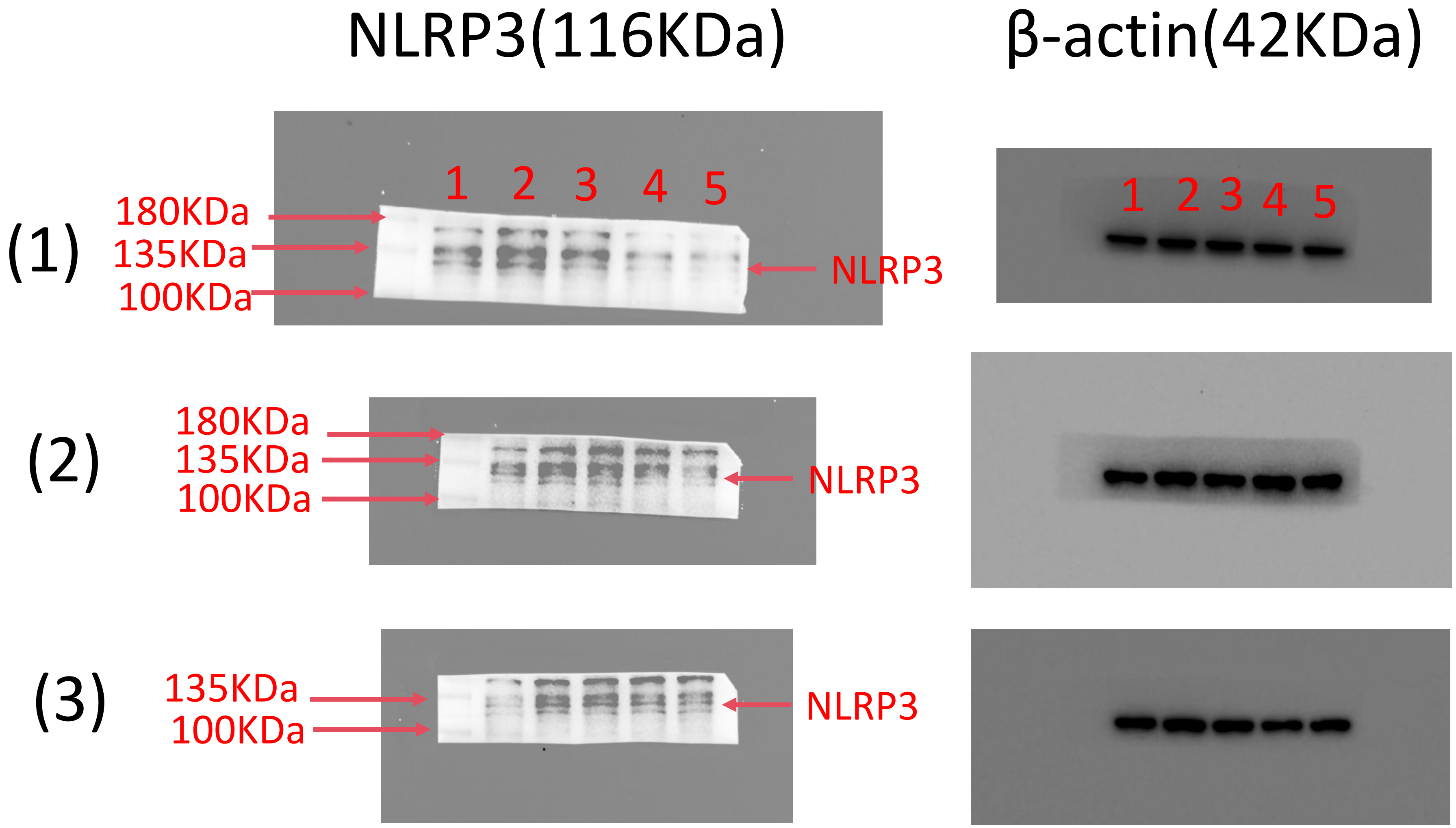

1: Control
2: LPS
3: LPS+Paroxetine (20mg/kg)
4: LPS+FMN(20mg/kg)
5: LPS+FMN(40mg/kg)

The protein band marker(PR1920) was purchased from the Solarbio (Beijing, China)

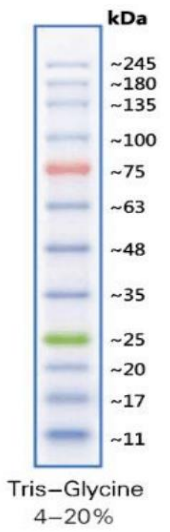

Fig2 L

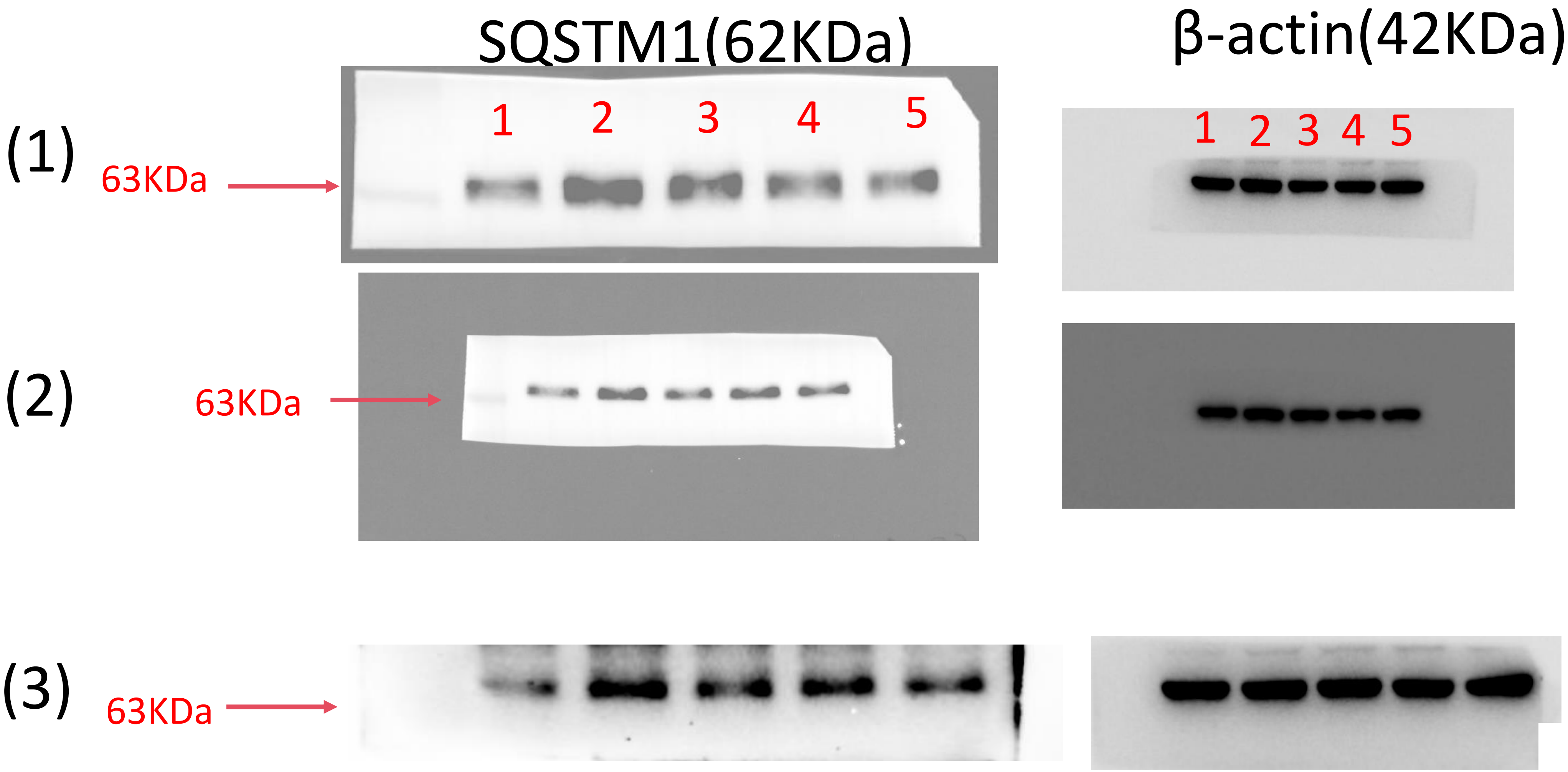

1: Control
2: LPS
3: LPS+Paroxetine (20mg/kg)
4: LPS+FMN(20mg/kg)
5: LPS+FMN(40mg/kg)

The protein band marker(PR1920) was purchased from the Solarbio (Beijing, China)

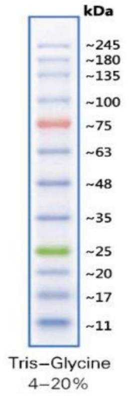

Fig2 L

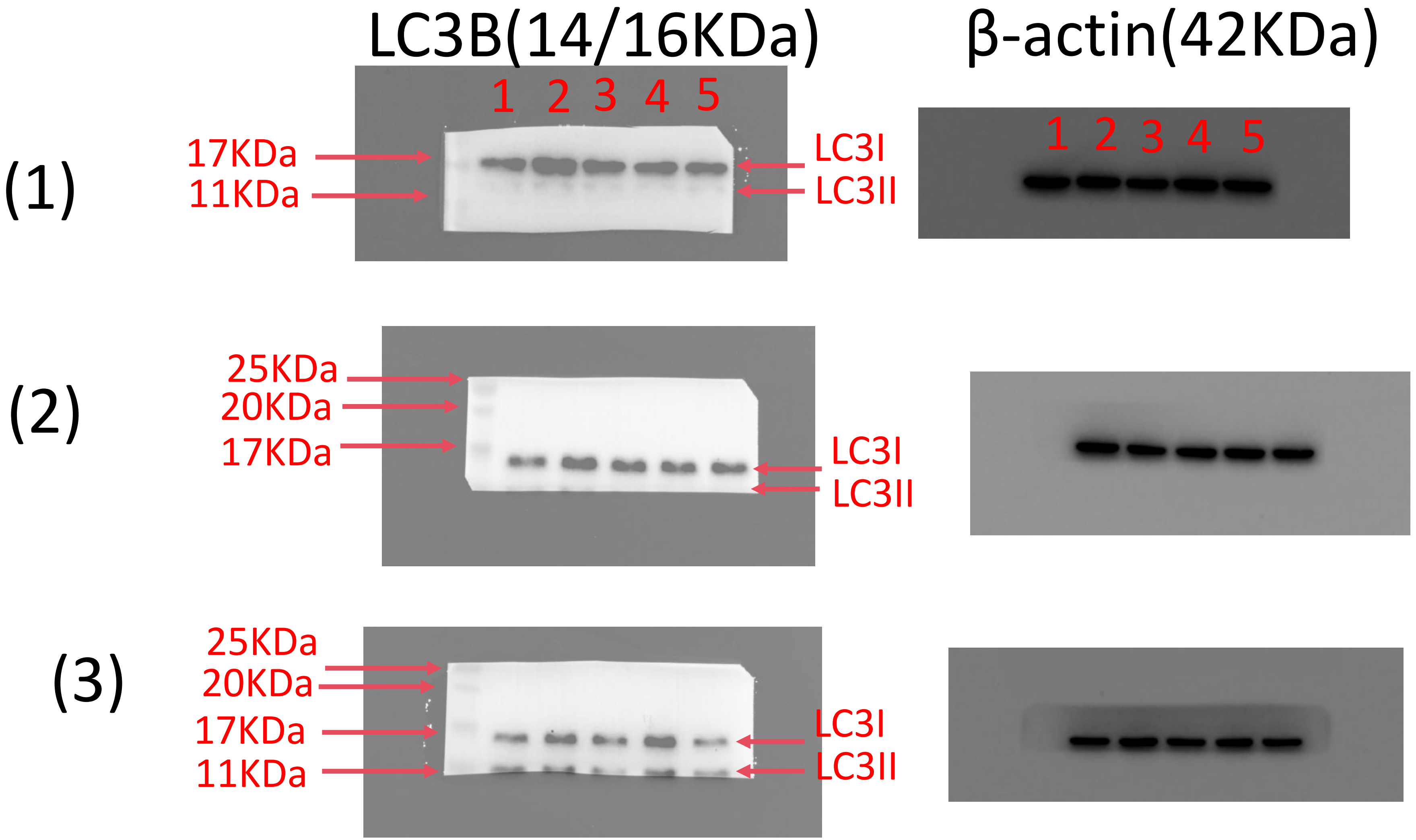

1: Control
2: LPS
3: LPS+Paroxetine (20mg/kg)
4: LPS+FMN(20mg/kg)
5: LPS+FMN(40mg/kg)

The protein band marker(PR1920) was purchased from the Solarbio (Beijing, China)

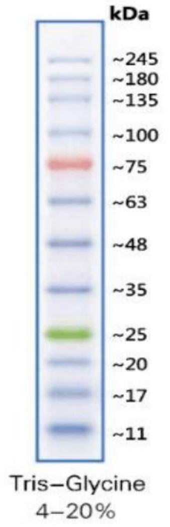

Fig3 B

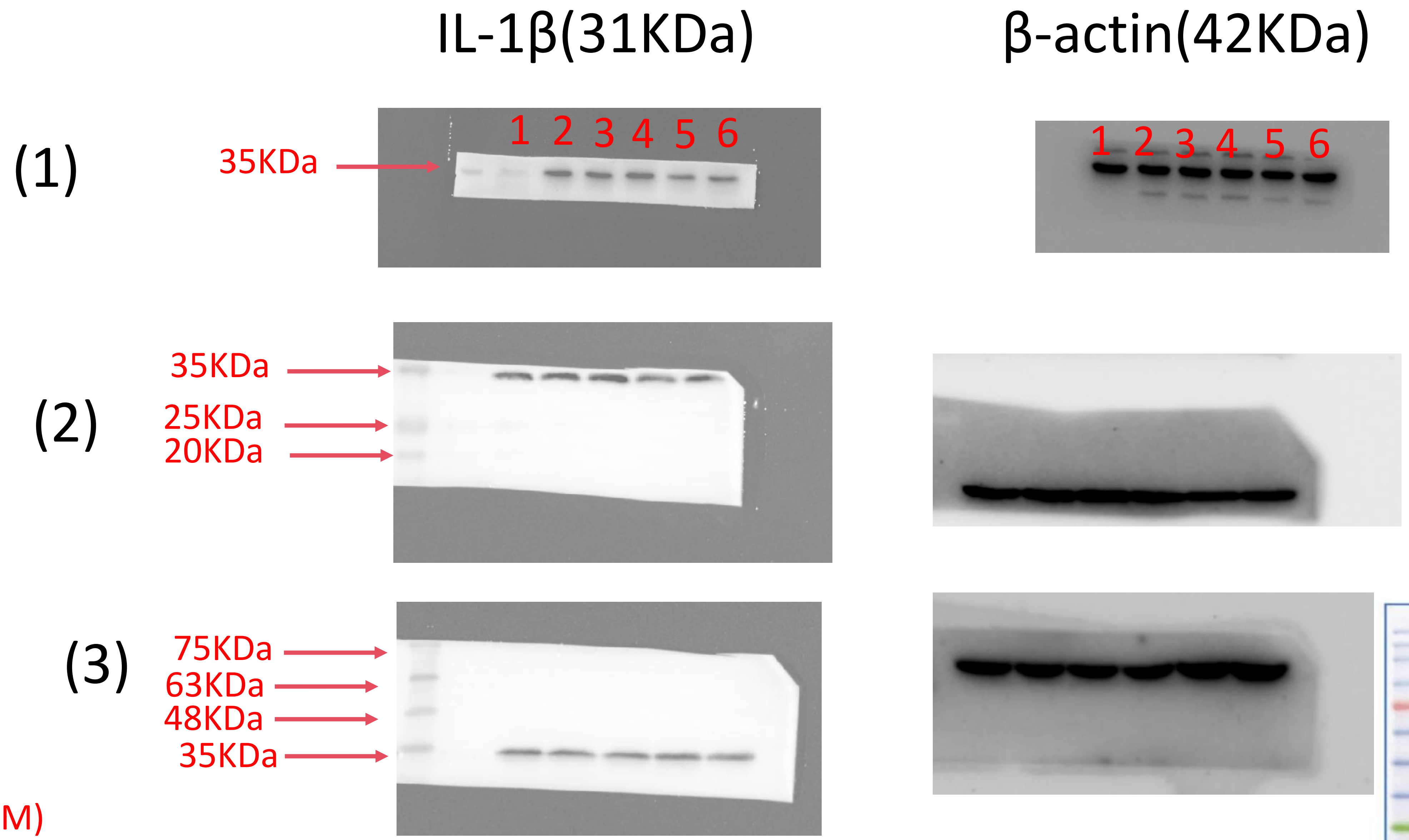

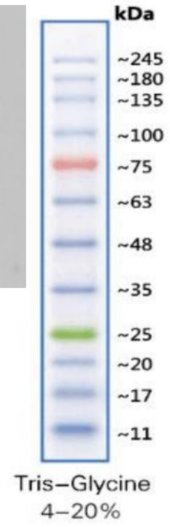

1: Control
2:LPS
3:LPS+FMN(5 μM)
4:LPS+FMN(10 μM)
5:LPS+FMN(20 μM)
6:LPS+FMN(30 μM)

The protein band marker(PR1920) was purchased from the Solarbio (Beijing, China)

Fig3 B

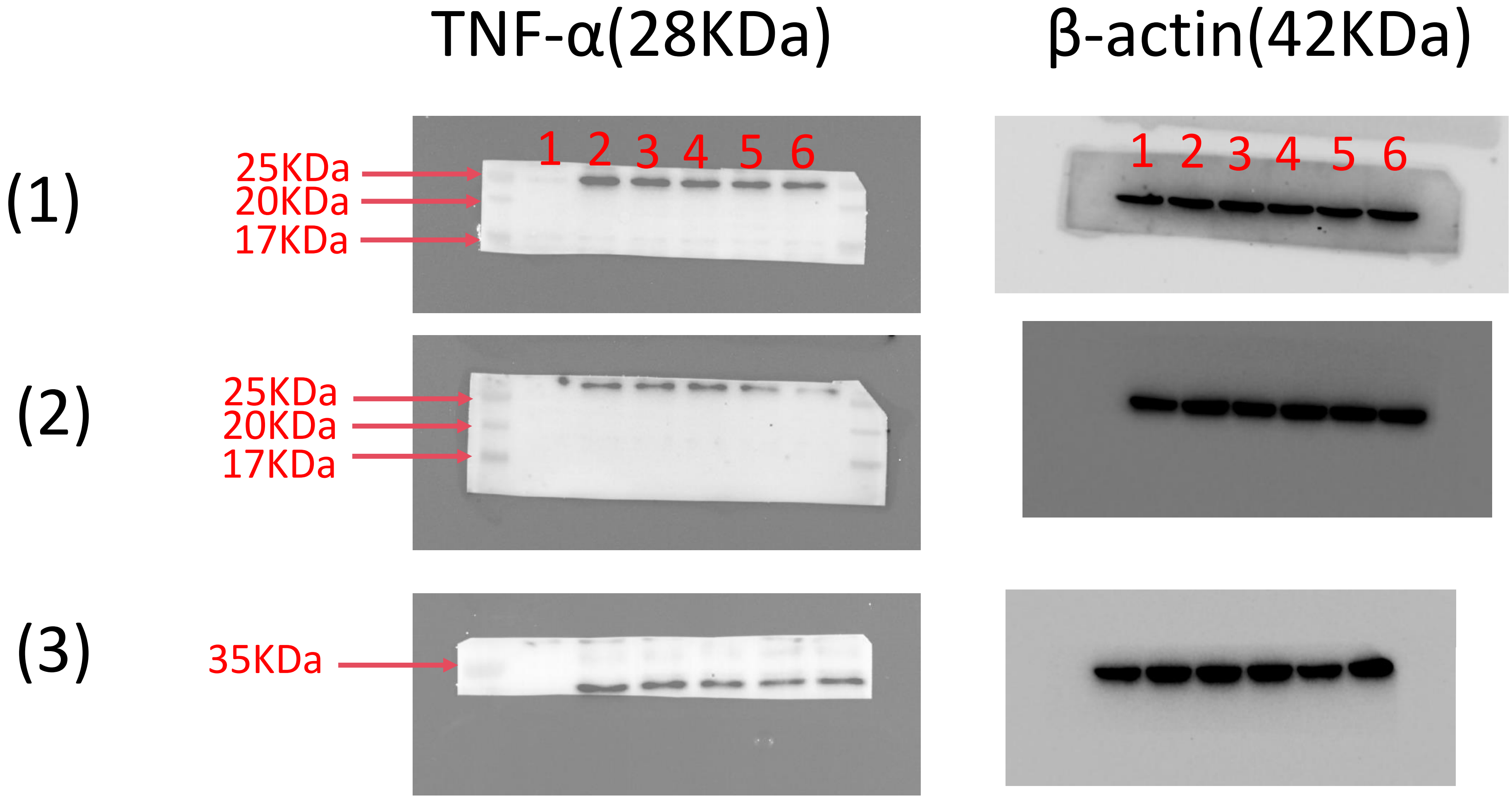

1: Control
2:LPS
3:LPS+FMN(5 μM)
4:LPS+FMN(10 μM)
5:LPS+FMN(20 μM)
6:LPS+FMN(30 μM)

The protein band marker(PR1920) was purchased from the Solarbio (Beijing, China)

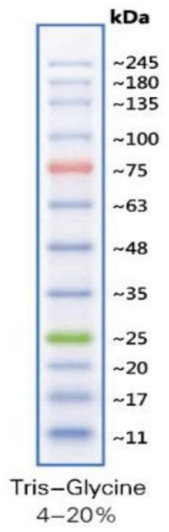

Fig3 B

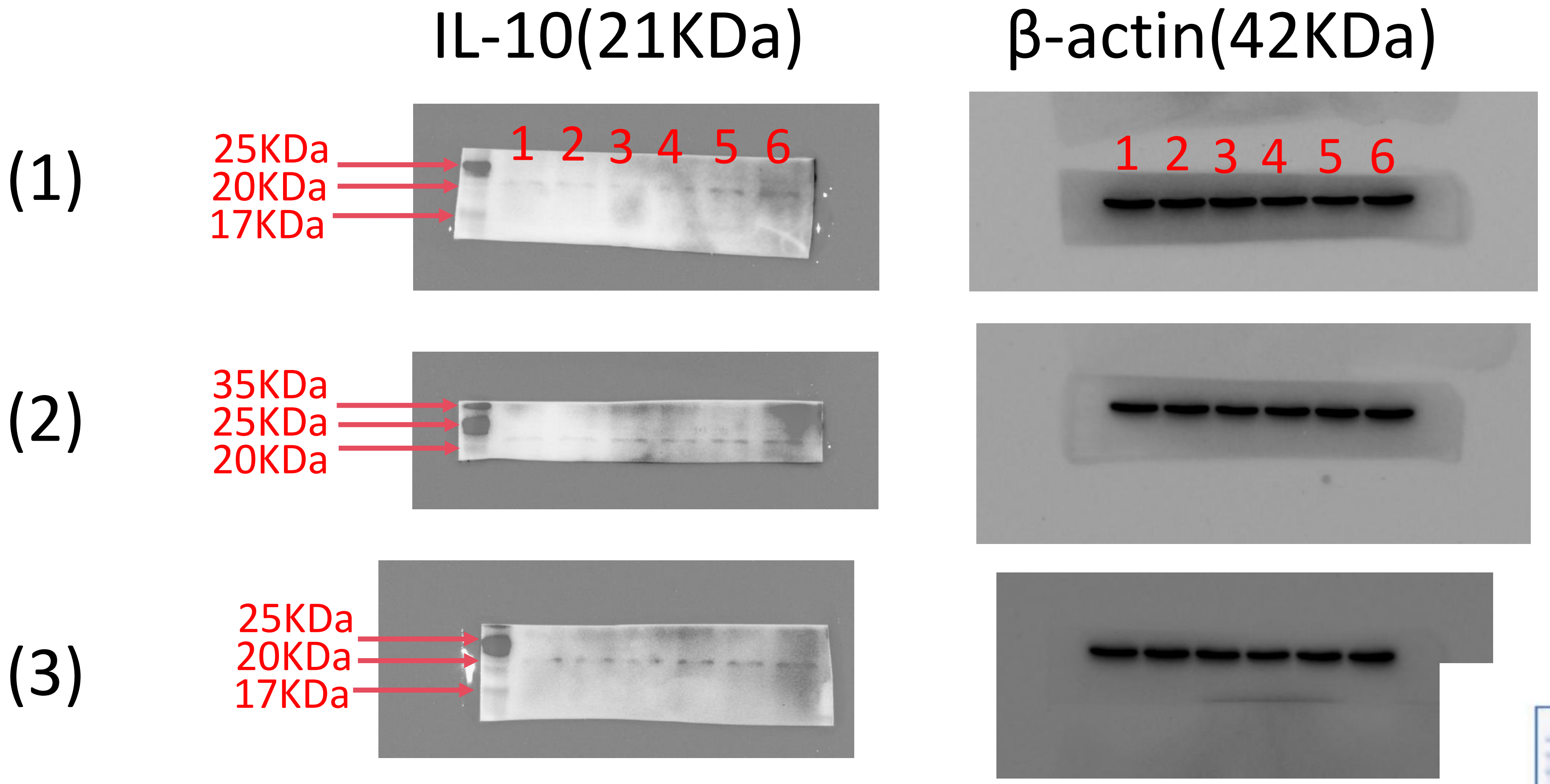

1: Control
2:LPS
3:LPS+FMN(5 μM)
4:LPS+FMN(10 μM)
5:LPS+FMN(20 μM)
6:LPS+FMN(30 μM)

The protein band marker(PR1920) was purchased from the Solarbio (Beijing, China)

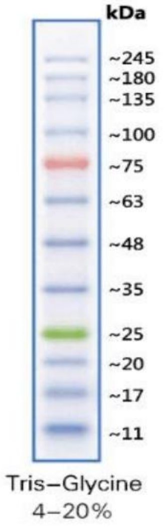

Fig3 D

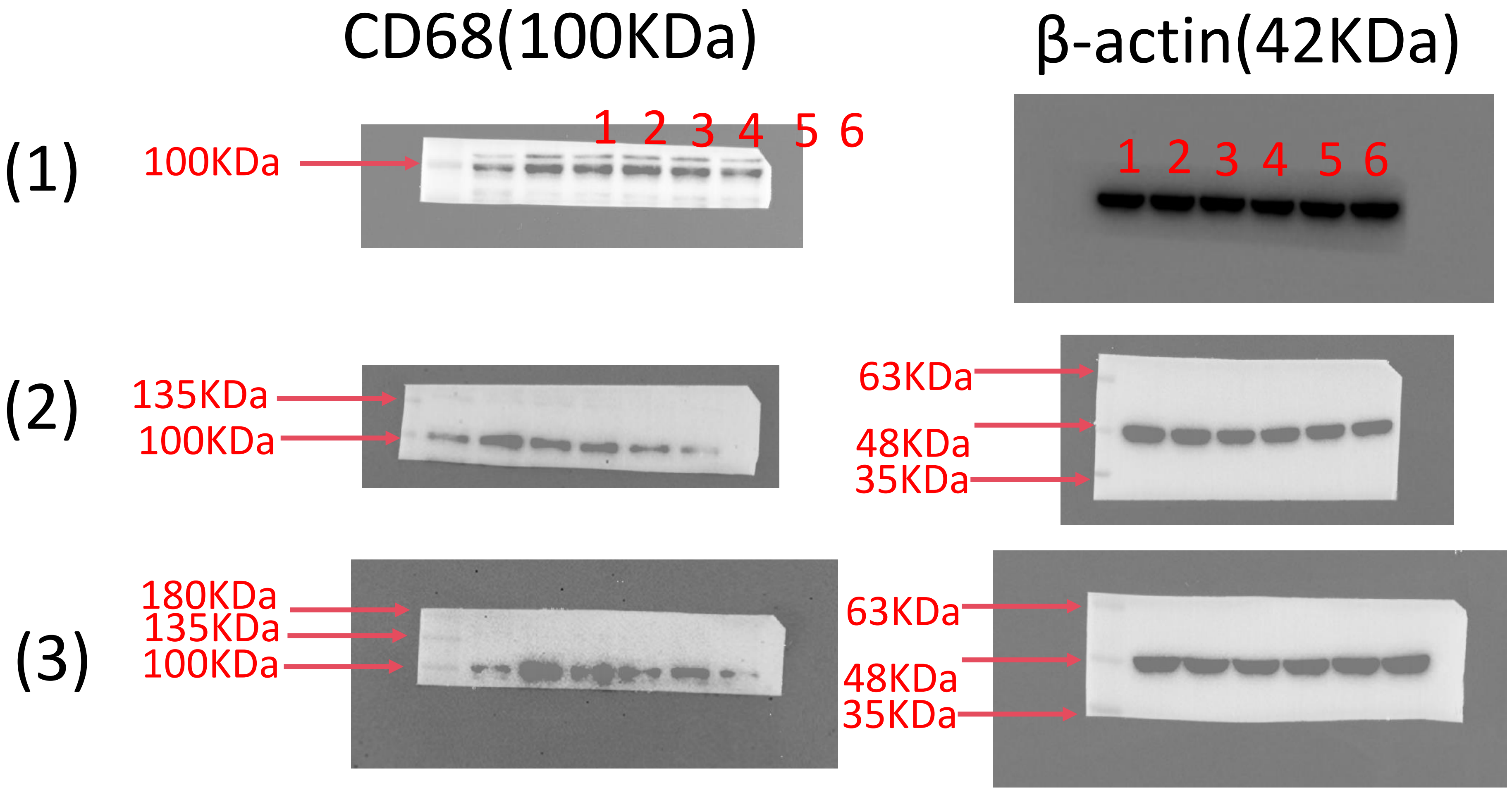

1: Control
2:LPS
3:LPS+FMN(5 μM)
4:LPS+FMN(10 μM)
5:LPS+FMN(20 μM)
6:LPS+FMN(30 μM)

The protein band marker(PR1920) was purchased from the Solarbio (Beijing, China)

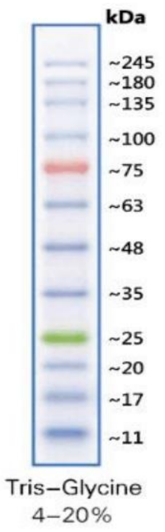

Fig3 D

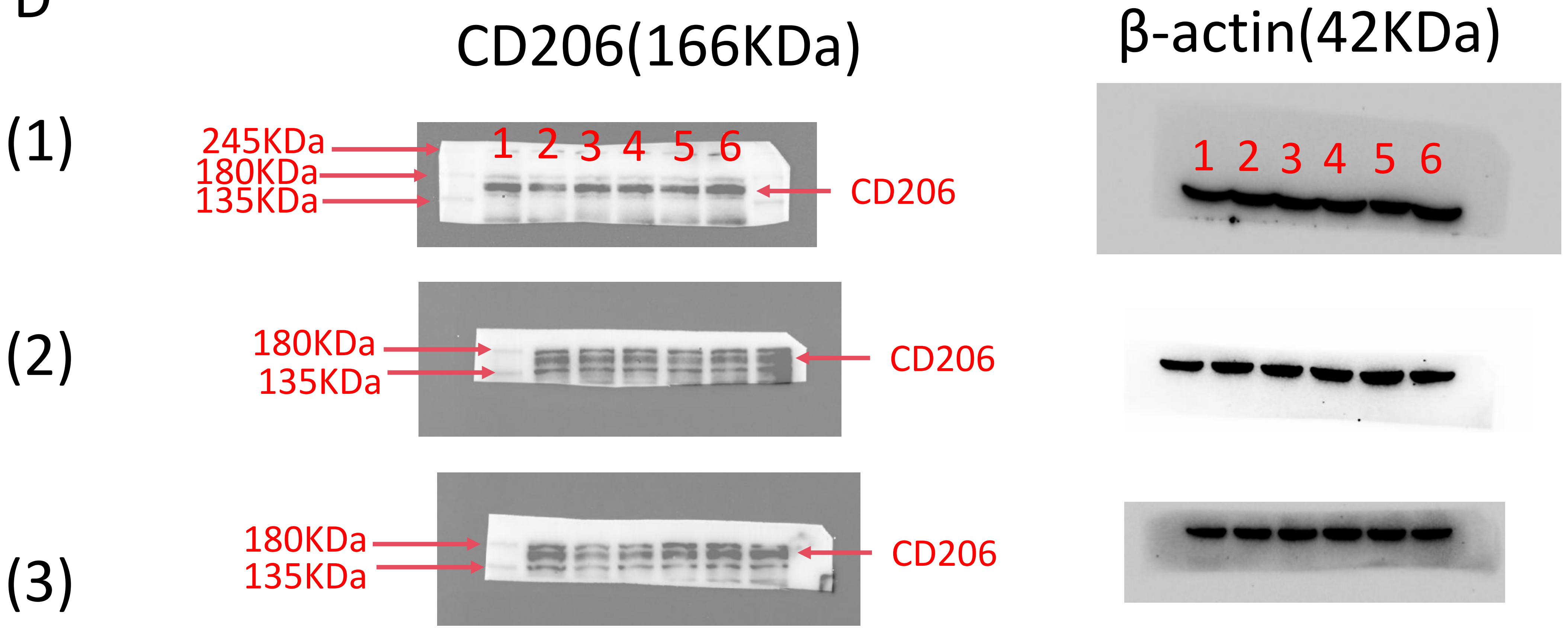

1: Control
2:LPS
3:LPS+FMN(5 μM)
4:LPS+FMN(10 μM)
5:LPS+FMN(20 μM)
6:LPS+FMN(30 μM)

The protein band marker(PR1920) was purchased from the Solarbio (Beijing, China)

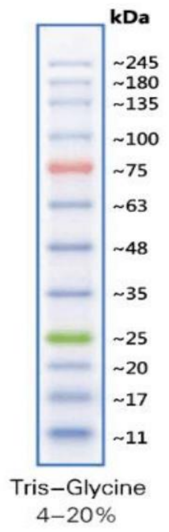

Fig3 D

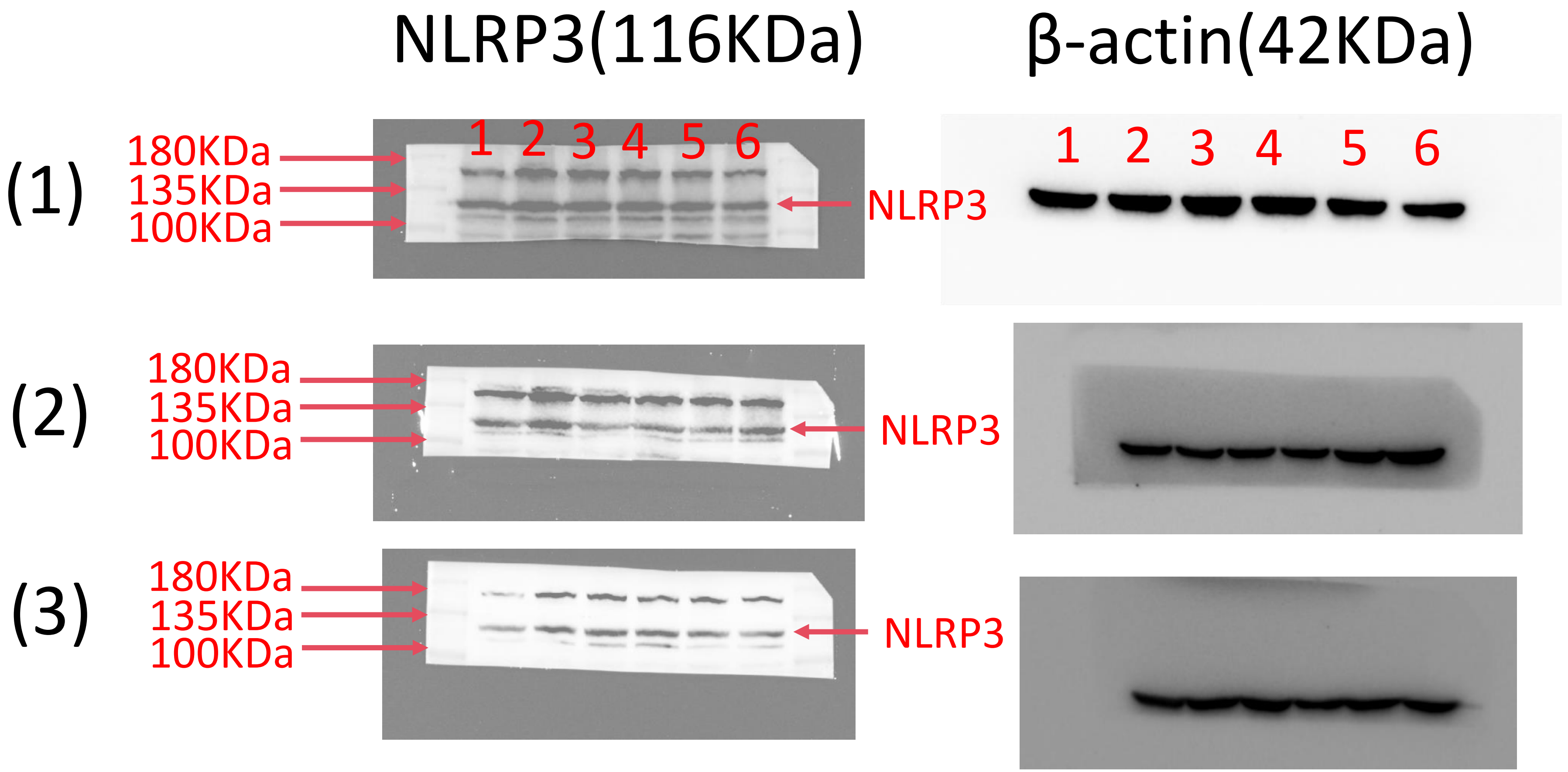

1: Control
2:LPS
3:LPS+FMN(5 μM)
4:LPS+FMN(10 μM)
5:LPS+FMN(20 μM)
6:LPS+FMN(30 μM)

The protein band marker(PR1920) was purchased from the Solarbio (Beijing, China)

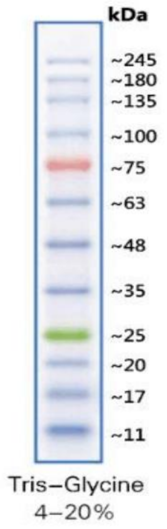

Fig3 D

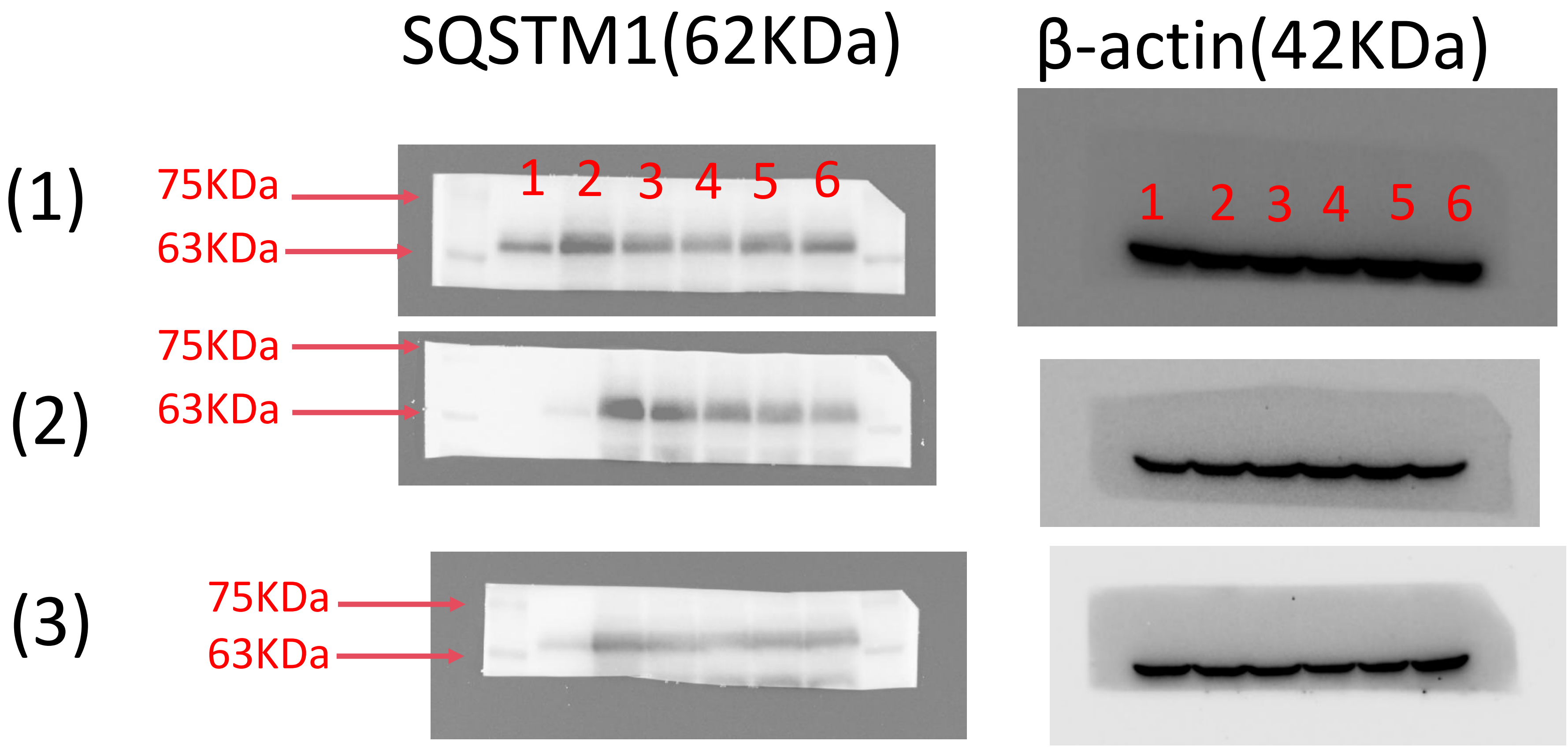

1: Control
2:LPS
3:LPS+FMN(5 μM)
4:LPS+FMN(10 μM)
5:LPS+FMN(20 μM)
6:LPS+FMN(30 μM)

The protein band marker(PR1920) was purchased from the Solarbio (Beijing, China)

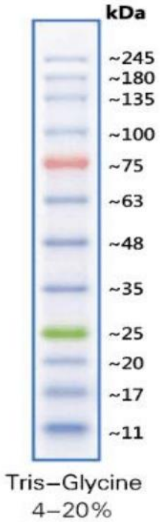

Fig3 D

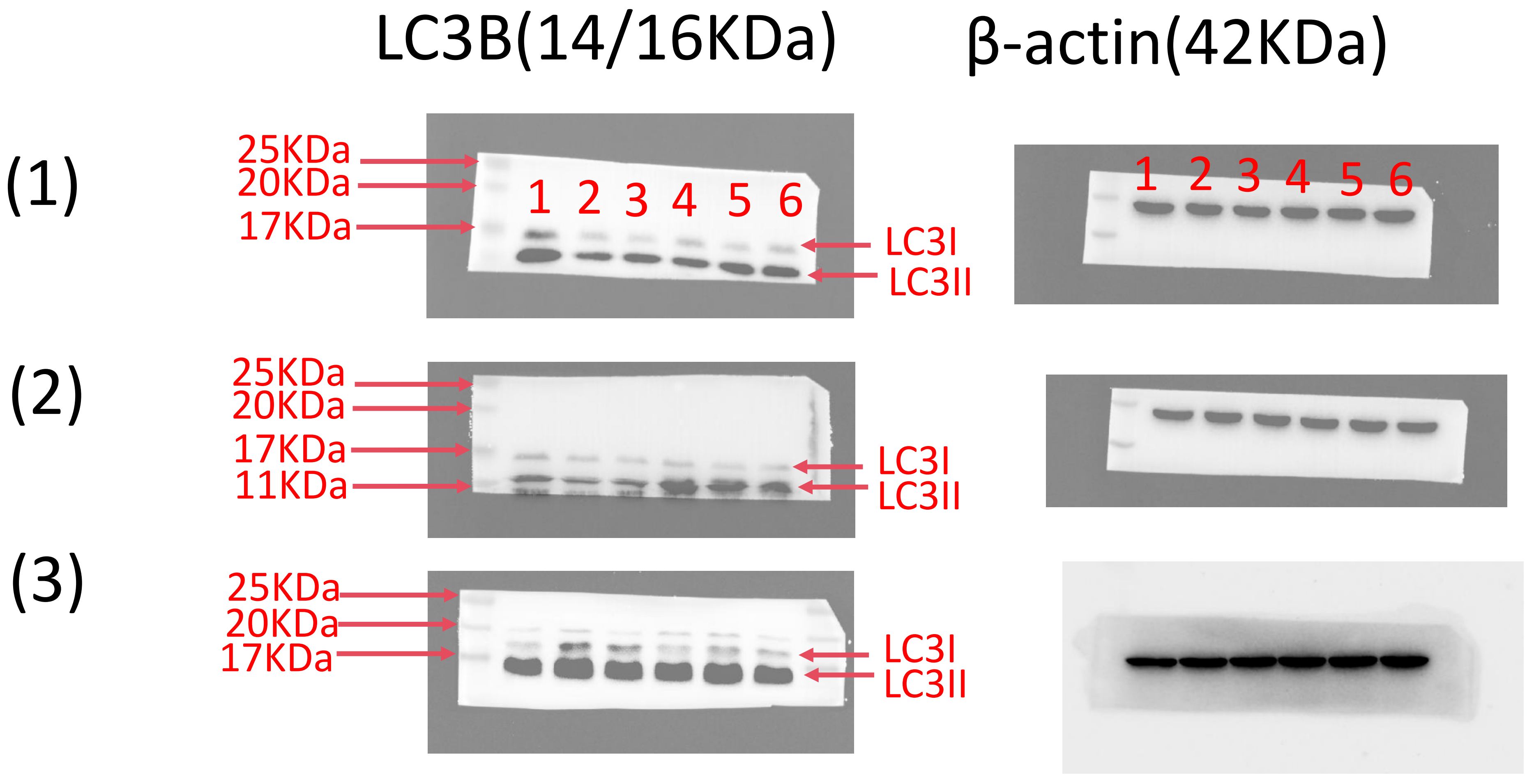

1: Control
2:LPS
3:LPS+FMN(5 μM)
4:LPS+FMN(10 μM)
5:LPS+FMN(20 μM)
6:LPS+FMN(30 μM)

The protein band marker(PR1920) was purchased from the Solarbio (Beijing, China)

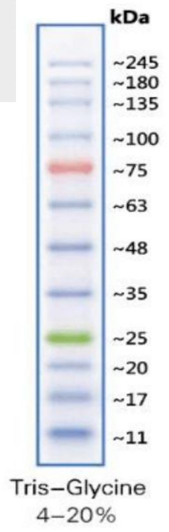

Fig4 A

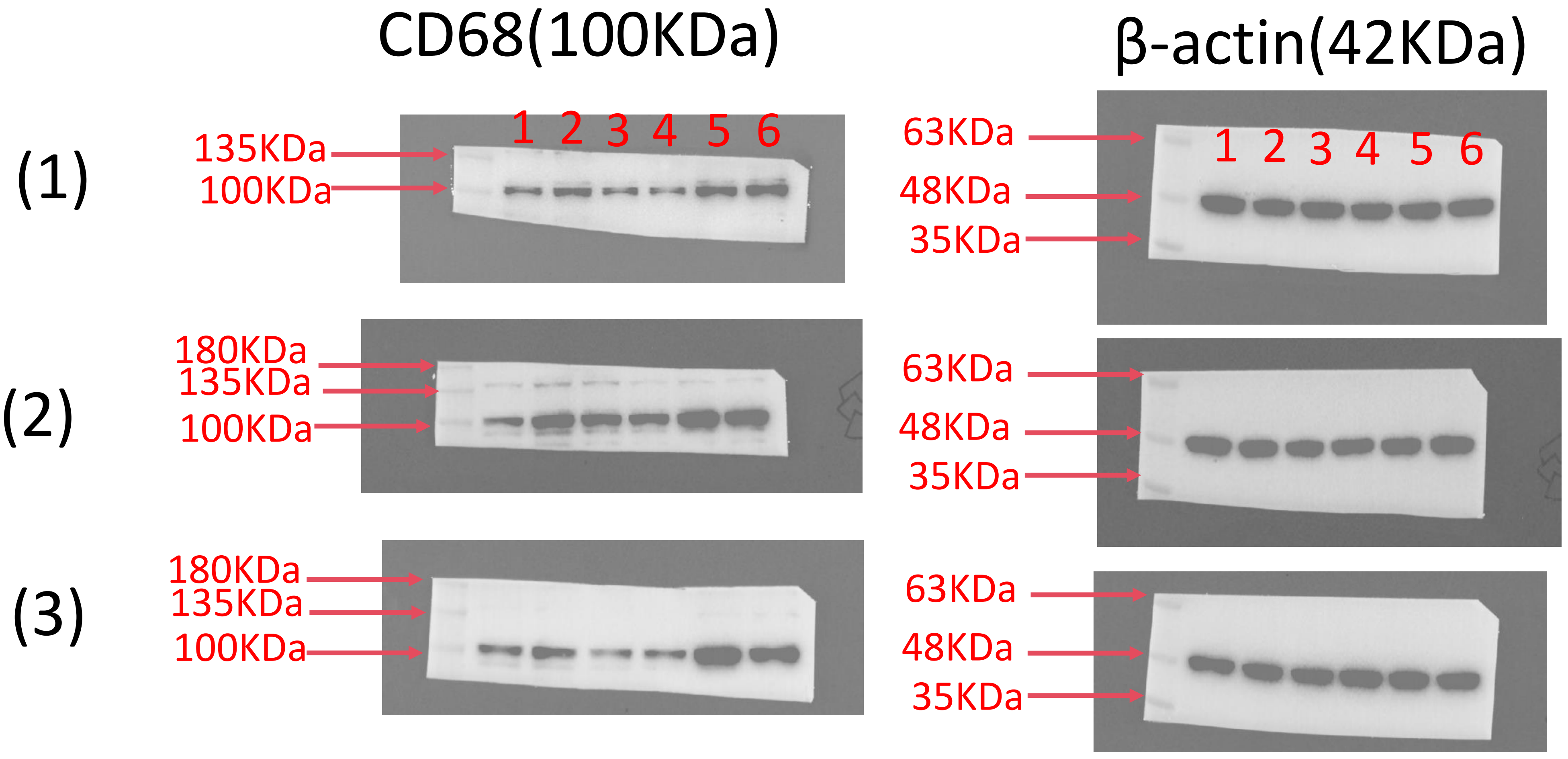

1: Control
2:LPS
3:LPS+Mino(20 μM)
4:LPS+FMN(30 μM)
5:LPS+FMN(30 μM)+CQ(20 μM)
6:LPS+CQ(20 μM)

The protein band marker(PR1920) was purchased from the Solarbio (Beijing, China)

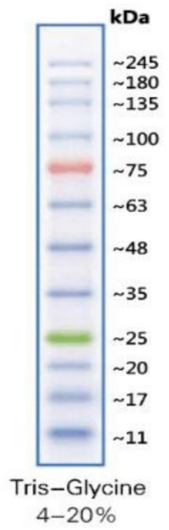

Fig4 A

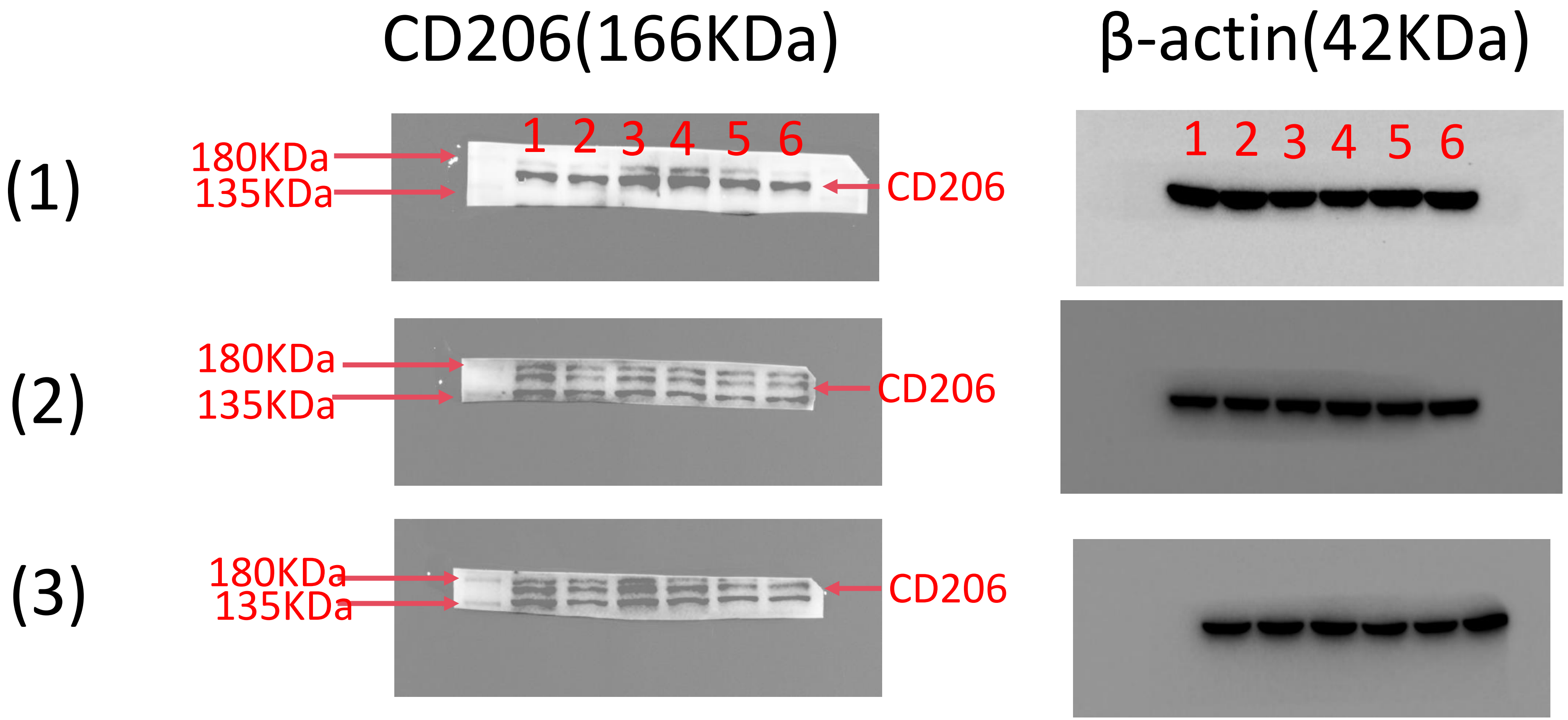

1: Control
2:LPS
3:LPS+Mino(20 μM)
4:LPS+FMN(30 μM)
5:LPS+FMN(30 μM)+CQ(20 μM)
6:LPS+CQ(20 μM)

The protein band marker(PR1920) was purchased from the Solarbio (Beijing, China)

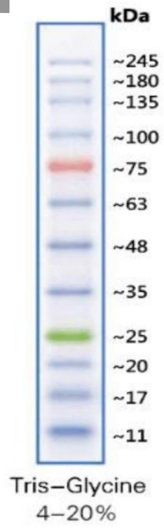

Fig4 A

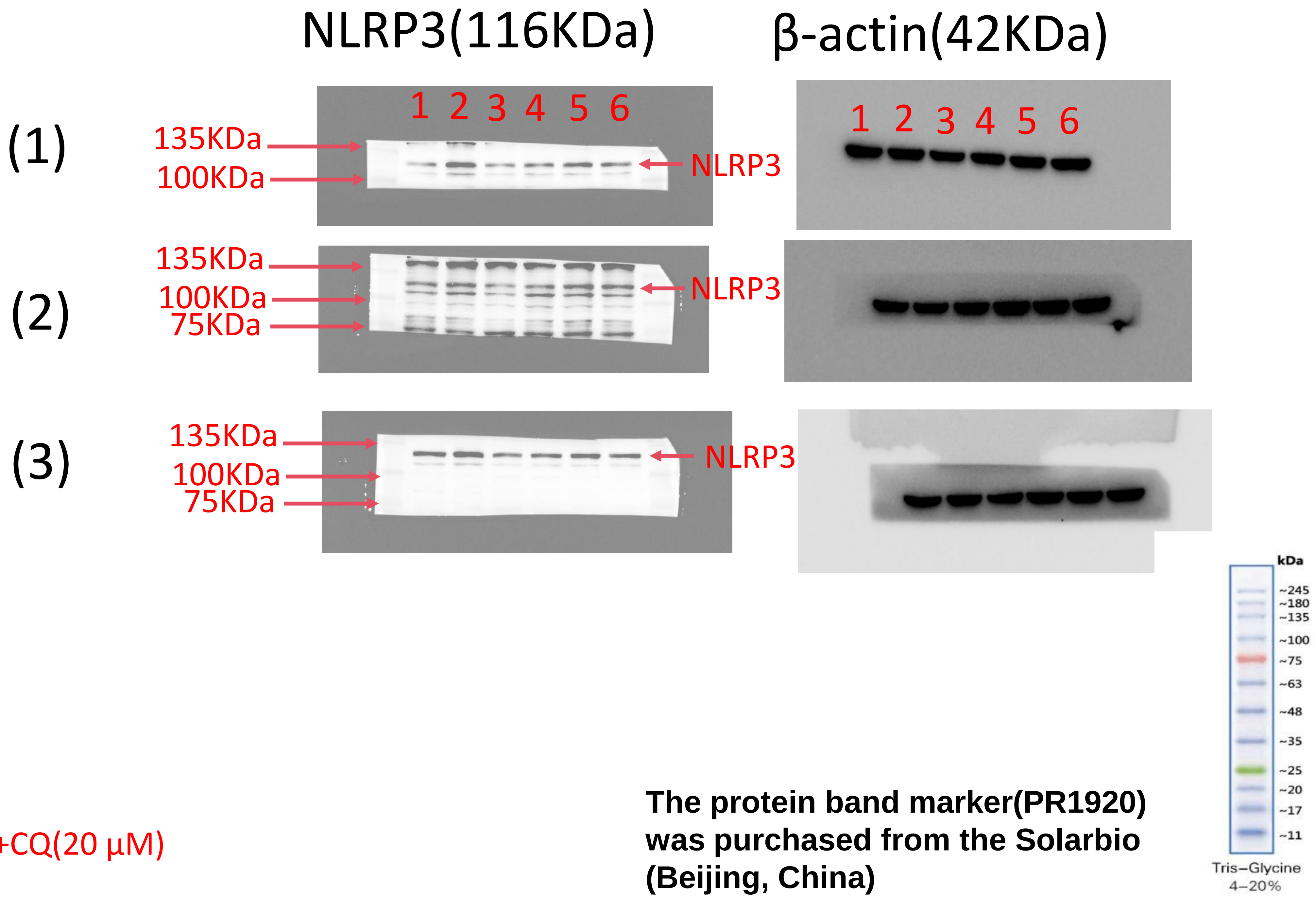

1: Control
2:LPS
3:LPS+Mino(20 μM)
4:LPS+FMN(30 μM)
5:LPS+FMN(30 μM)+CQ(20 μM)
6:LPS+CQ(20 μM)

The protein band marker(PR1920) was purchased from the Solarbio (Beijing, China)

Fig4 C

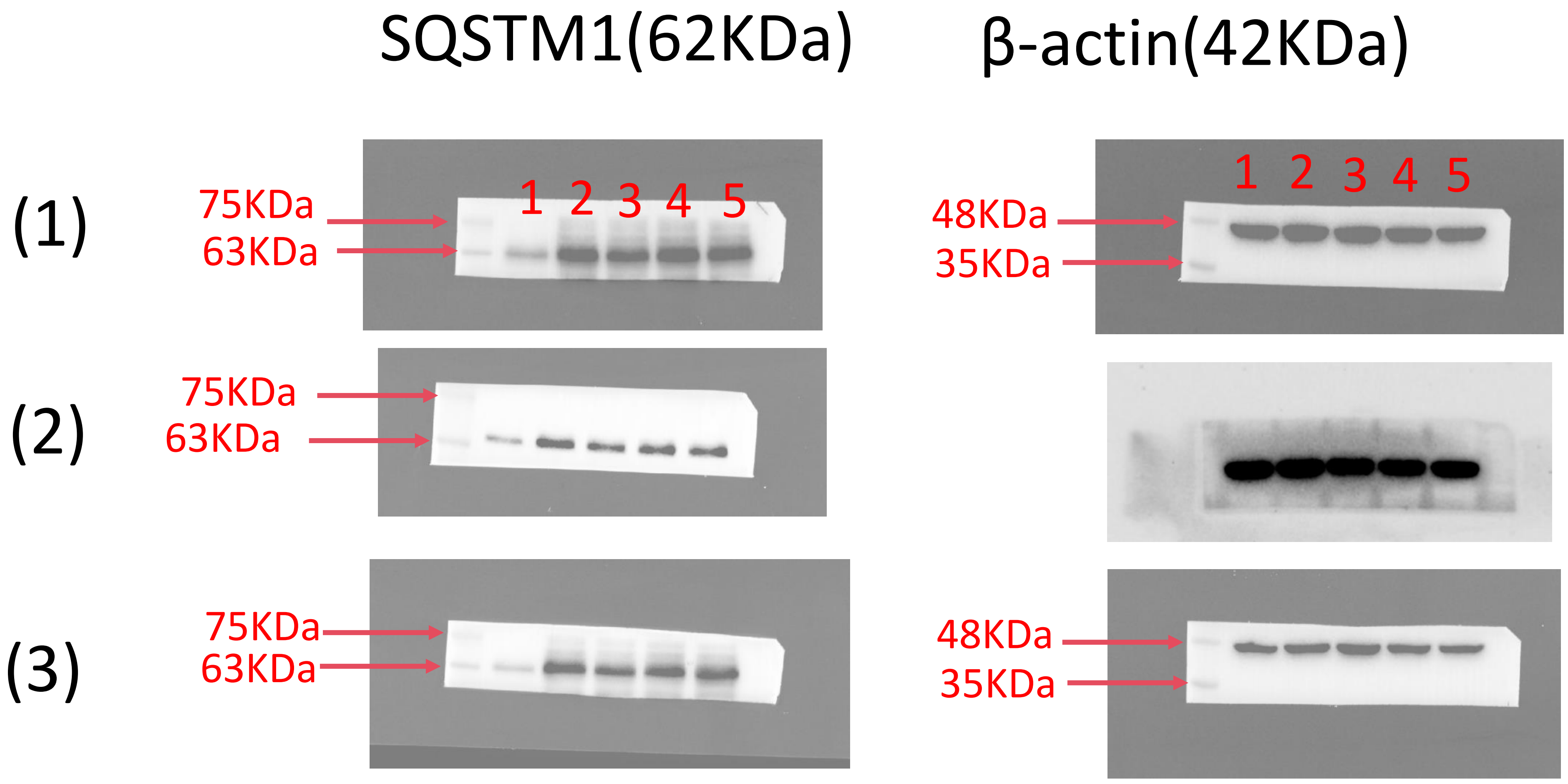

1: Control
2:LPS
3:LPS+FMN(30 μM)
4:LPS+FMN(30 μM)+CQ(20 μM)
5:LPS+CQ(20 μM)

The protein band marker(PR1920) was purchased from the Solarbio (Beijing, China)

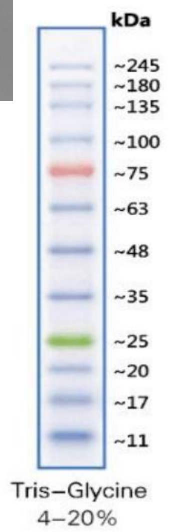

Fig4 C

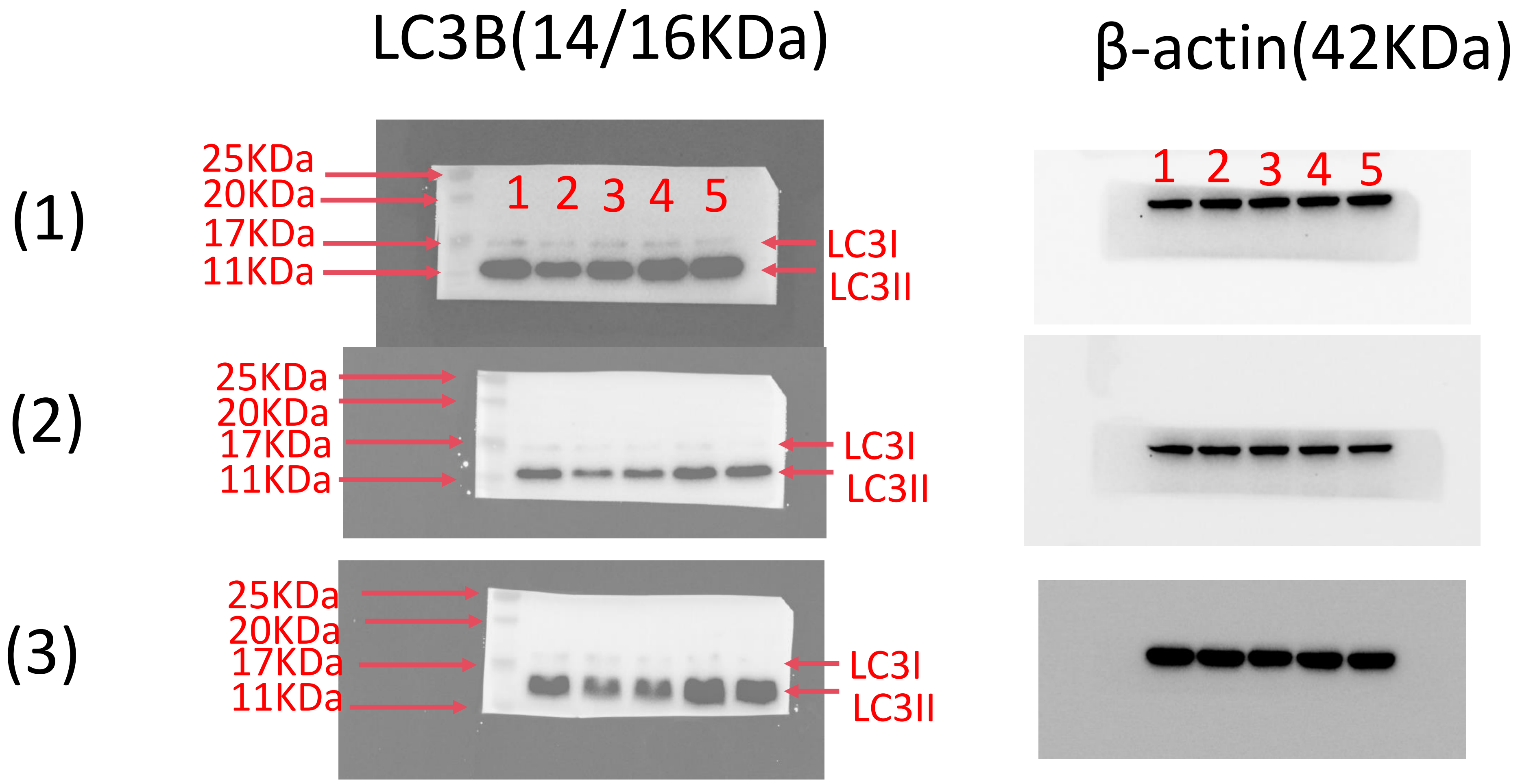

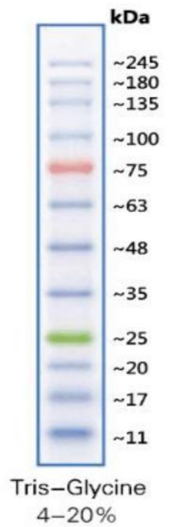

1: Control

2:LPS

3:LPS+FMN(30 μM)

4:LPS+FMN(30 μM)+CQ(20 μM)

5:LPS+CQ(20 μM)

The protein band marker(PR1920) was purchased from the Solarbio (Beijing, China)

Fig5 E

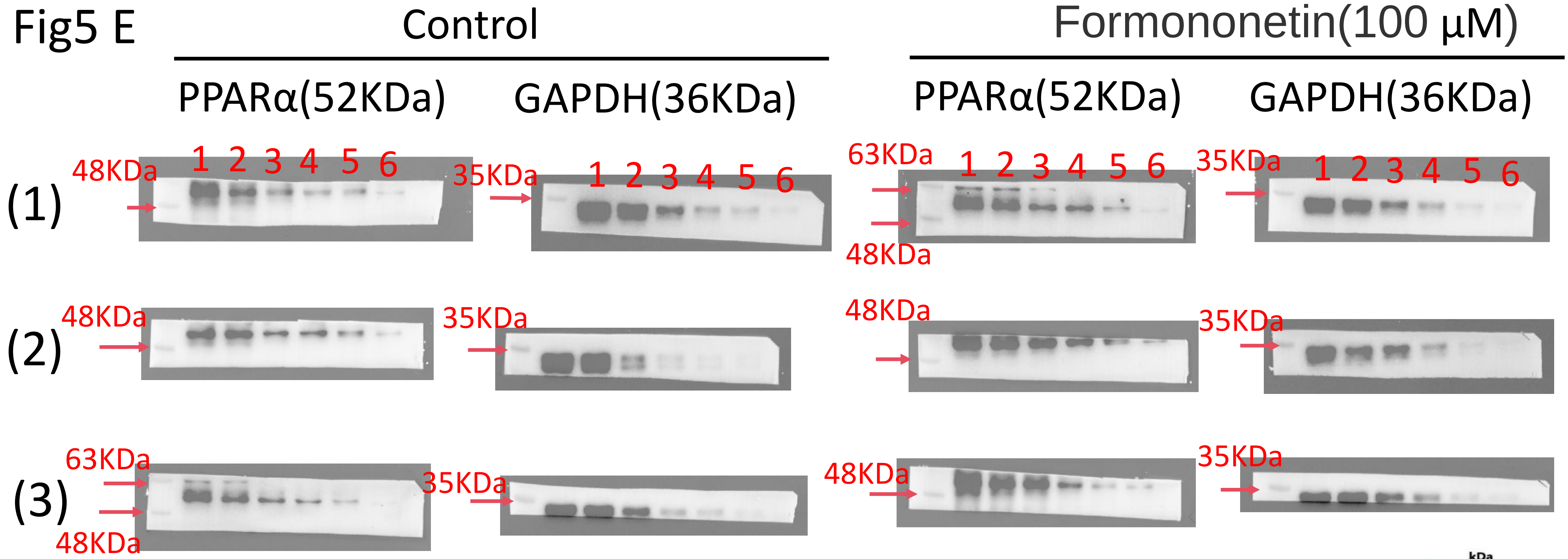

1: 45℃
2: 48℃
3: 51℃
4: 54℃
5: 57 ℃
6: 60 ℃

The protein band marker(PR1920) was purchased from the Solarbio (Beijing, China)

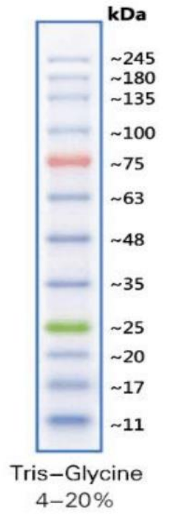

Fig5 F

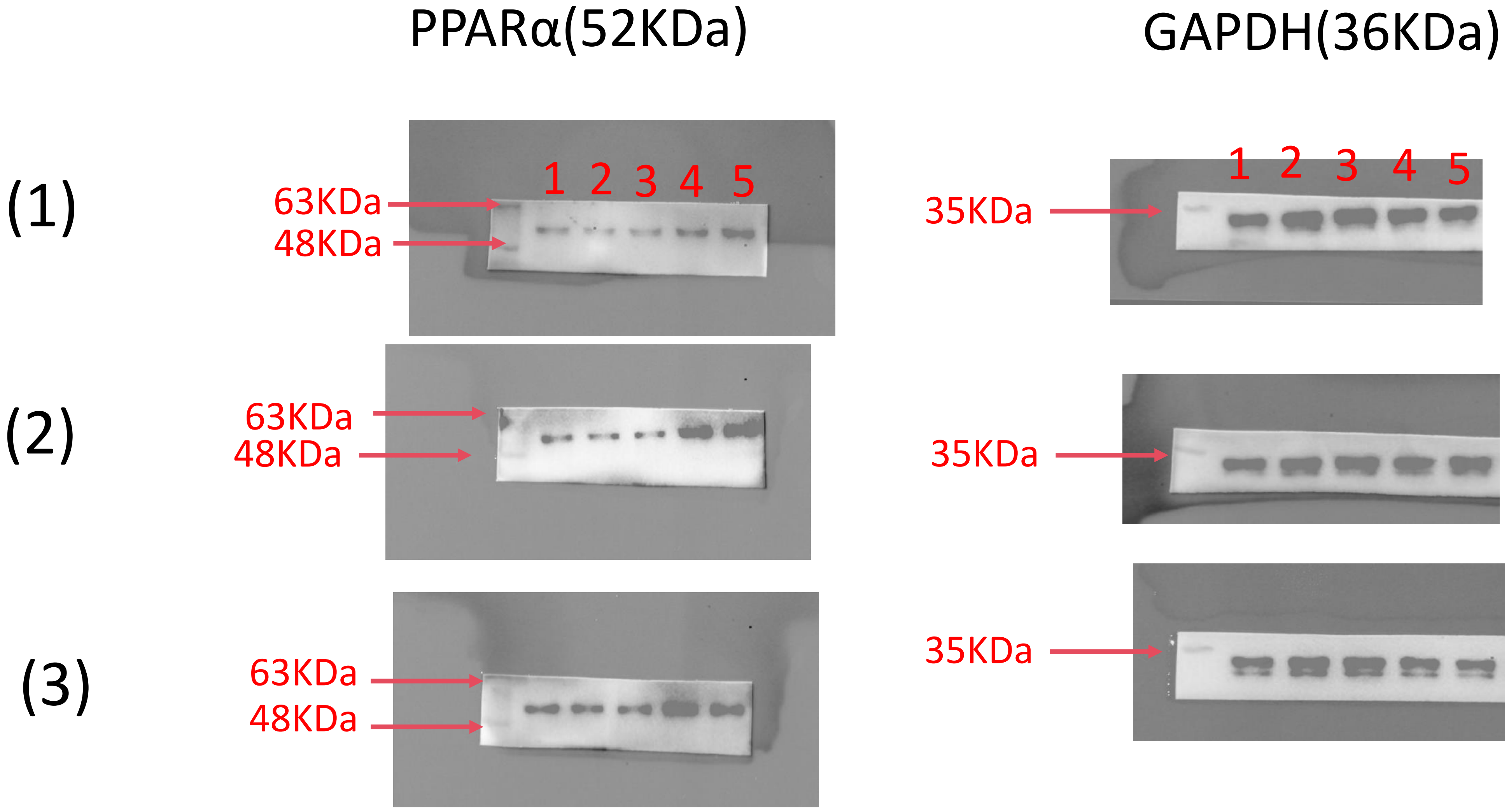

1: DMSO
2: FMN(25 μM)
3: FMN(50 μM)
4: FMN(100 μM)
5: FMN(200 μM)

The protein band marker(PR1920) was purchased from the Solarbio (Beijing, China)

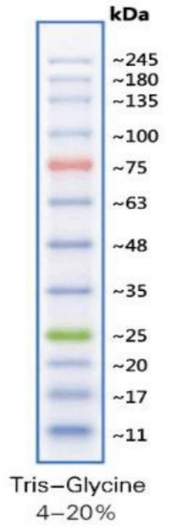

Fig5 G

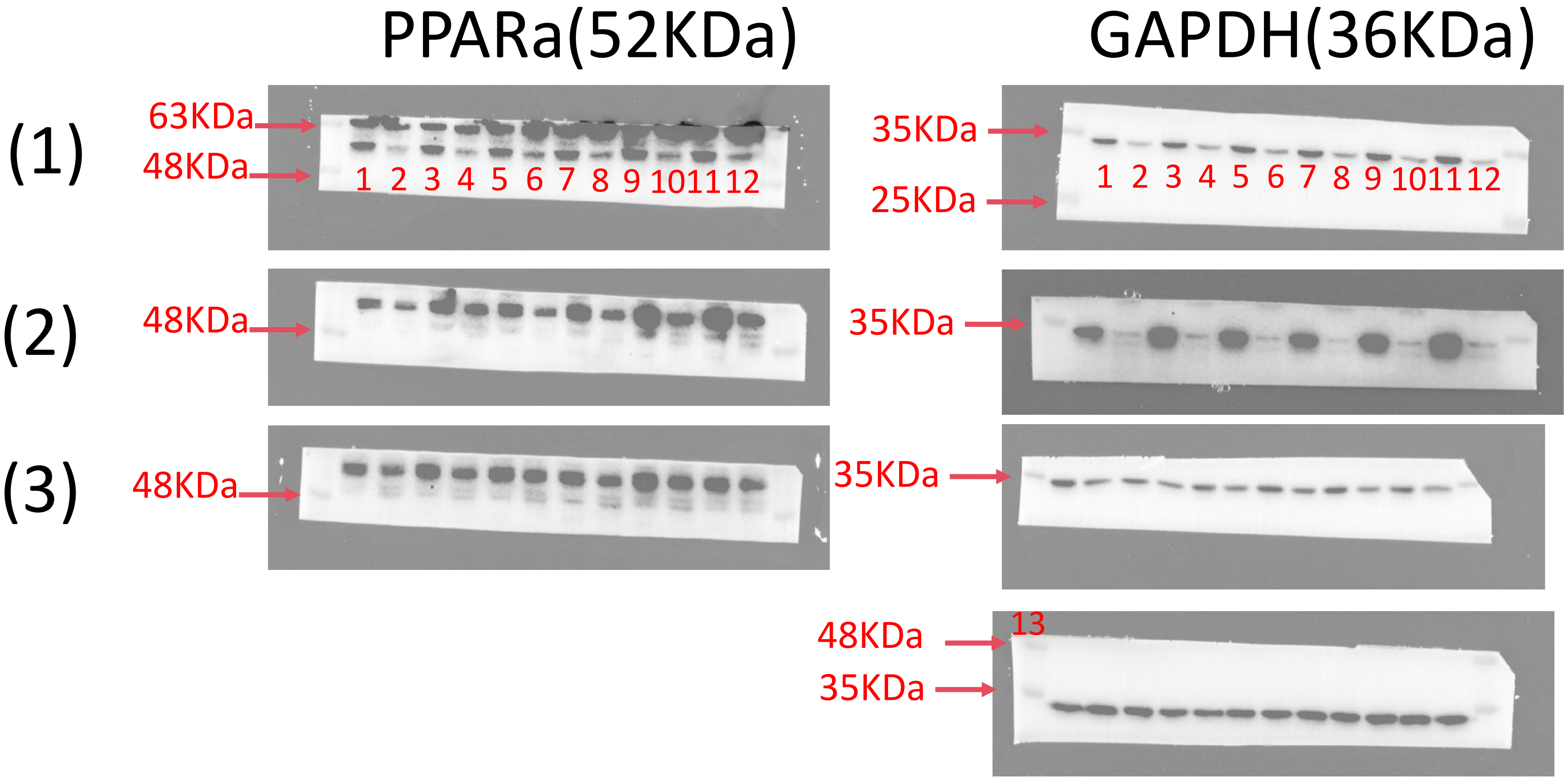

1: FMN 0uM+TNC 2: FMN 0uM+ pronase (0.01 mg/mL)
3: FMN 25uM+TNC 4: FMN 25uM+ pronase (0.01 mg/mL)
5: FMN 50uM+TNC 6: FMN 50uM+ pronase (0.01 mg/mL)
7: FMN 100uM+TNC 8: FMN 100uM+ pronase (0.01 mg/mL)
9: FMN 200uM+TNC 10: FMN 200uM+ pronase (0.01 mg/mL)
11: FMN 400uM+TNC 12: FMN 400uM+ pronase (0.01 mg/mL)
13:Not involved in the enzymatic hydrolysis process loading control

The protein band marker(PR1920) was purchased from the Solarbio (Beijing, China)

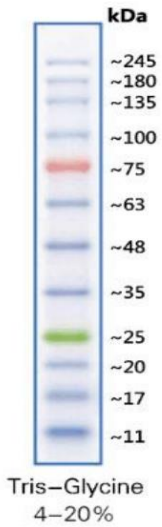

Fig6 A

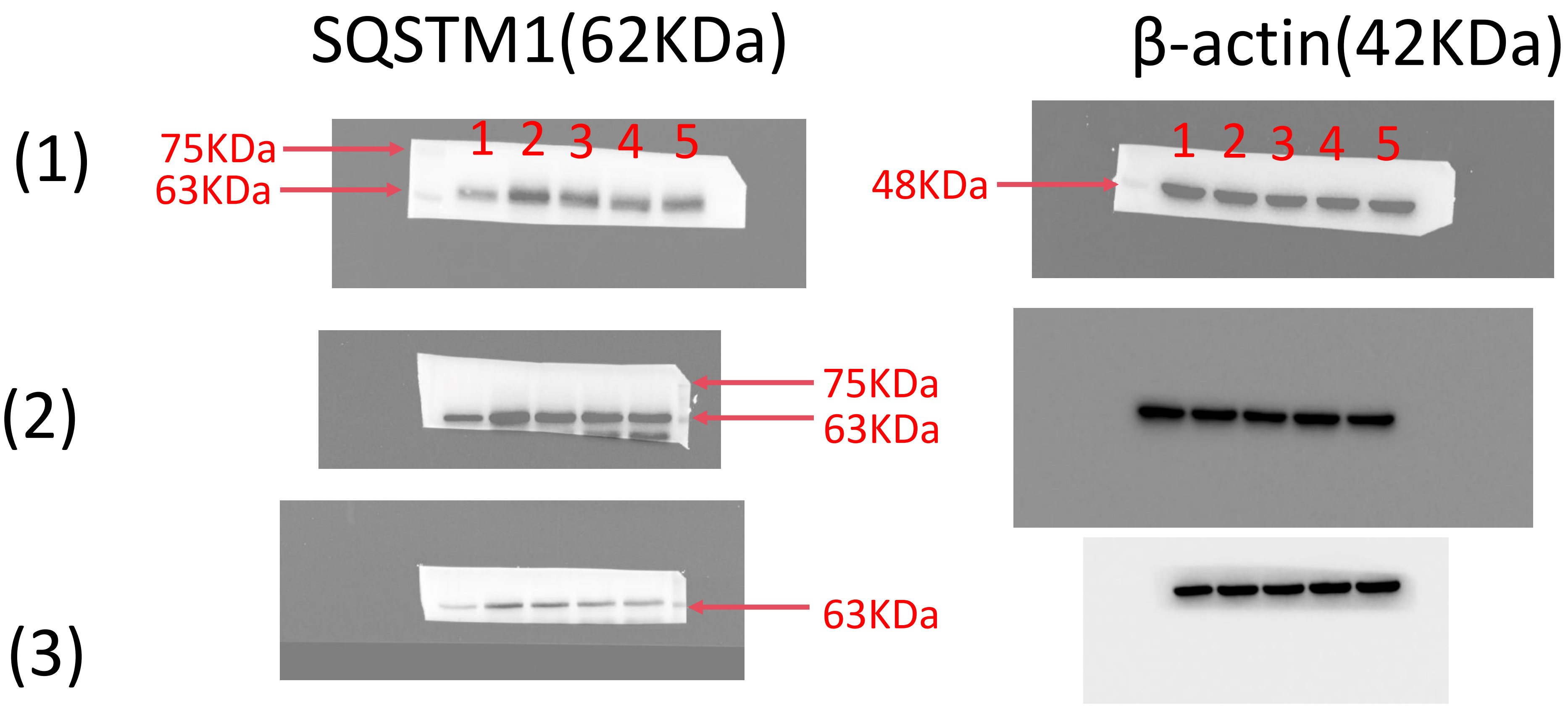

1: Control
2:LPS
3:LPS+FMN(30 μM)
4:LPS+FMN(30 μM)+WY(20 μM)
5:LPS+WY(20 μM)

The protein band marker(PR1920) was purchased from the Solarbio (Beijing, China)

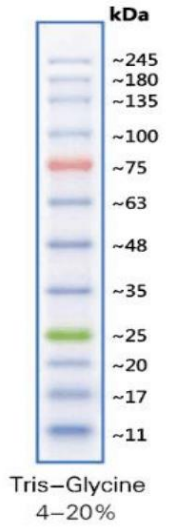

Fig6 A

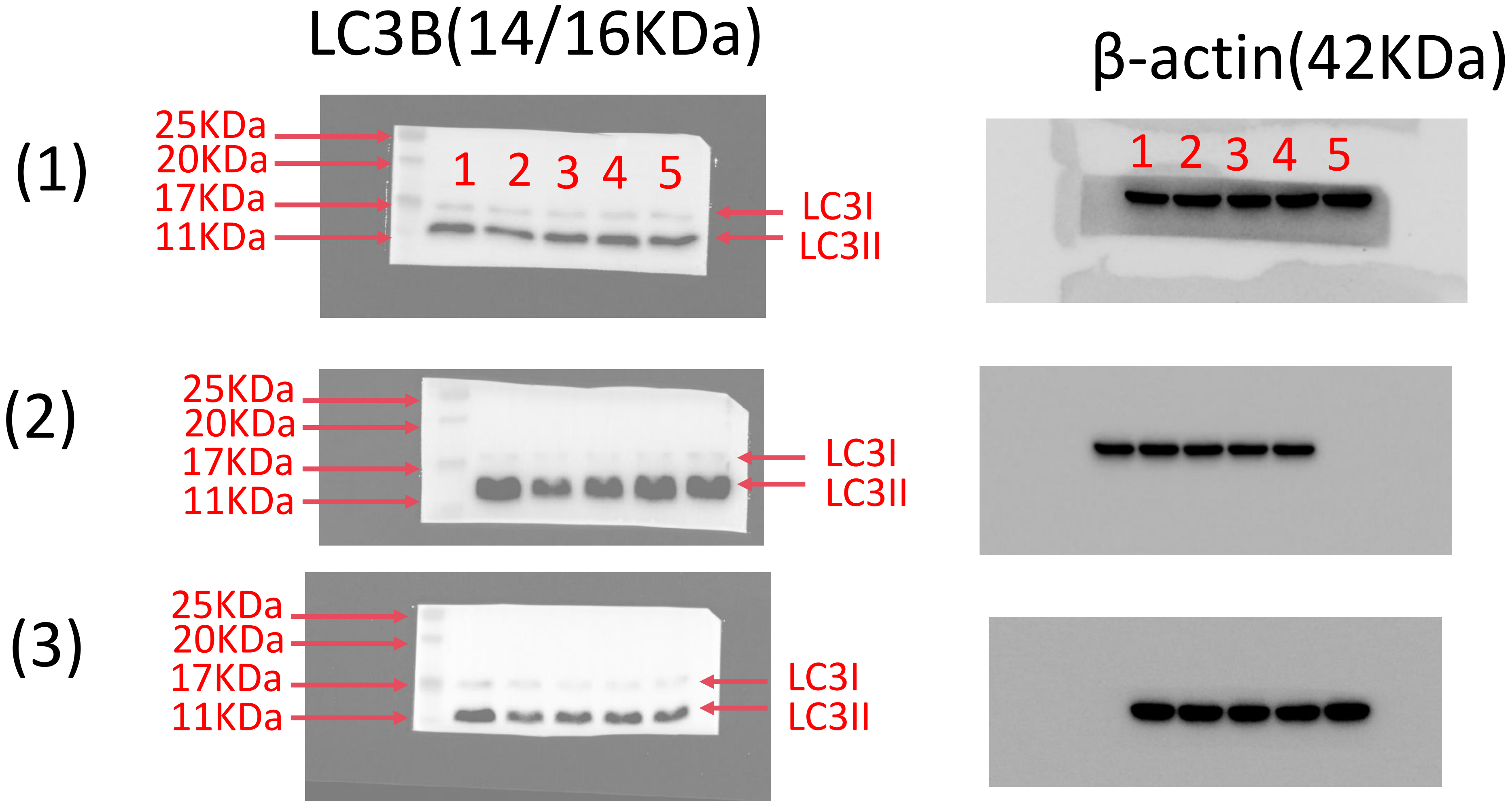

1: Control

2:LPS

3:LPS+FMN(30 μM)

4:LPS+FMN(30 μM)+WY(20 μM)

5:LPS+WY(20 μM)

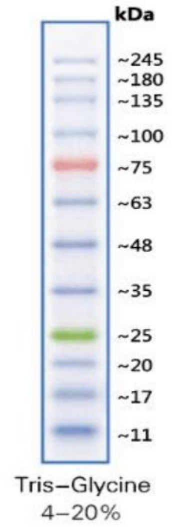

The protein band marker(PR1920) was purchased from the Solarbio (Beijing, China)

Fig6 C

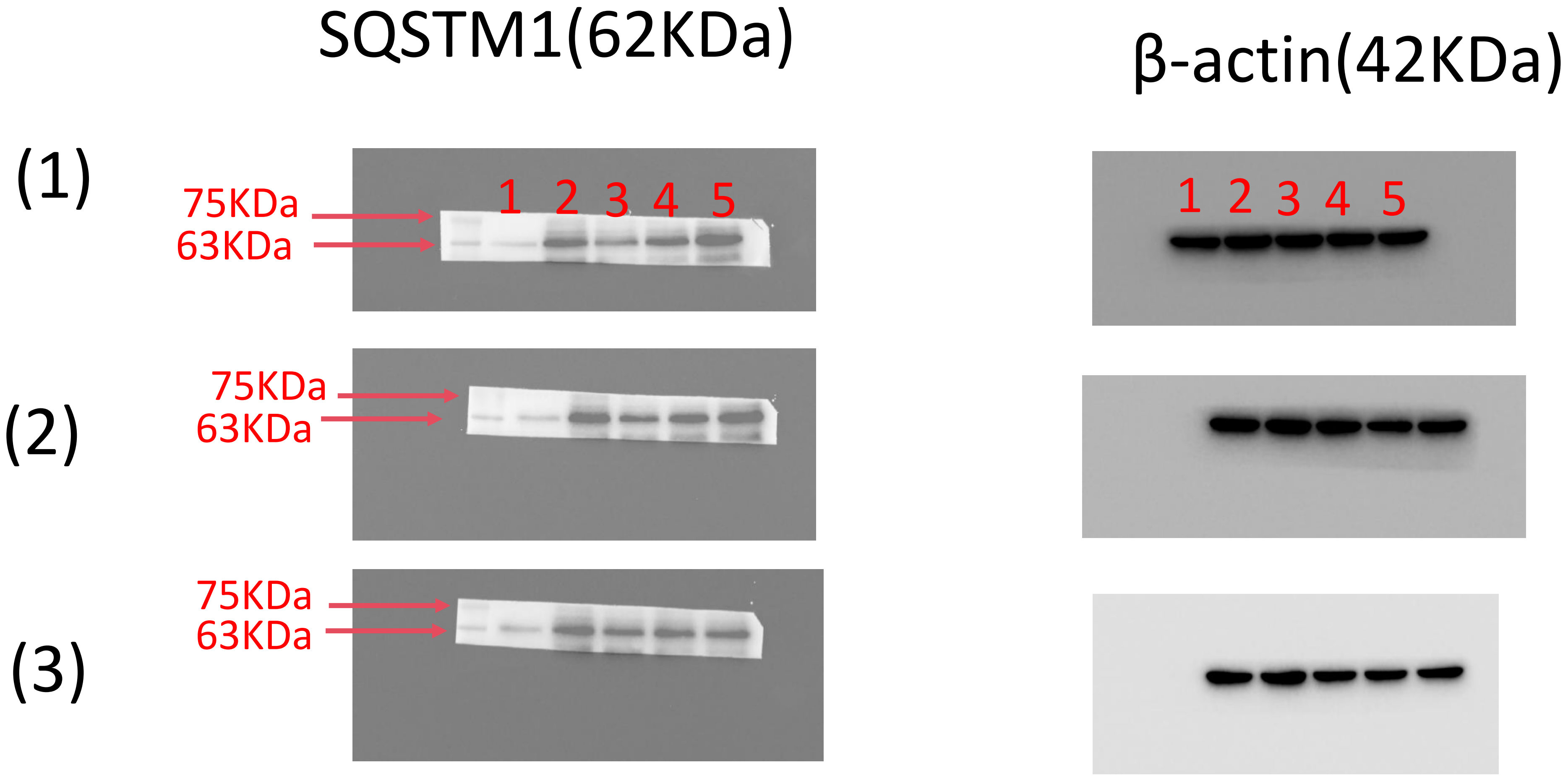

1: Control
2:LPS
3:LPS+FMN(30 μM)
4:LPS+FMN(30 μM)+GW(5 μM)
5:LPS+GW(5 μM)

The protein band marker(PR1920) was purchased from the Solarbio (Beijing, China)

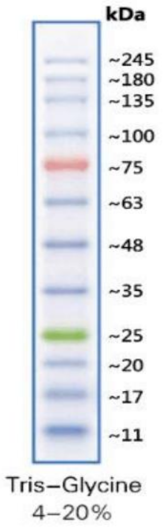

Fig6 C

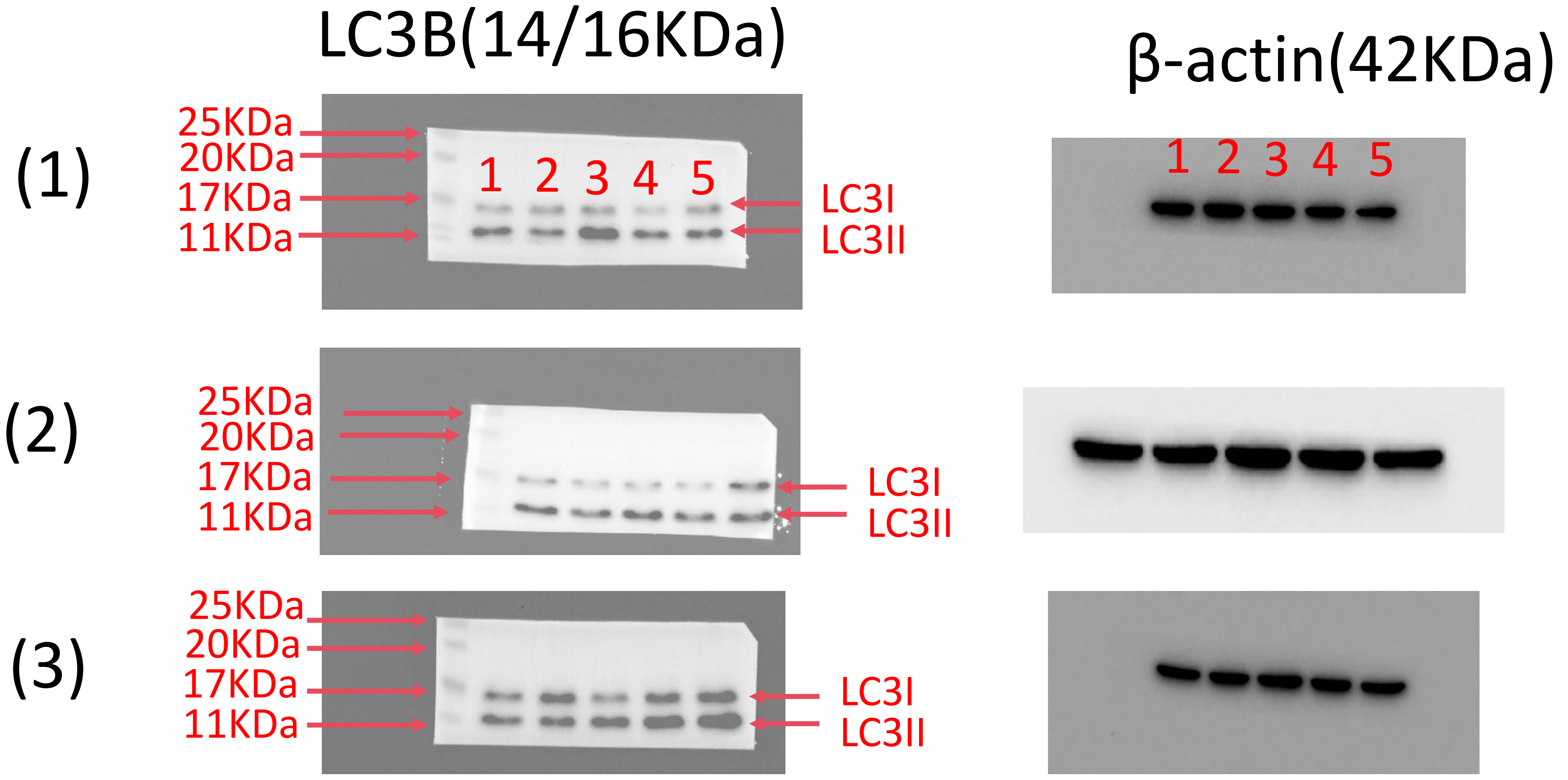

1: Control
2:LPS
3:LPS+FMN(30 μM)
4:LPS+FMN(30 μM)+GW(5 μM)
5:LPS+GW(5 μM)

The protein band marker(PR1920) was purchased from the Solarbio (Beijing, China)

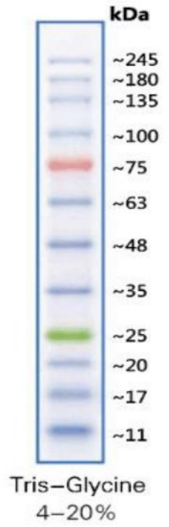

Fig6 F

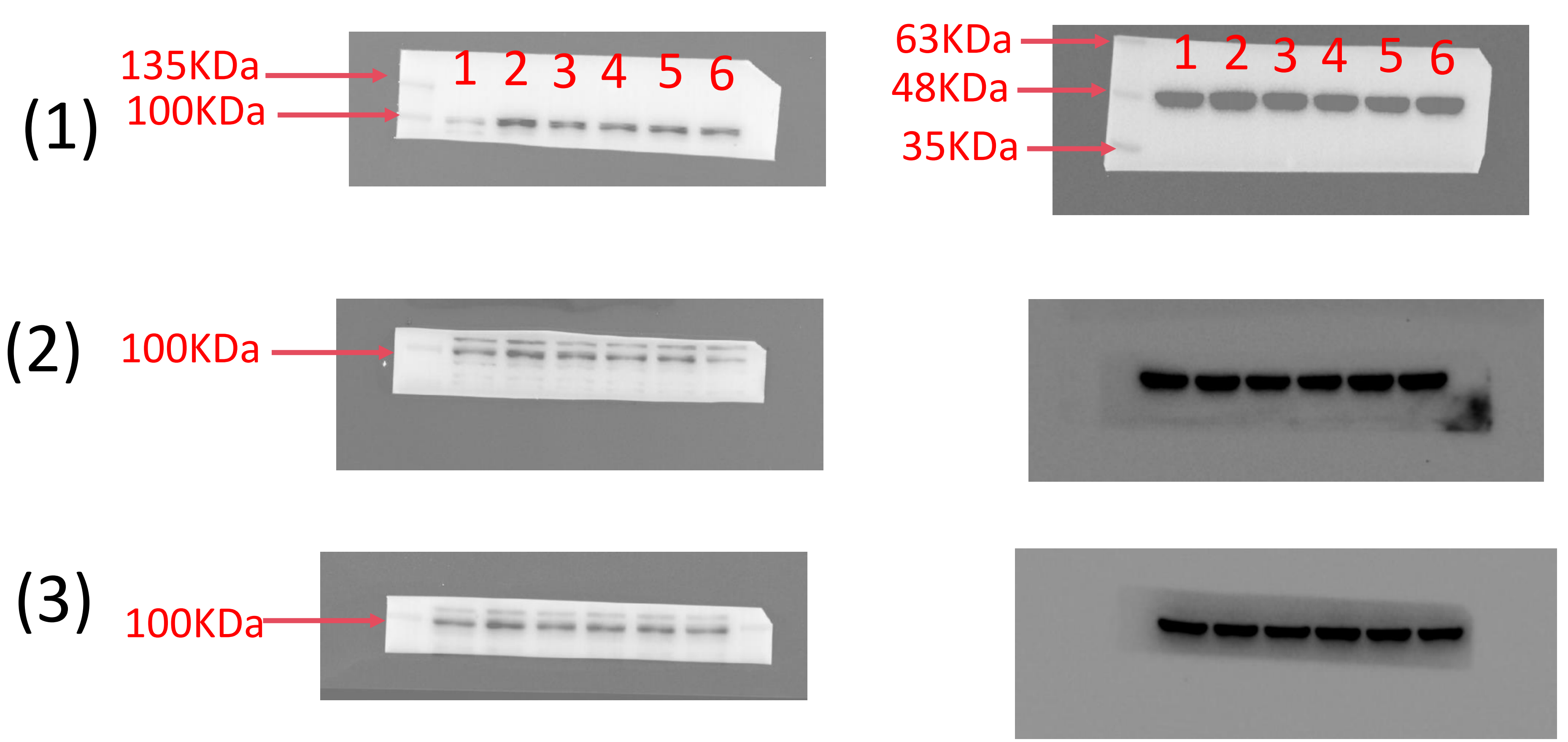

1: Control
2:LPS
3:LPS+Mino(20 μM)
4:LPS+FMN(30 μM)
5:LPS+FMN(30 μM)+WY(20 μM)
6:LPS+WY(20 μM)

The protein band marker(PR1920) was purchased from the Solarbio (Beijing, China)

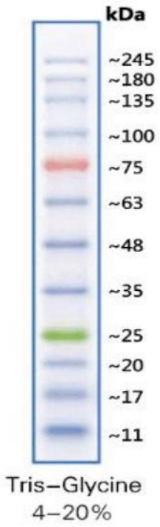

Fig6 F

CD206(166KDa) β-actin(42KDa)

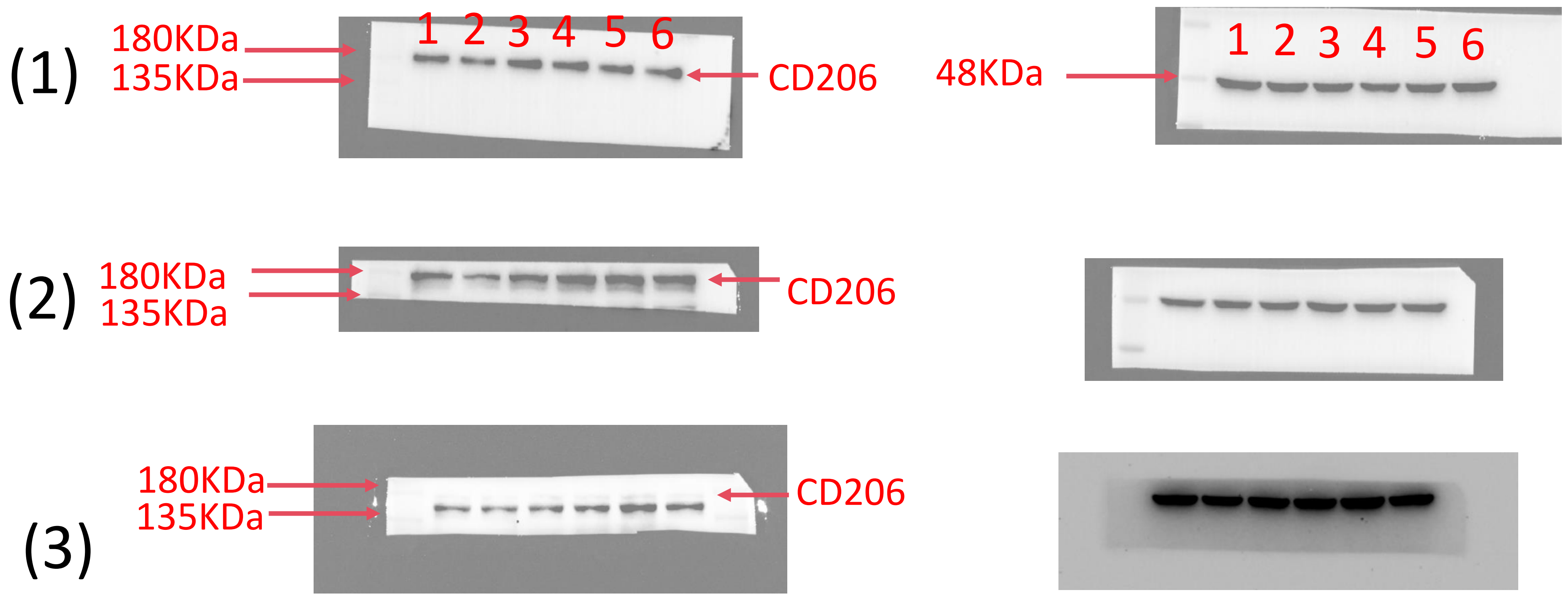

1: Control
2:LPS
3:LPS+Mino(20 μM)
4:LPS+FMN(30 μM)
5:LPS+FMN(30 μM)+WY(20 μM)
6:LPS+WY(20 μM)

The protein band marker(PR1920) was purchased from the Solarbio (Beijing, China)

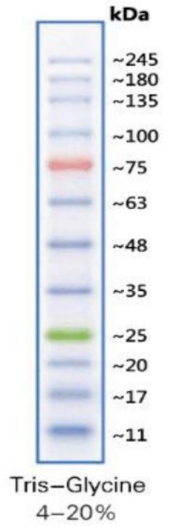

Fig6 F

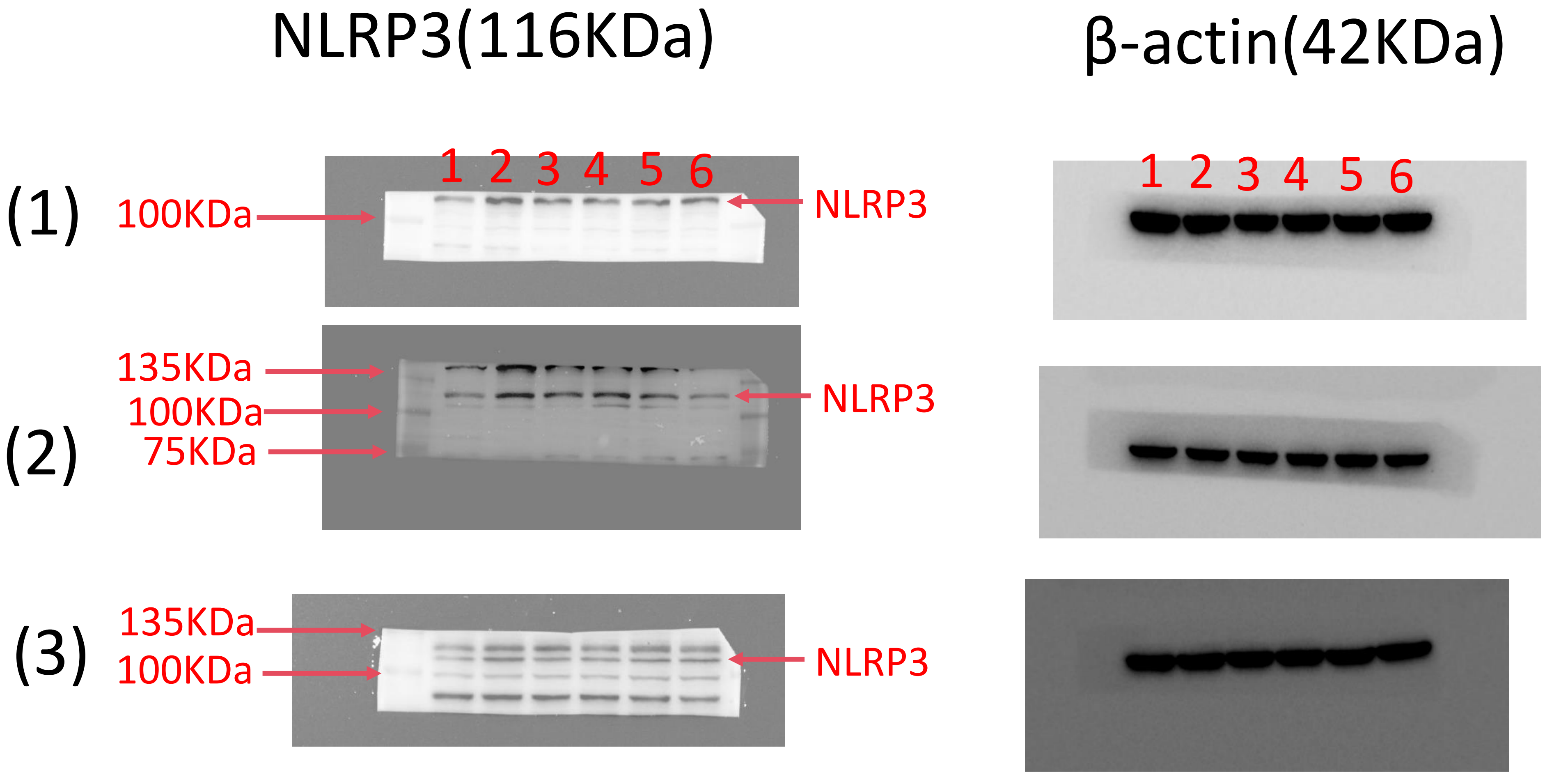

1: Control
2:LPS
3:LPS+Mino(20 μM)
4:LPS+FMN(30 μM)
5:LPS+FMN(30 μM)+WY(20 μM)
6:LPS+WY(20 μM)

The protein band marker(PR1920) was purchased from the Solarbio (Beijing, China)

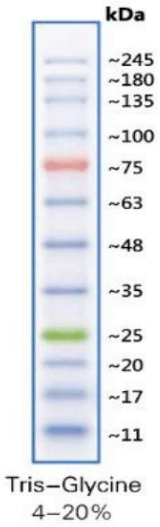

Fig6 H

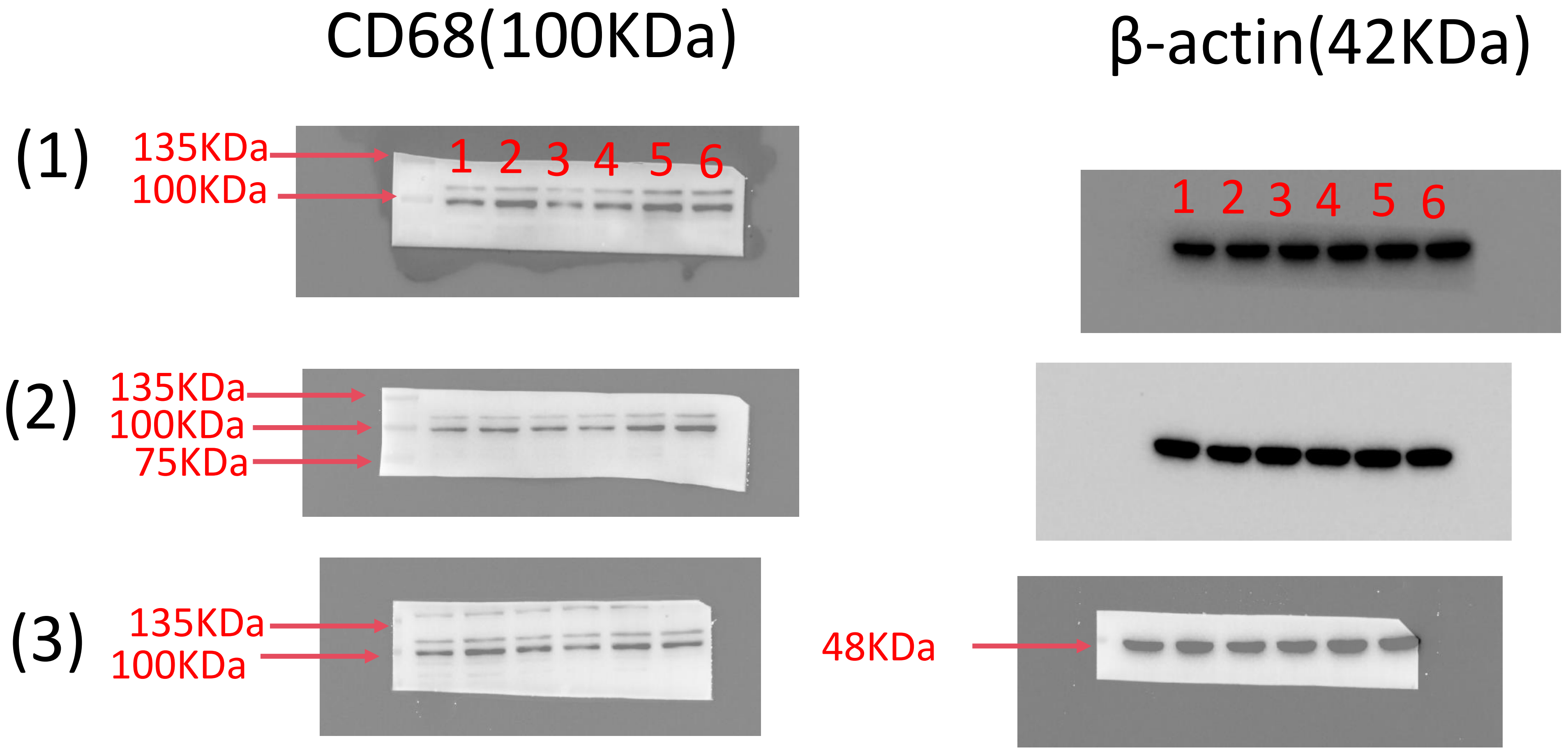

1: Control
2:LPS
3:LPS+Mino(20 μM)
4:LPS+FMN(30 μM)
5:LPS+FMN(30 μM)+GW(5 μM)
6:LPS+GW(5 μM)

The protein band marker(PR1920) was purchased from the Solarbio (Beijing, China)

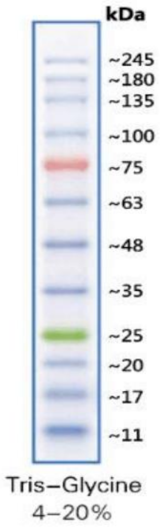

Fig6 H

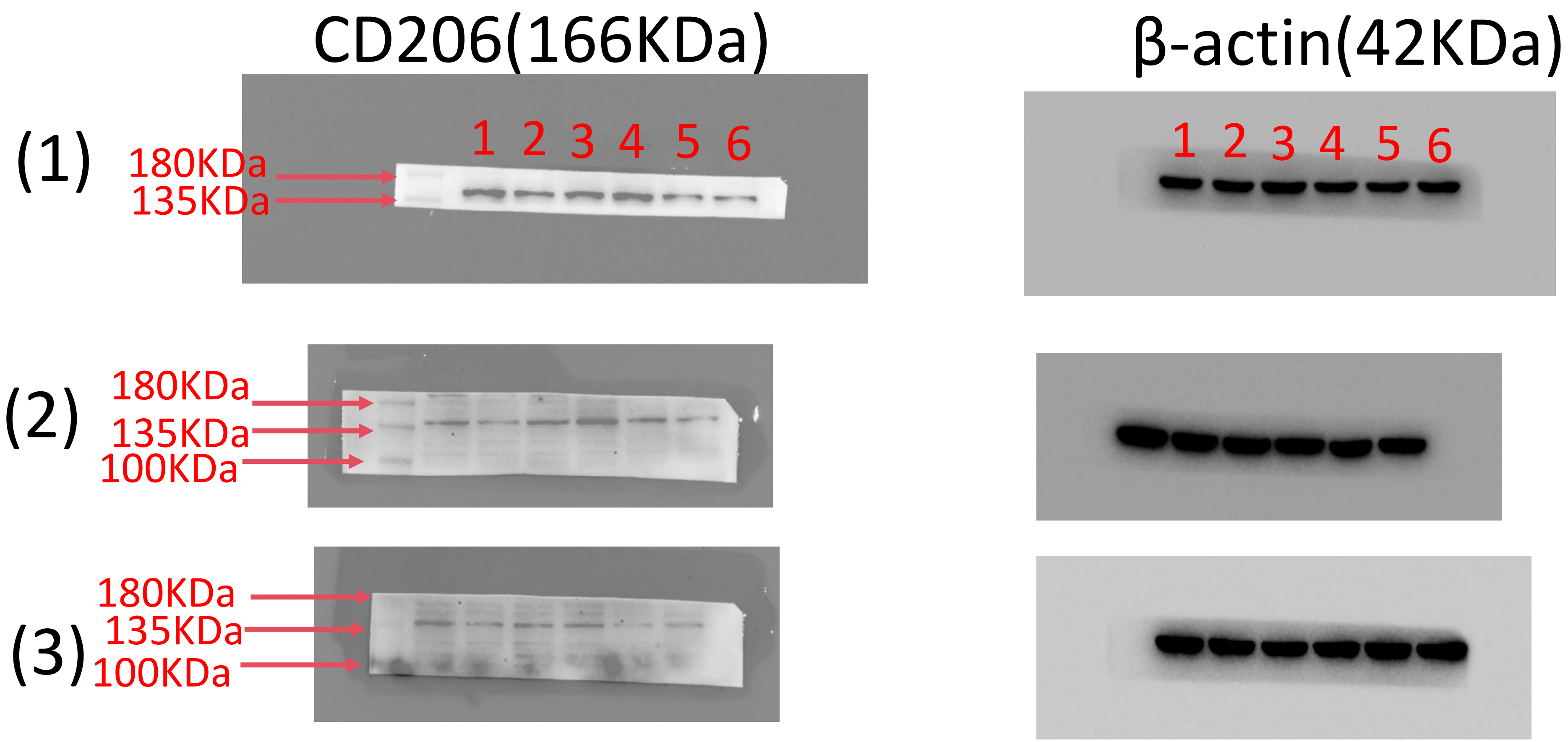

1: Control
2:LPS
3:LPS+Mino(20 μM)
4:LPS+FMN(30 μM)
5:LPS+FMN(30 μM)+GW(5 μM)
6:LPS+GW(5 μM)

The protein band marker(PR1920) was purchased from the Solarbio (Beijing, China)

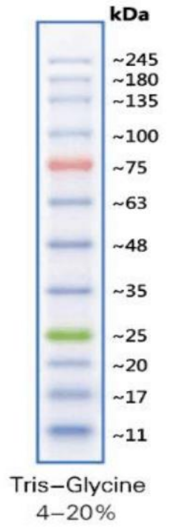

Fig6 H

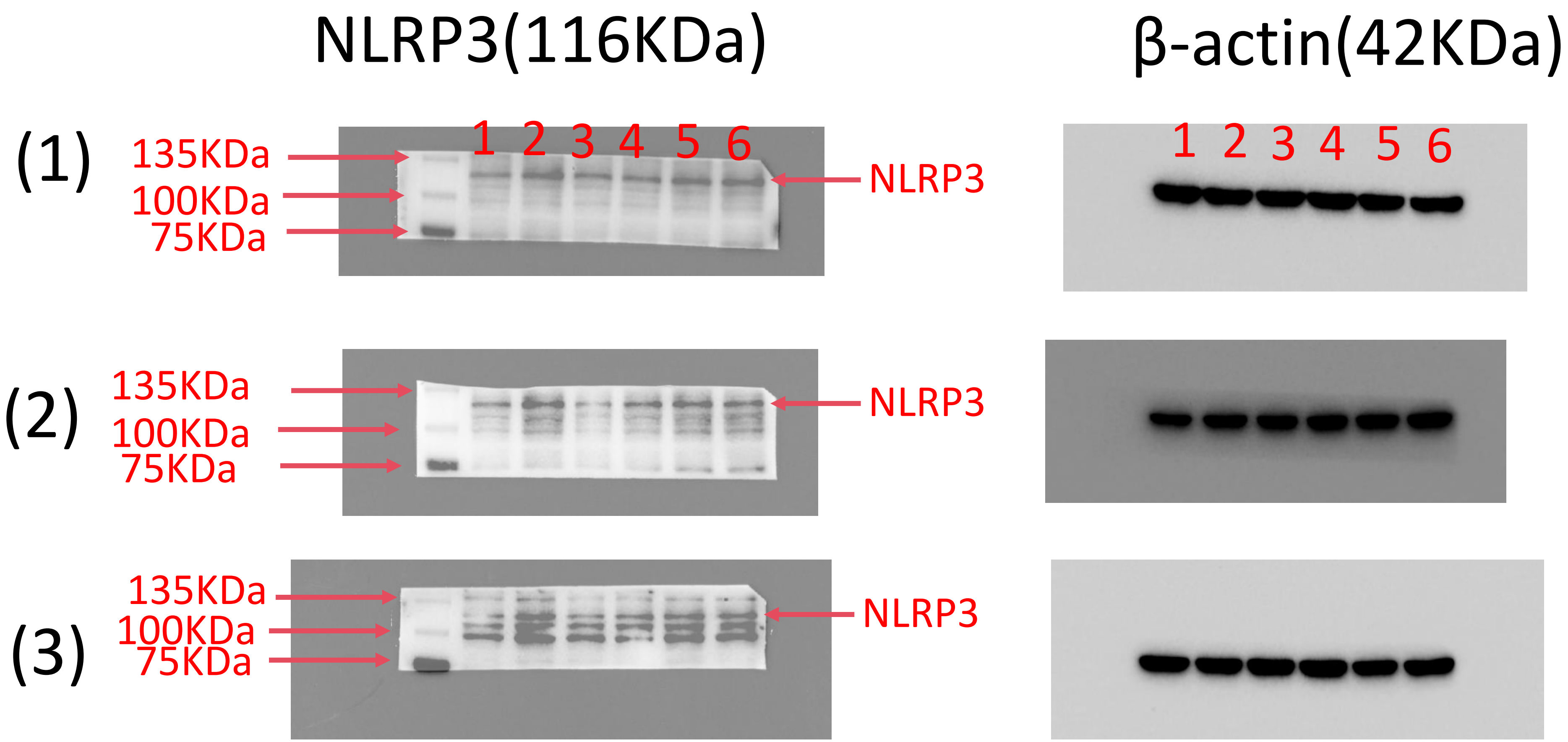

1: Control
2:LPS
3:LPS+Mino(20 μM)
4:LPS+FMN(30 μM)
5:LPS+FMN(30 μM)+GW(5 μM)
6:LPS+GW(5 μM)

The protein band marker(PR1920) was purchased from the Solarbio (Beijing, China)

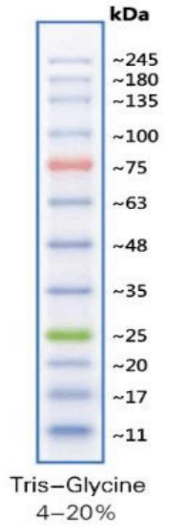

Fig7 I

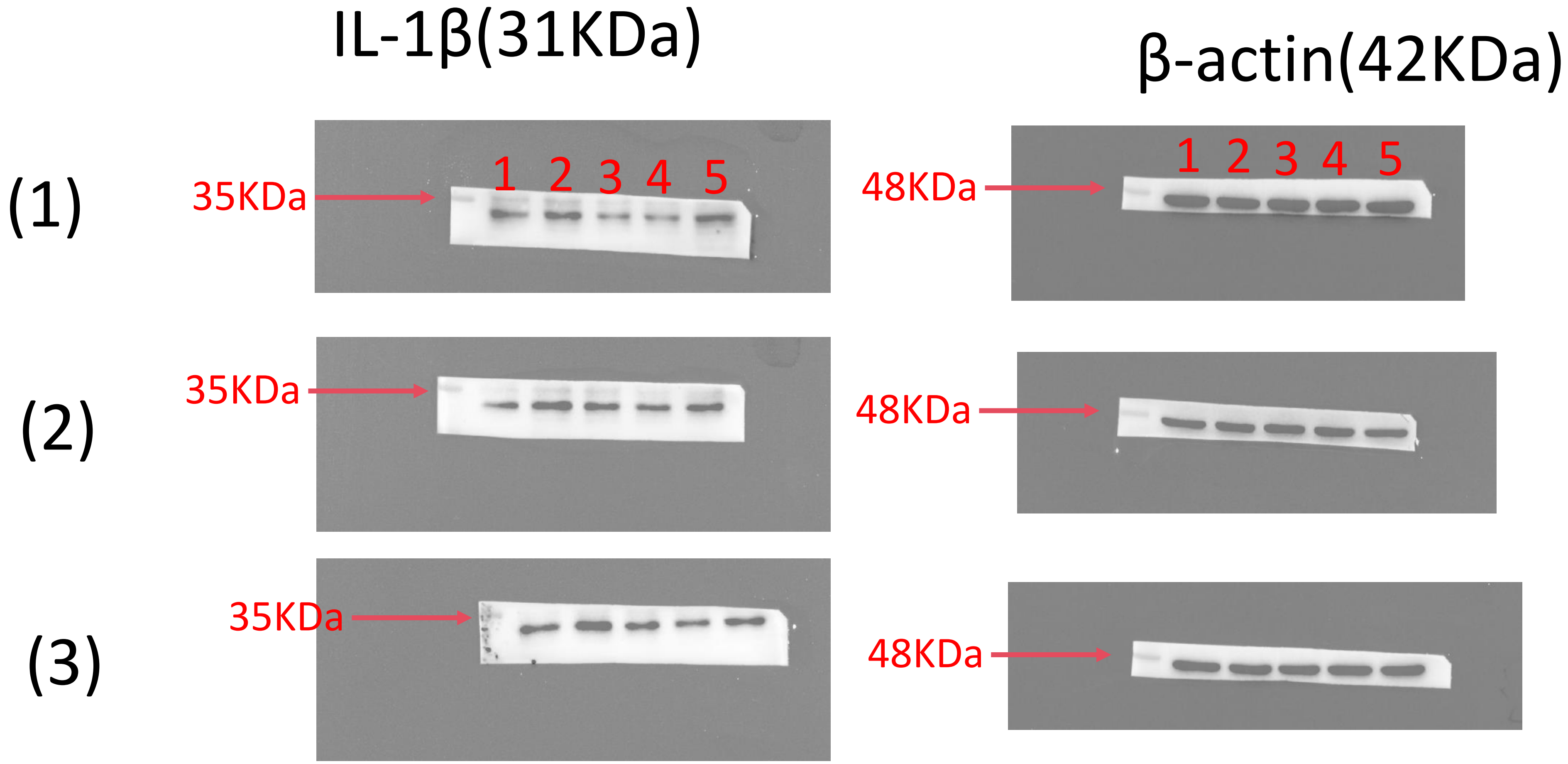

1: Control
2:LPS
3:LPS+WY(10 mg/kg)
4:LPS+FMN(40 mg/kg)
5:LPS+FMN(40 mg/kg)+GW(2 mg/kg)

The protein band marker(PR1920) was purchased from the Solarbio (Beijing, China)

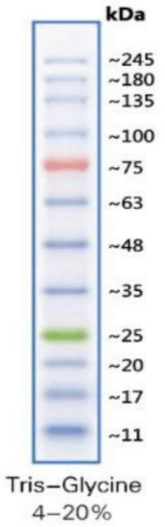

Fig7 I

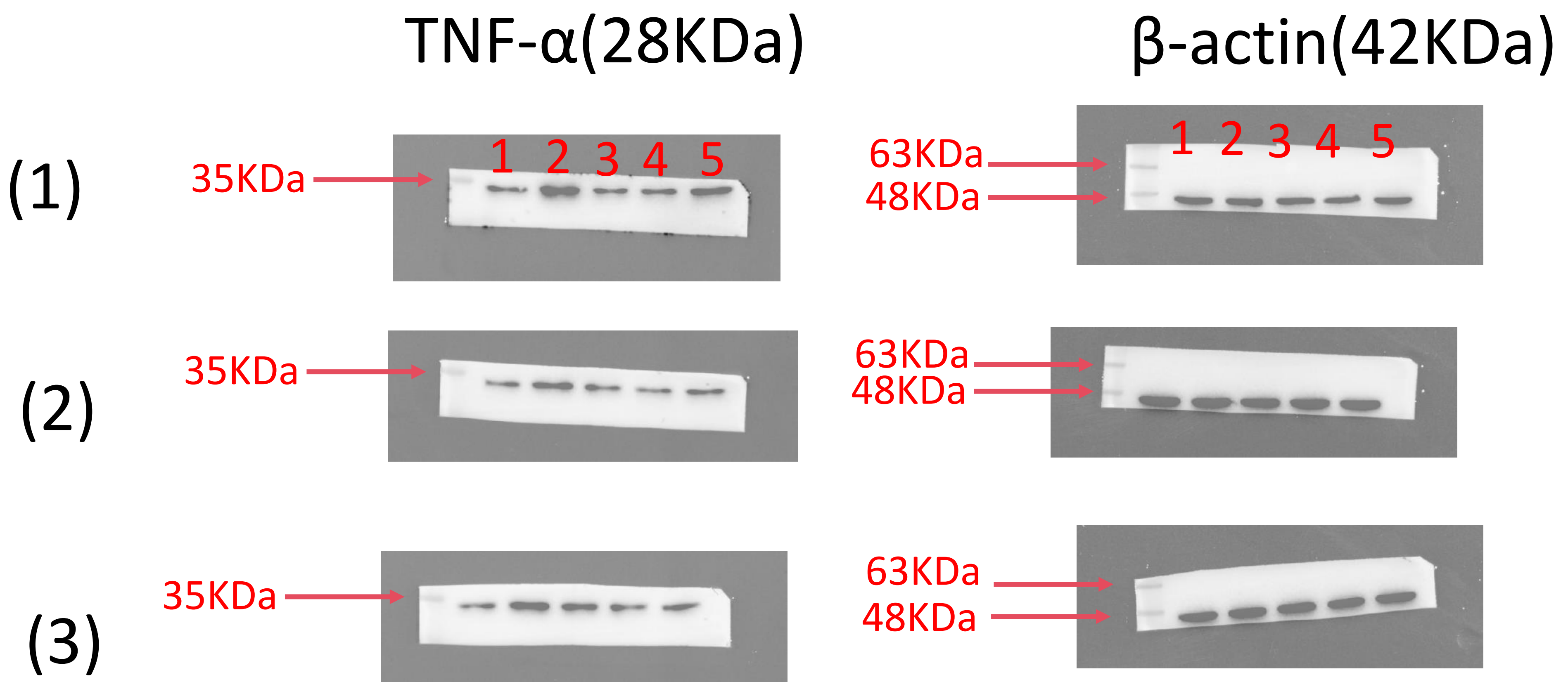

1: Control
2:LPS
3:LPS+WY(10 mg/kg)
4:LPS+FMN(40 mg/kg)
5:LPS+FMN(40 mg/kg)+GW(2 mg/kg)

The protein band marker(PR1920) was purchased from the Solarbio (Beijing, China)

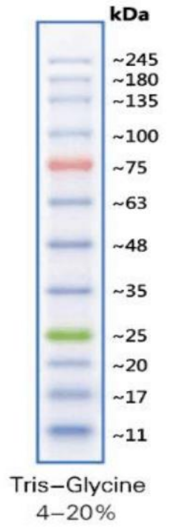

Fig7 I

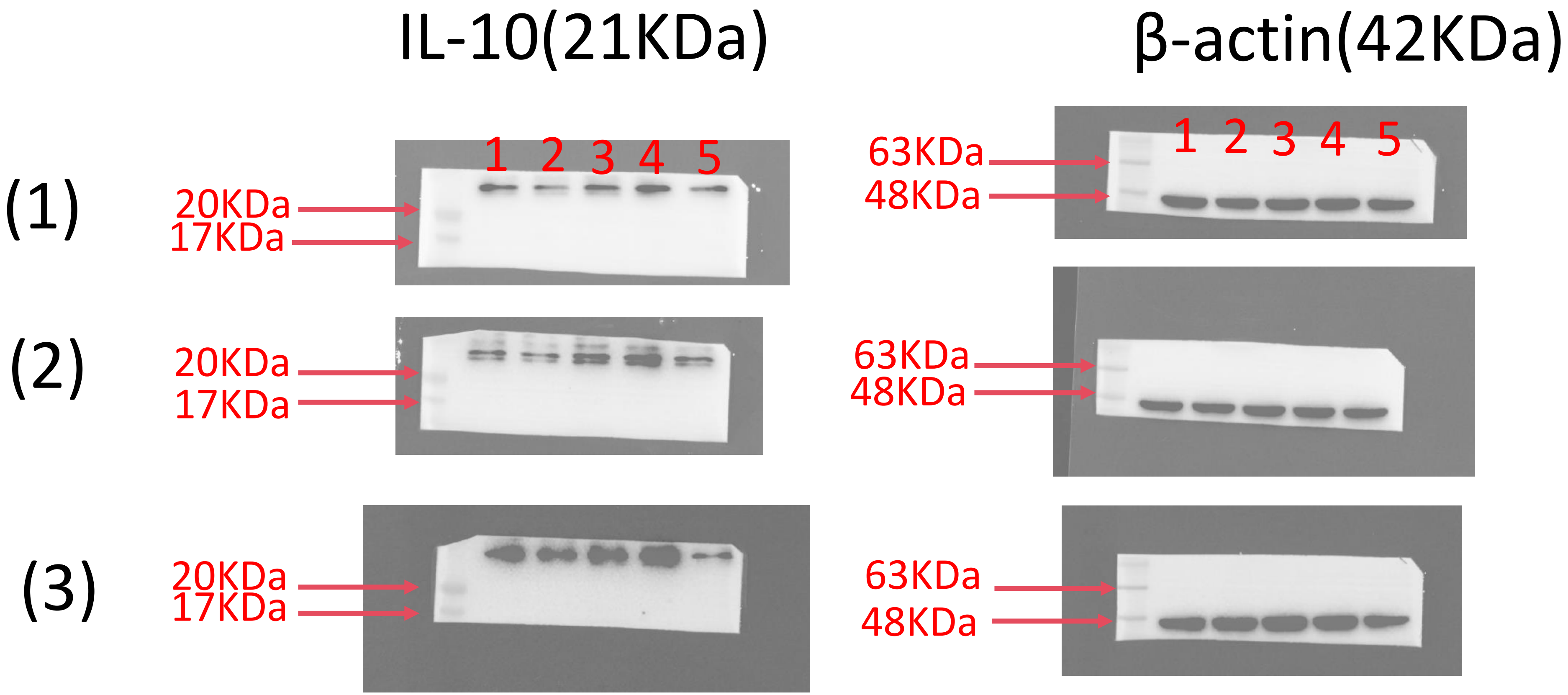

1: Control

2:LPS

3:LPS+WY(10 mg/kg)

4:LPS+FMN(40 mg/kg)

5:LPS+FMN(40 mg/kg)+GW(2 mg/kg)

The protein band marker(PR1920) was purchased from the Solarbio (Beijing, China)

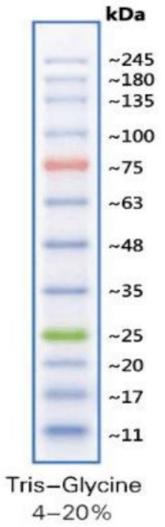

Fig8 E

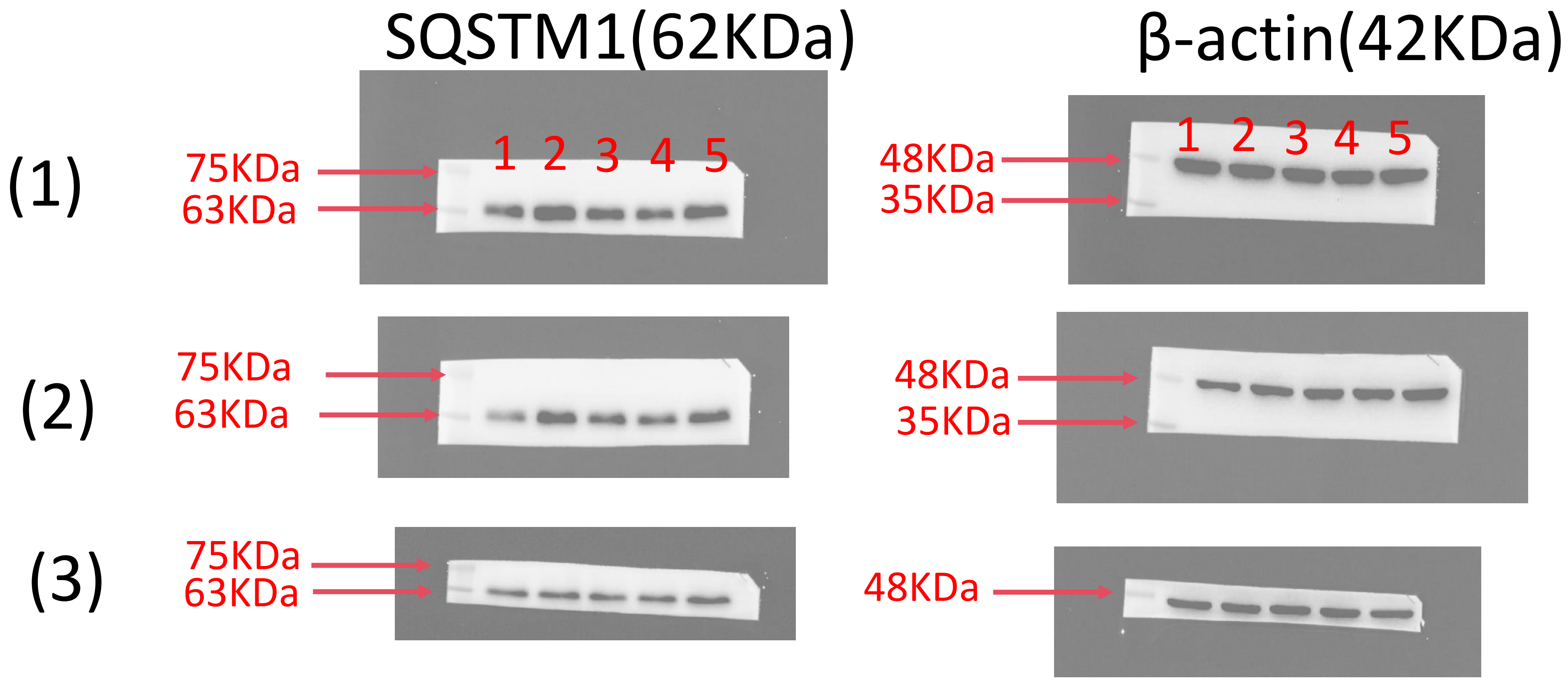

1: Control
2:LPS
3:LPS+WY(10 mg/kg)
4:LPS+FMN(40 mg/kg)
5:LPS+FMN(40 mg/kg)+GW(2 mg/kg)

The protein band marker(PR1920) was purchased from the Solarbio (Beijing, China)

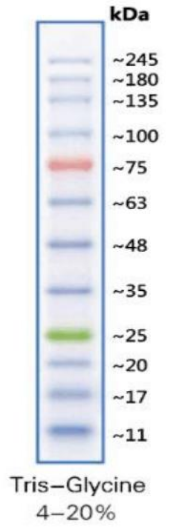

Fig8 E

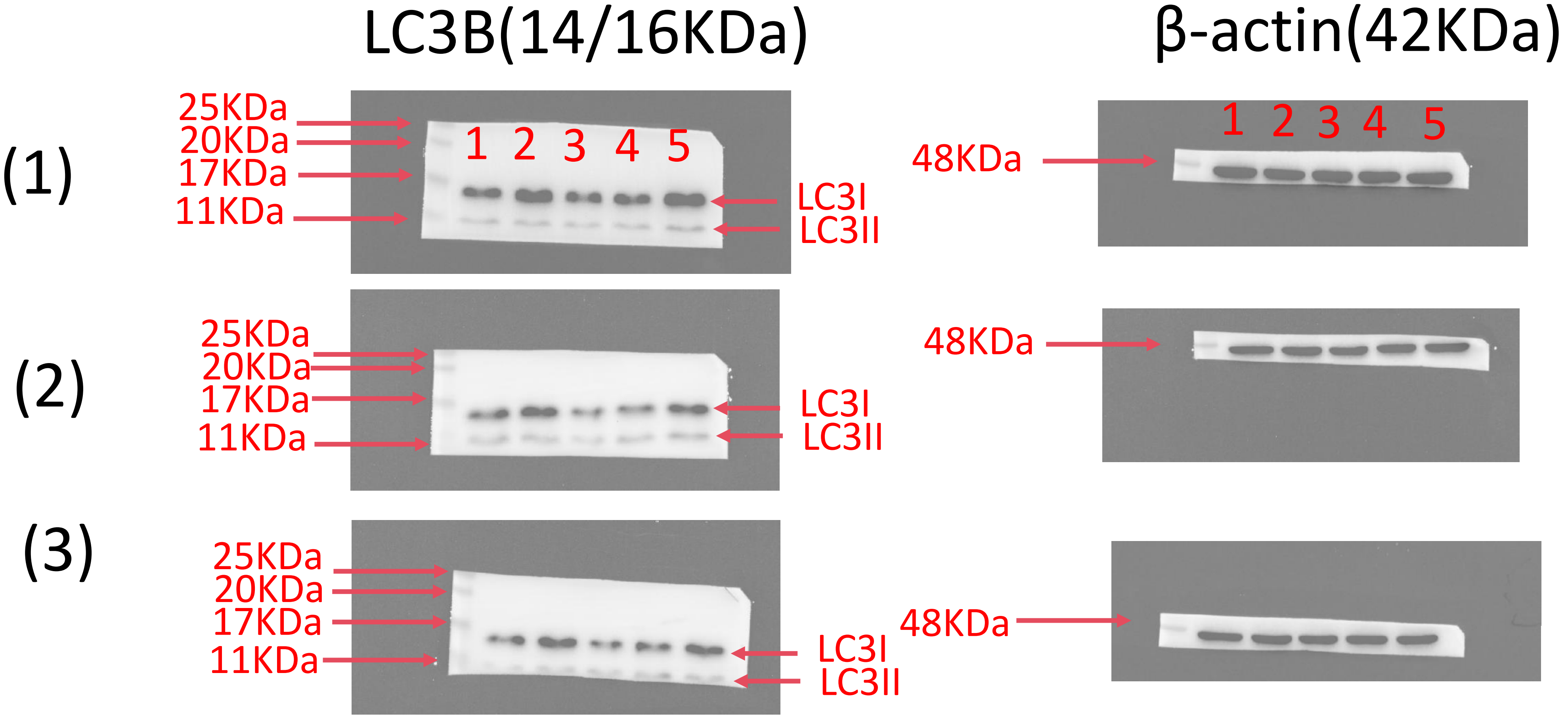

1: Control
2:LPS
3:LPS+WY(10 mg/kg)
4:LPS+FMN(40 mg/kg)
5:LPS+FMN(40 mg/kg)+GW(2 mg/kg)

The protein band marker(PR1920) was purchased from the Solarbio (Beijing, China)

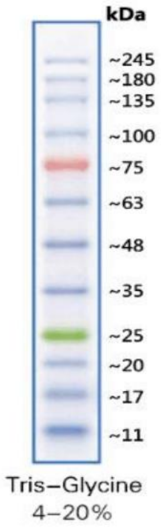

Fig8 M

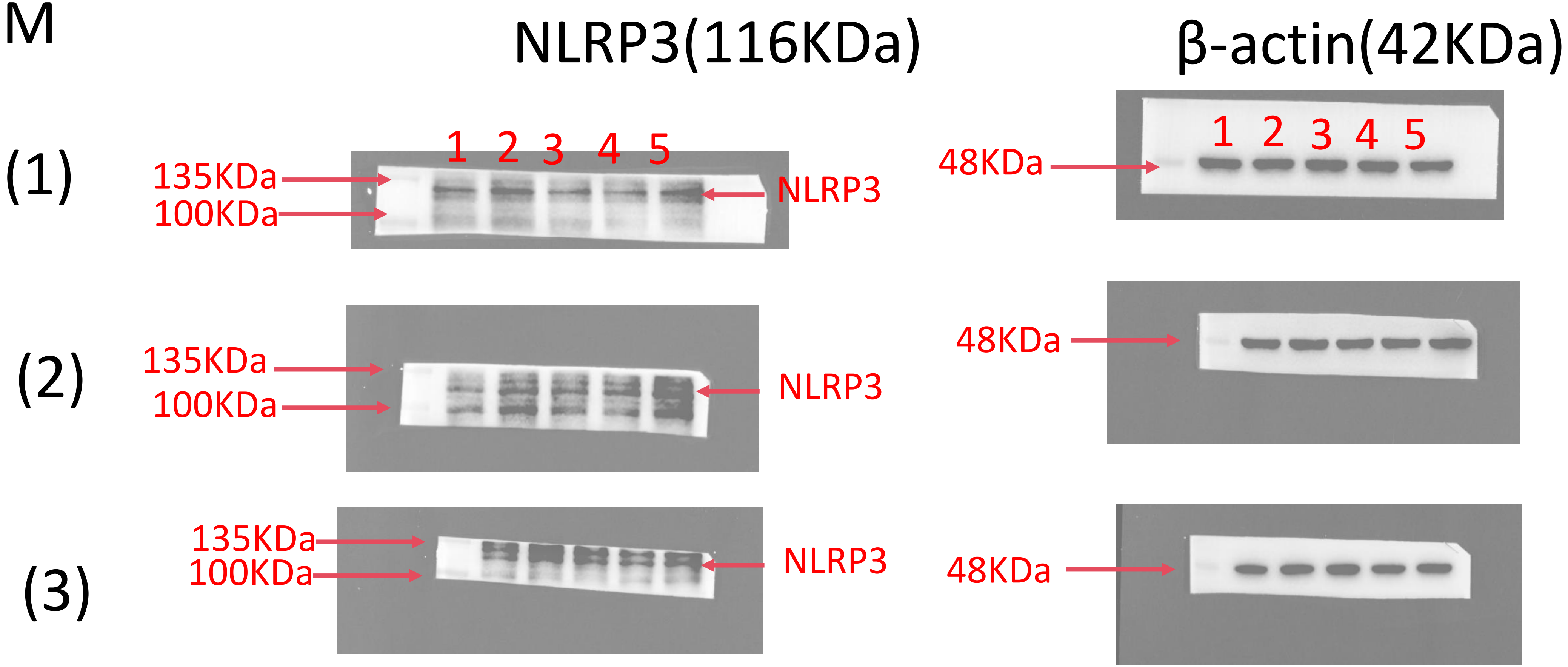

1: Control
2:LPS
3:LPS+WY(10 mg/kg)
4:LPS+FMN(40 mg/kg)
5:LPS+FMN(40 mg/kg)+GW(2 mg/kg)

The protein band marker(PR1920) was purchased from the Solarbio (Beijing, China)

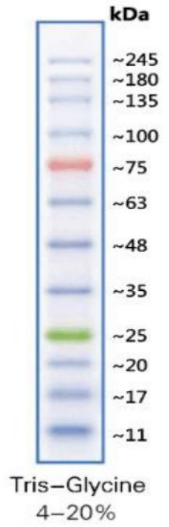

Fig8 M

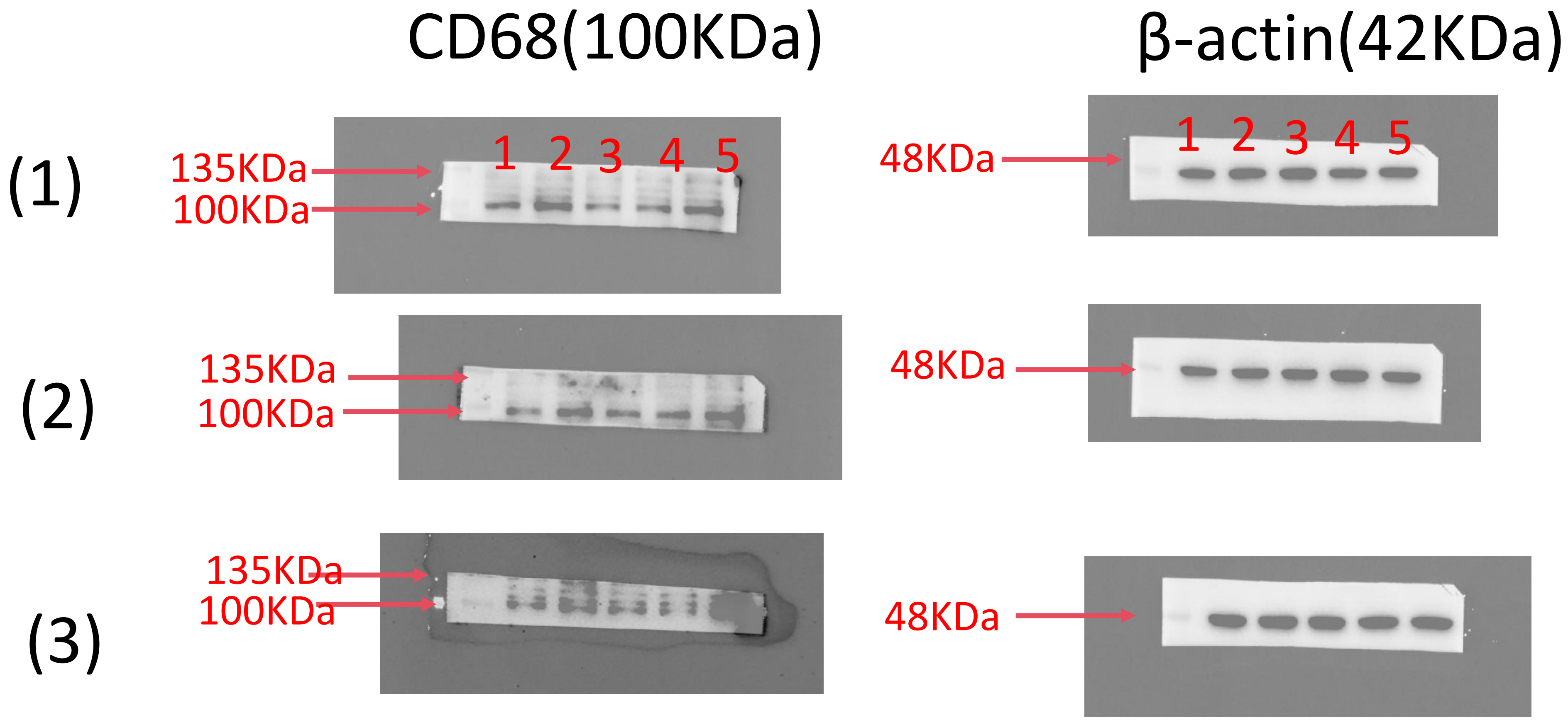

1: Control
2:LPS
3:LPS+WY(10 mg/kg)
4:LPS+FMN(40 mg/kg)
5:LPS+FMN(40 mg/kg)+GW(2 mg/kg)

The protein band marker(PR1920) was purchased from the Solarbio (Beijing, China)

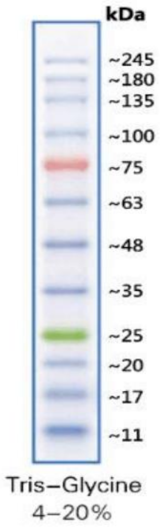

Fig8 M

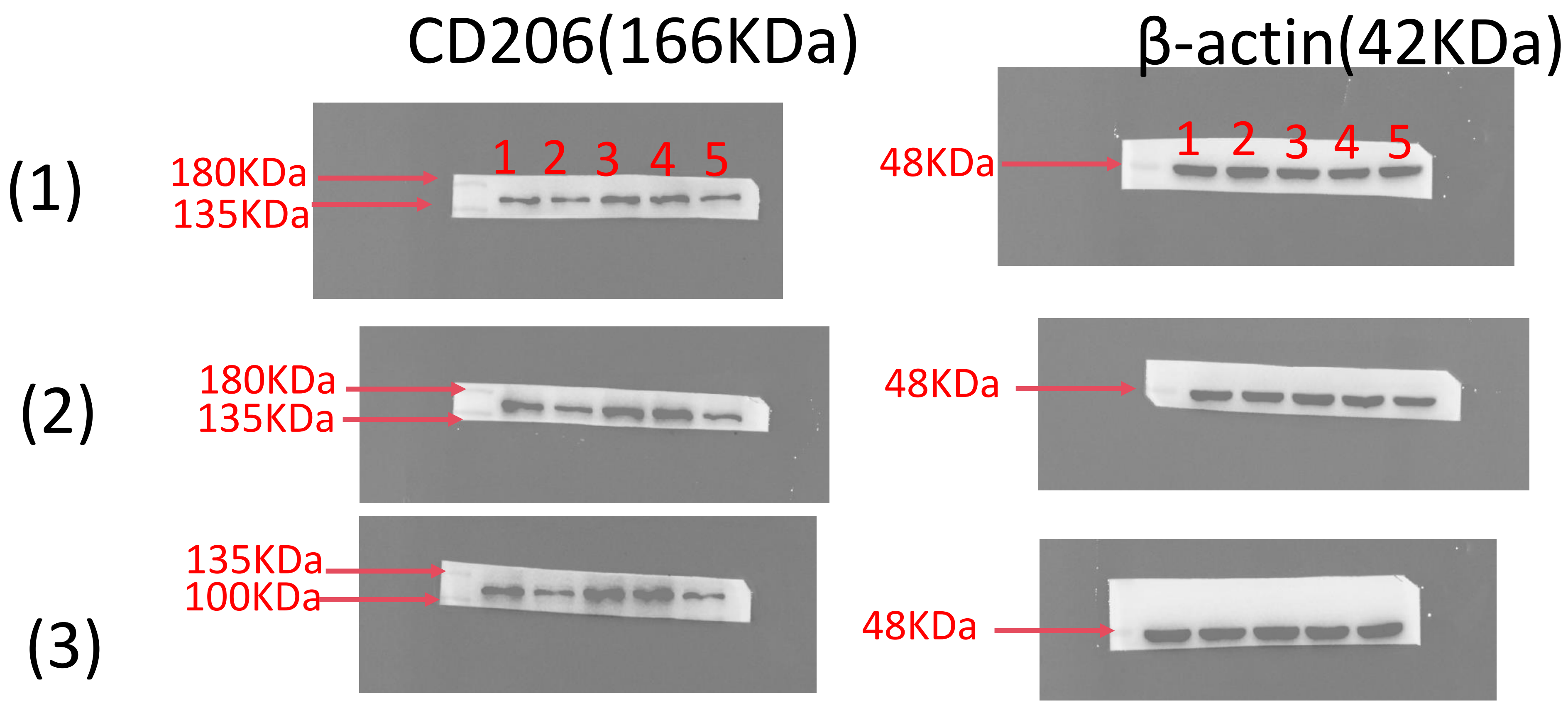

1: Control
2:LPS
3:LPS+WY(10 mg/kg)
4:LPS+FMN(40 mg/kg)
5:LPS+FMN(40 mg/kg)+GW(2 mg/kg)

The protein band marker(PR1920) was purchased from the Solarbio (Beijing, China)

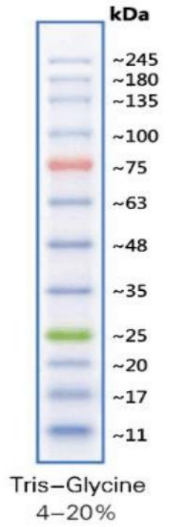

Supplement: Supplementary file 2 — Supplementary Material 2 [file 10020_2025_1217_MOESM2_ESM.pdf]
