# Supplementary figures and images for: Formononetin ameliorates depression-like behaviors through rebalancing microglia M1/M2 polarization and inhibiting NLRP3 inflammasome: involvement of activating PPARα-mediated autophagy
Source: Mol Med. 2025 Apr 24;31:153. doi: 10.1186/s10020-025-01217-2 (PMC12023581; doi:10.1186/s10020-025-01217-2)

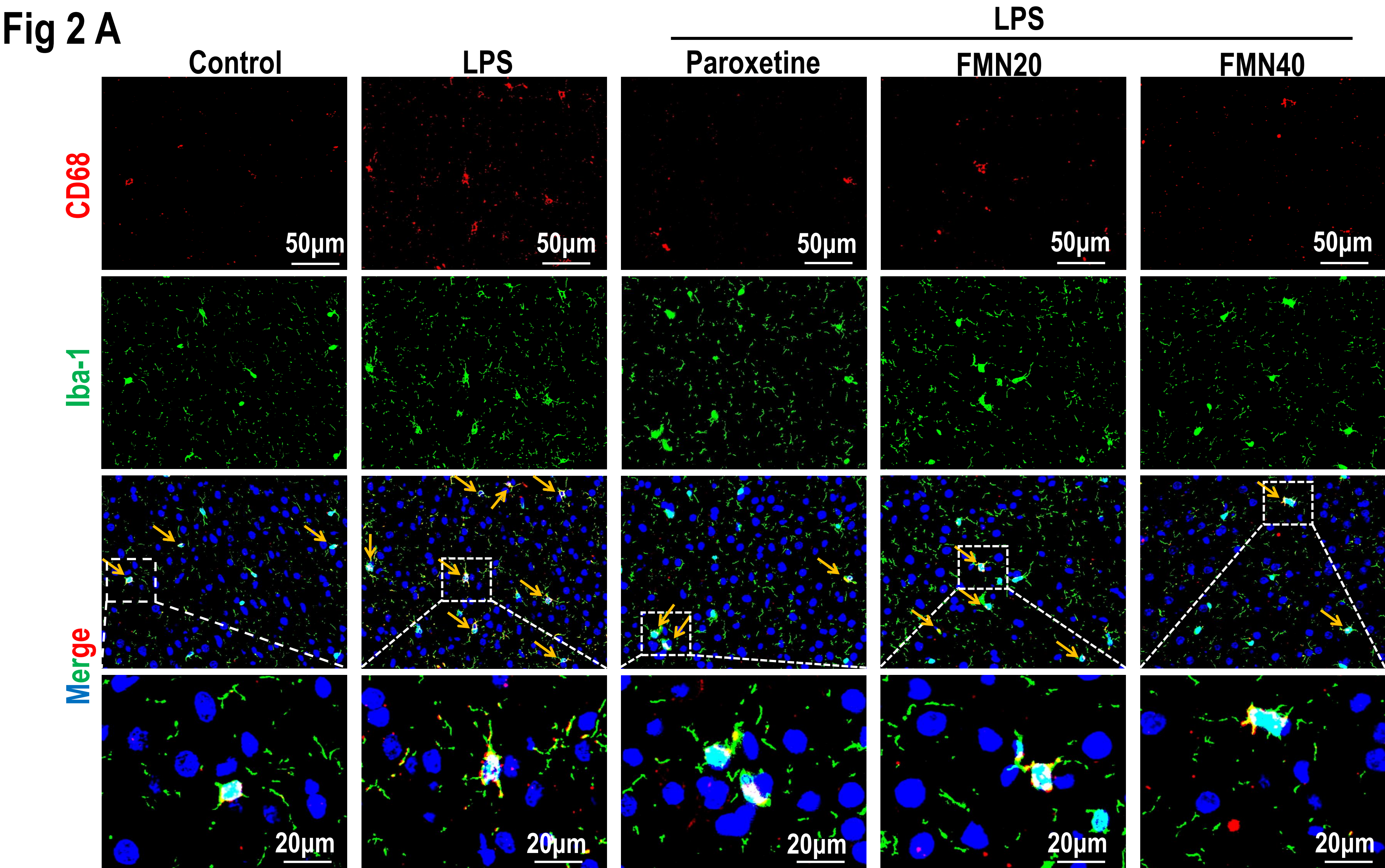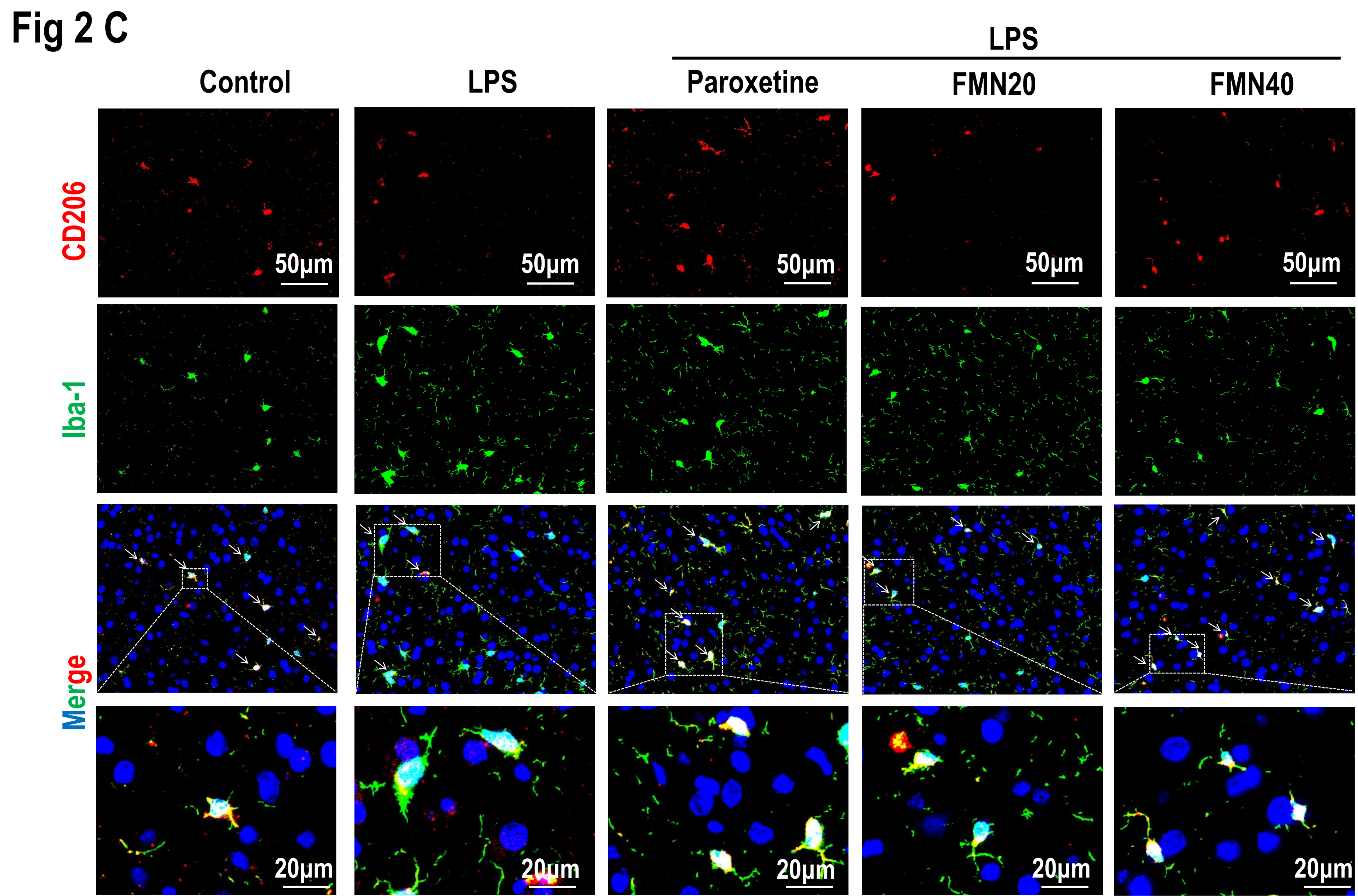

Fig 2 H

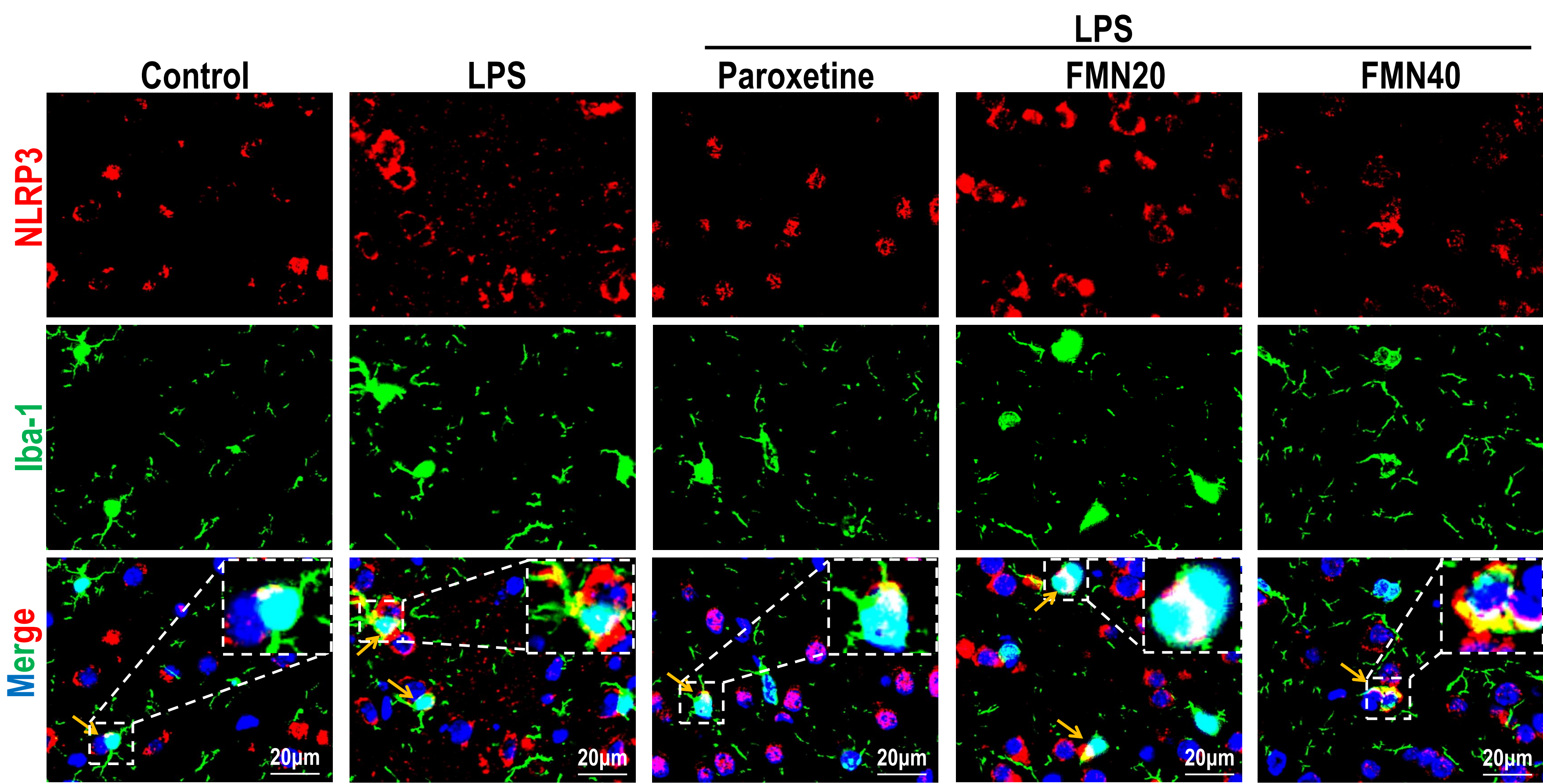

Fig 2 J

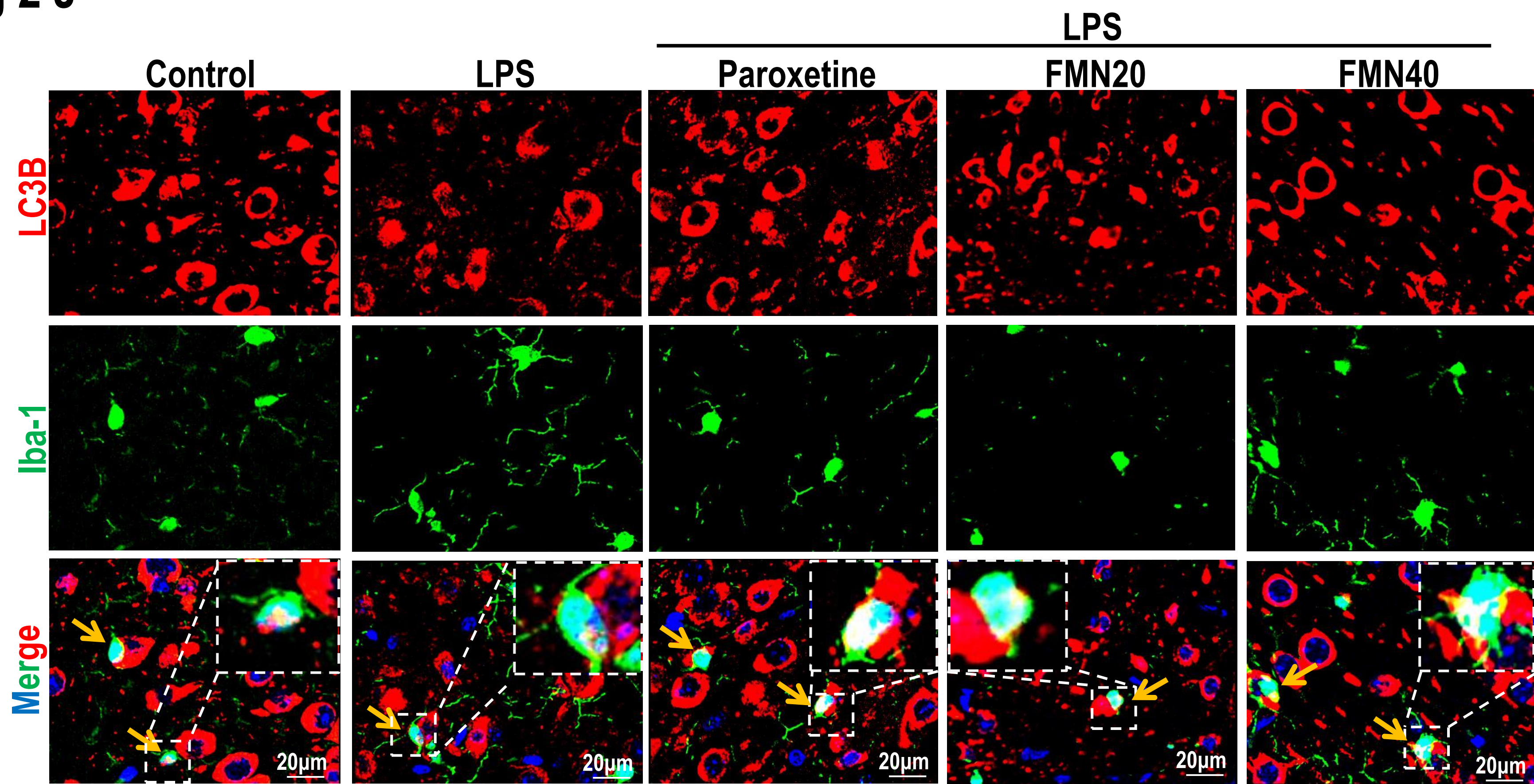

Fig 3 F

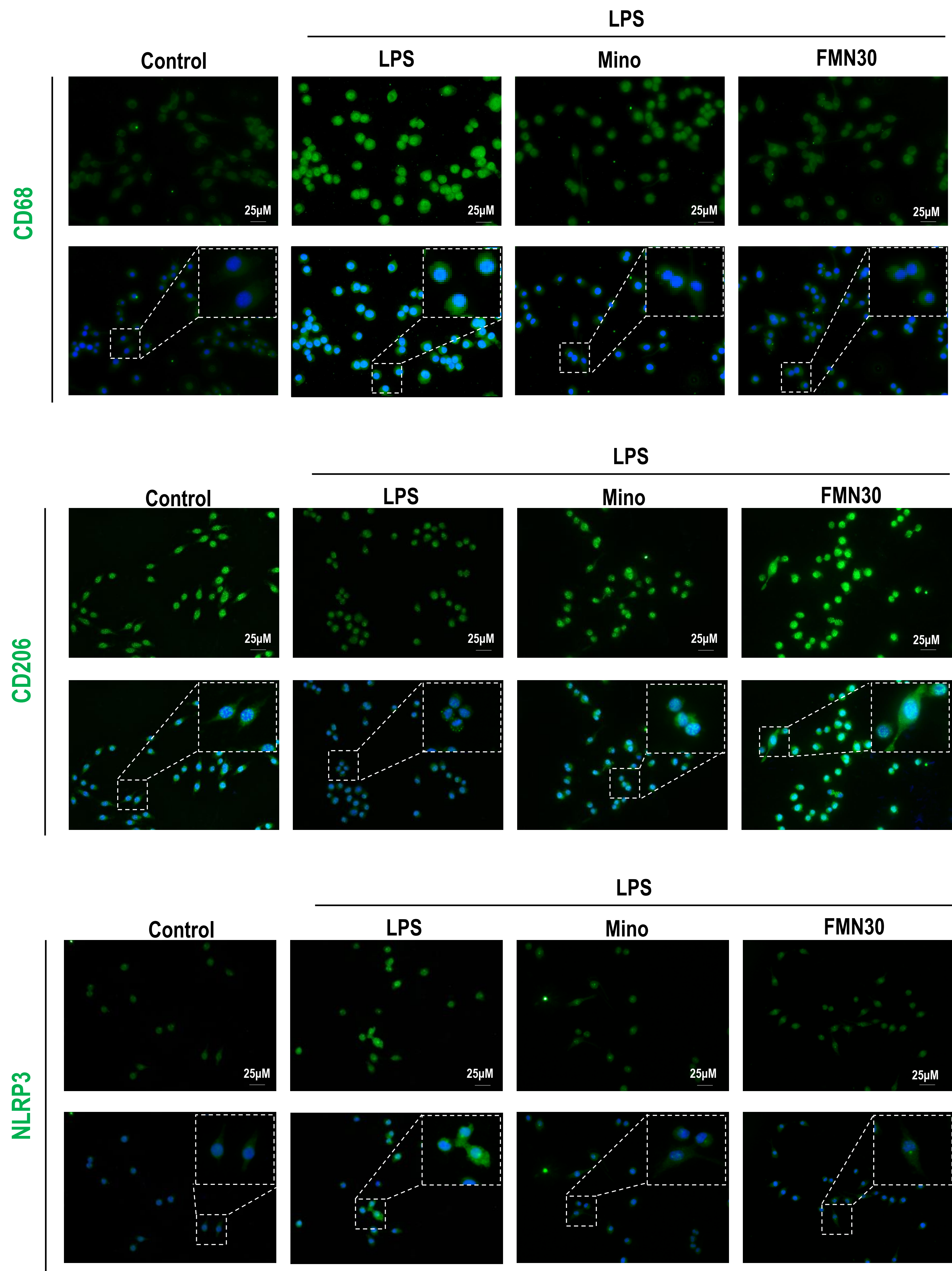

Fig 3 G

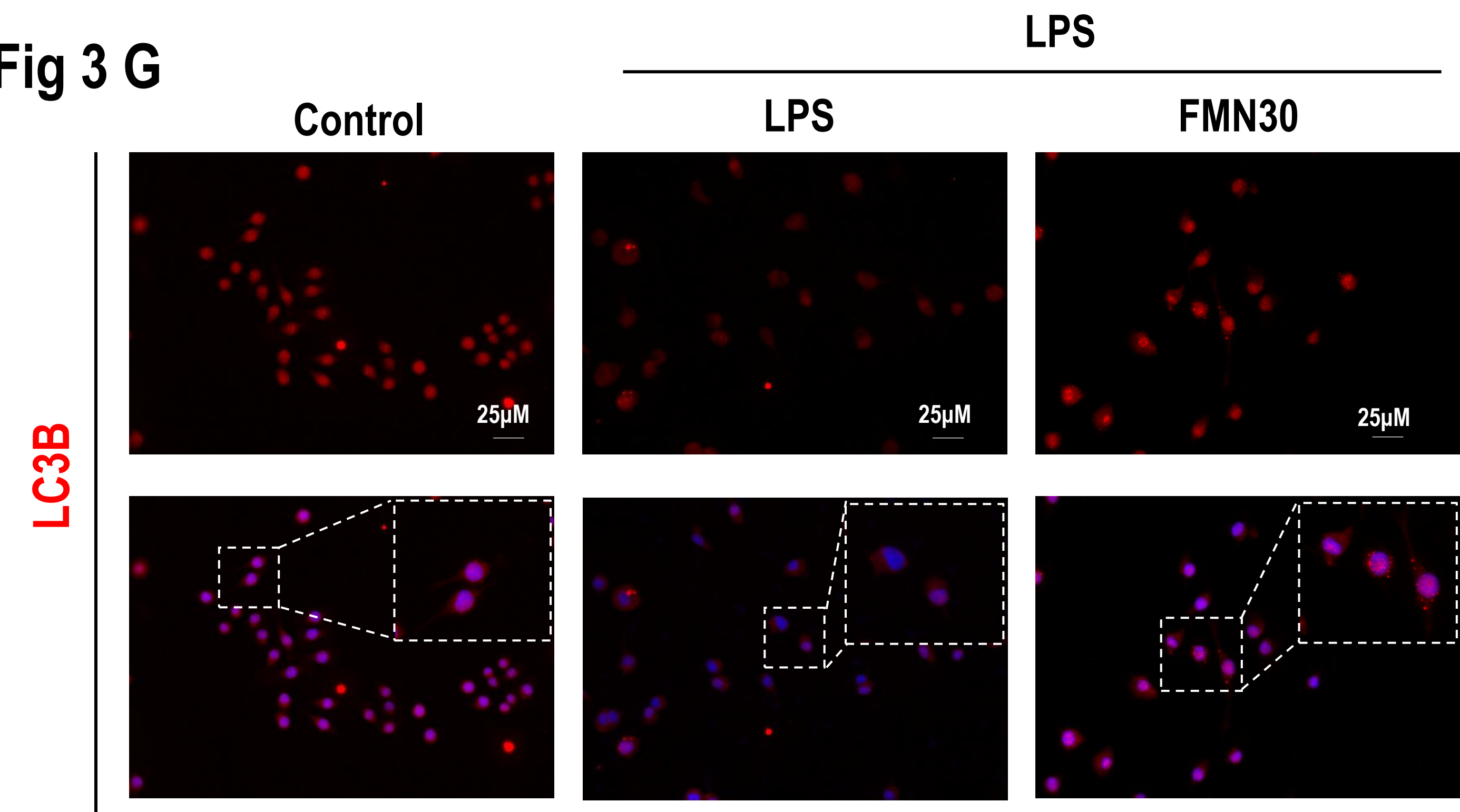

Fig 4 E

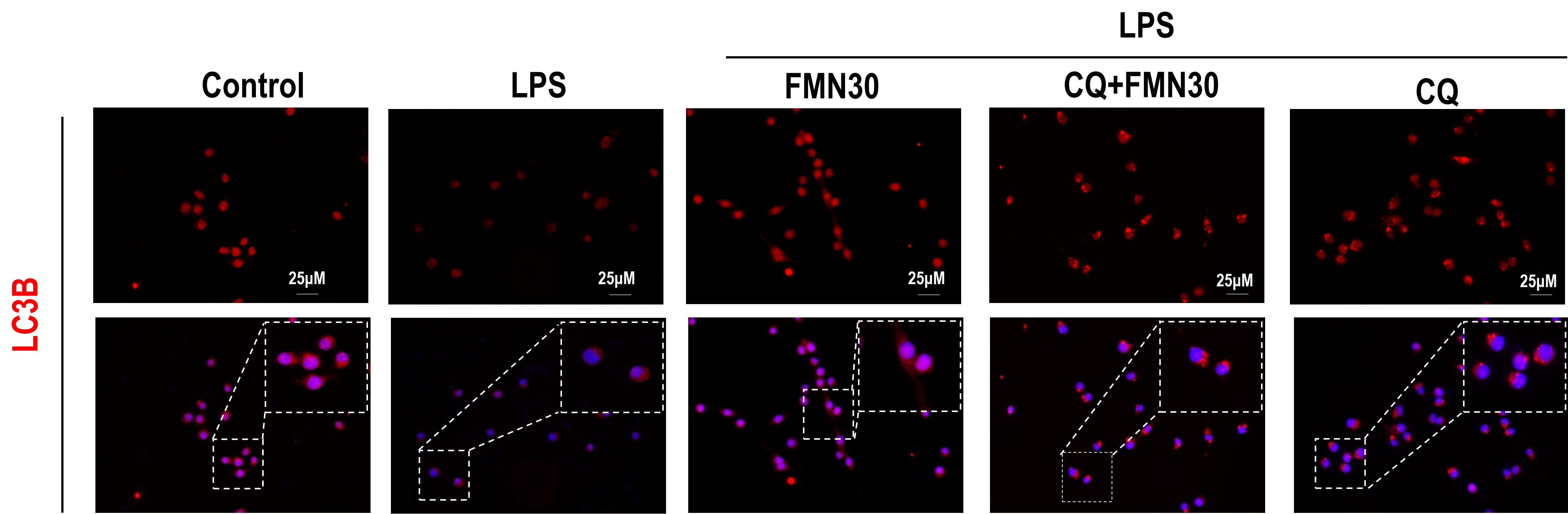

Fig 5 C

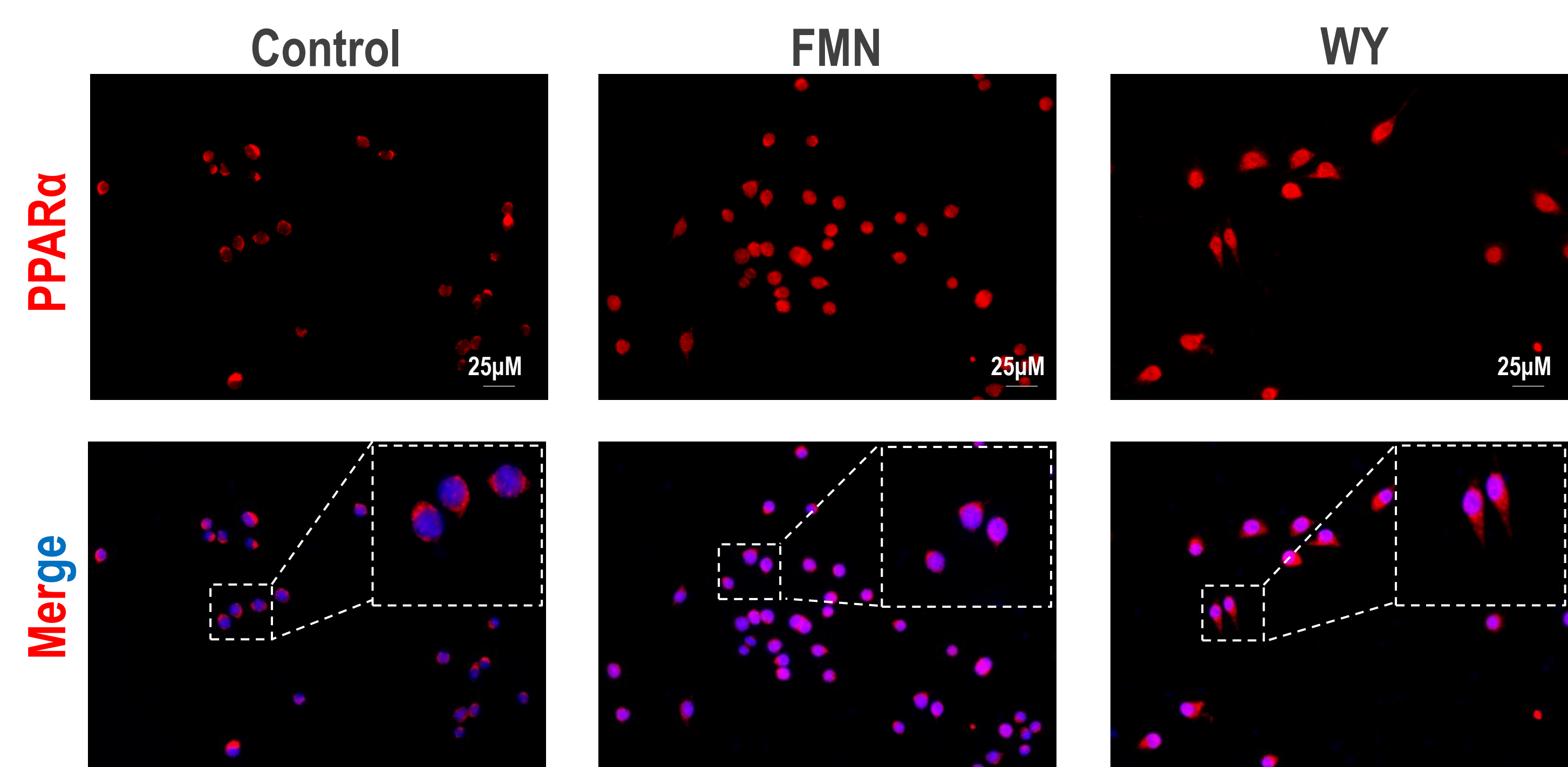

Fig 6 E

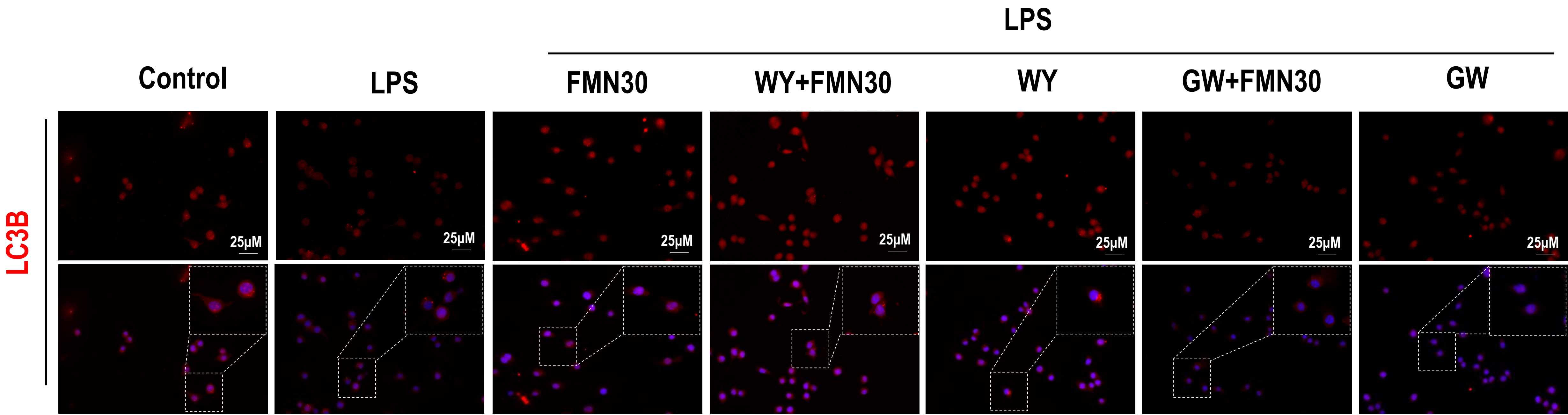

Fig 8 A

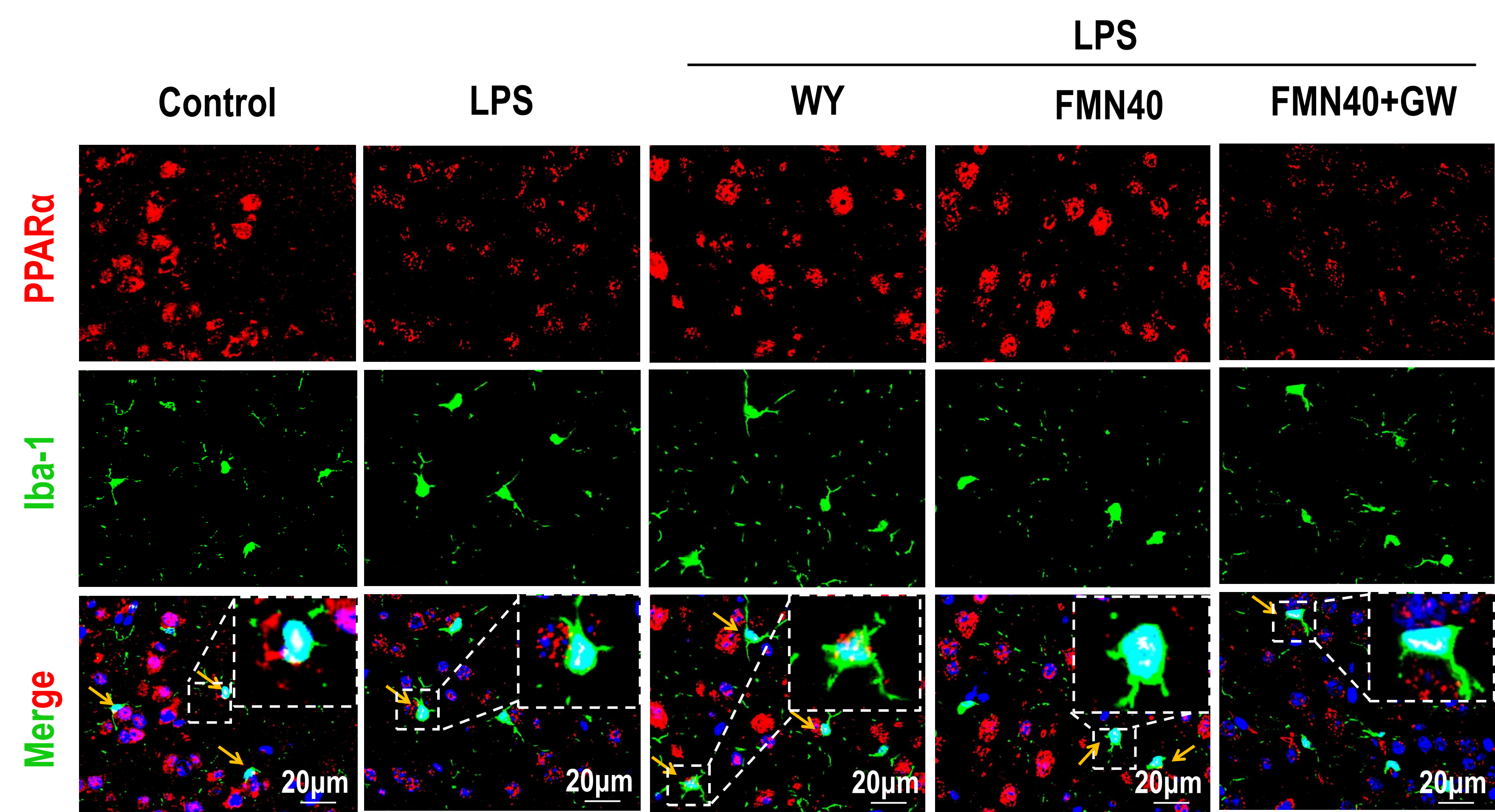

Fig 8 C

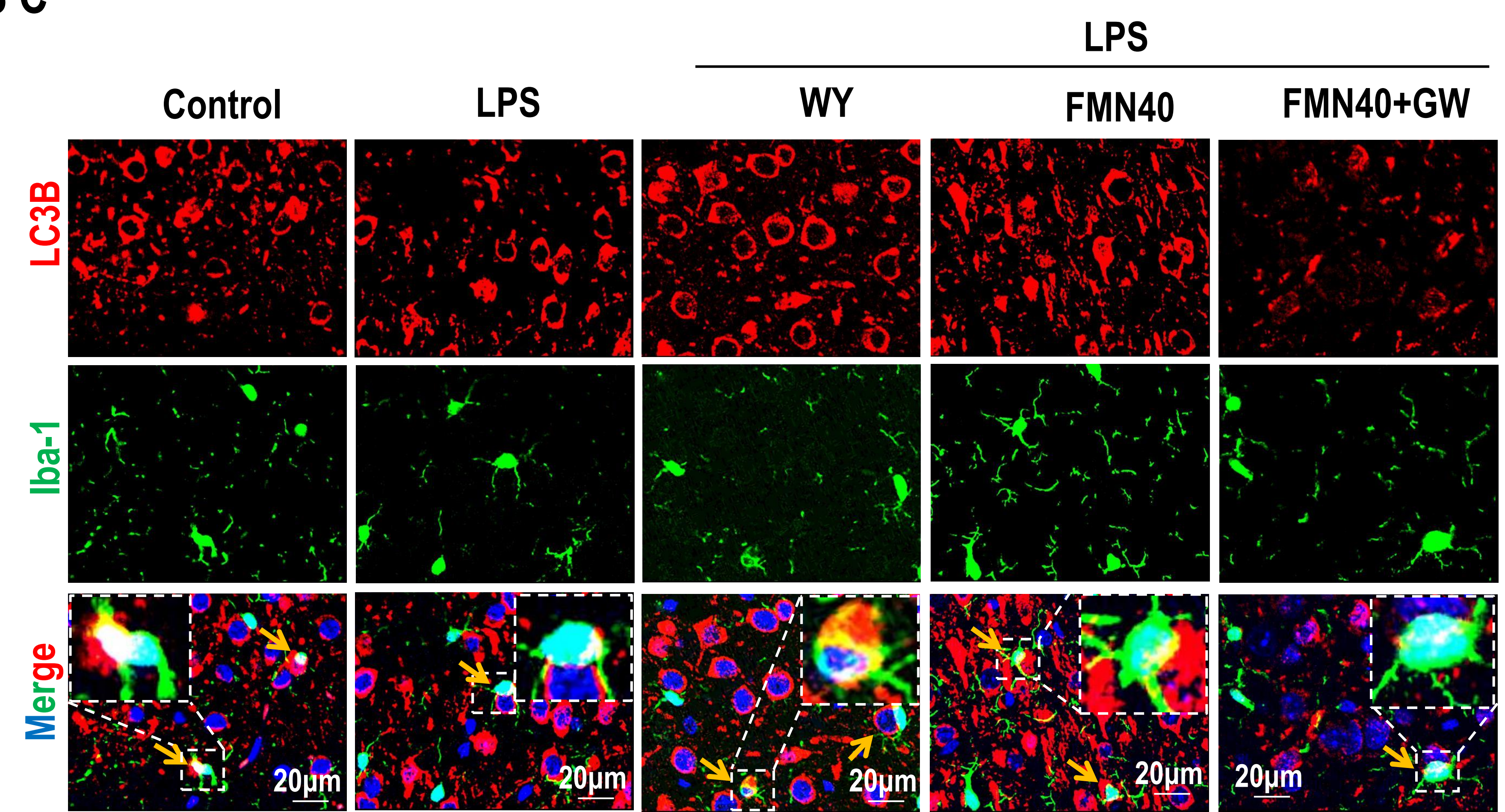

Fig 8 G

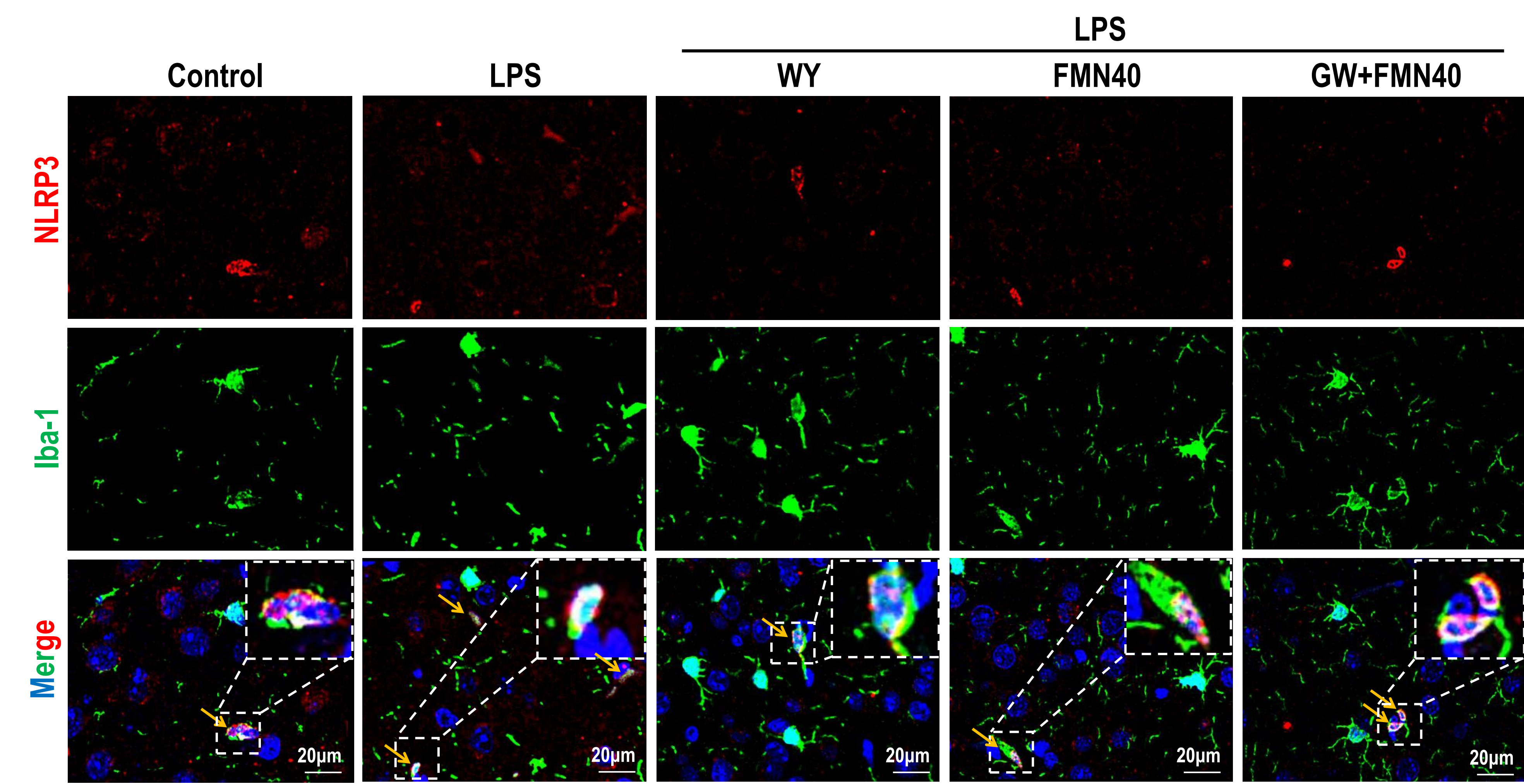

Fig 8 H

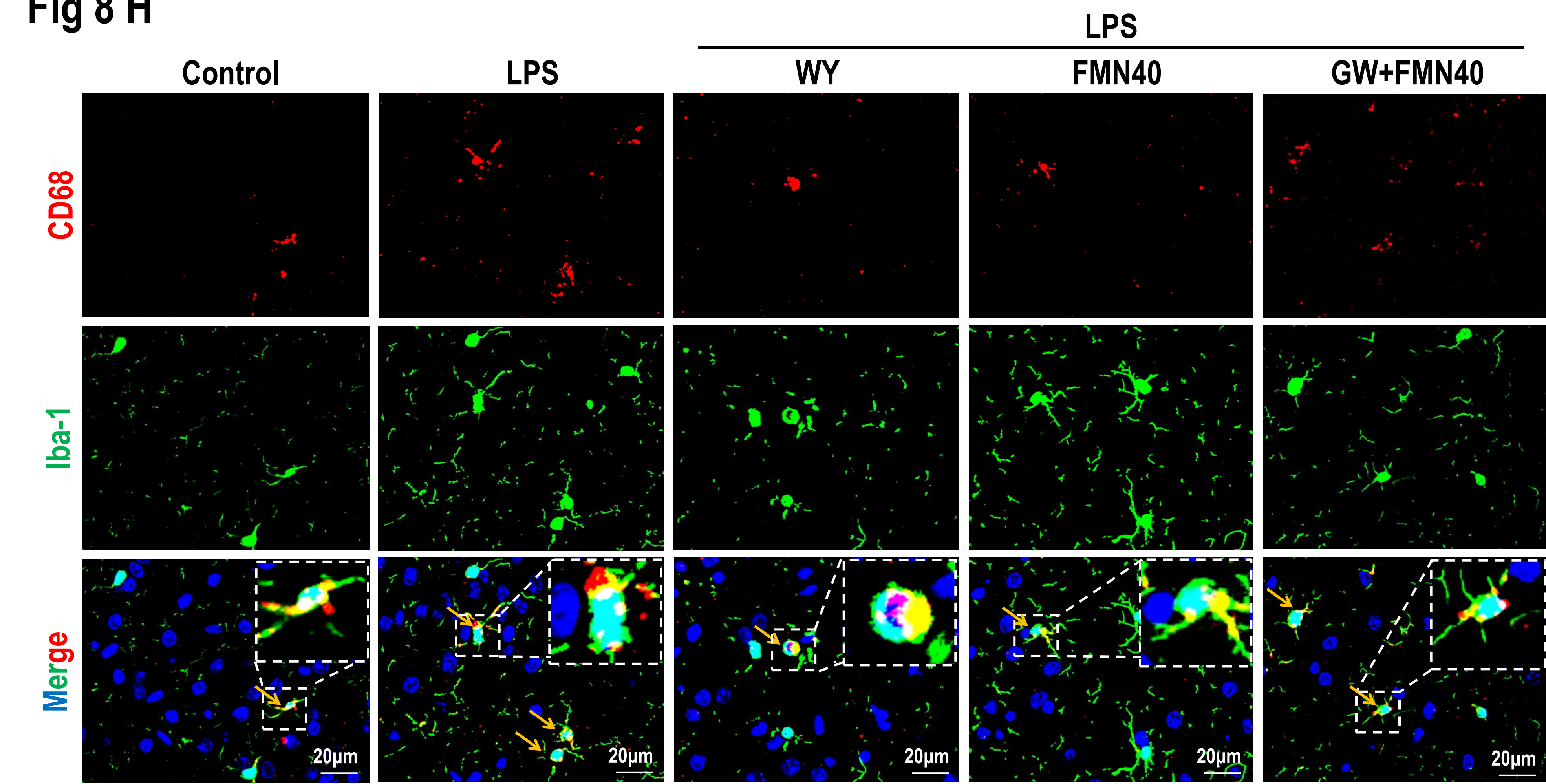

Fig 8 I

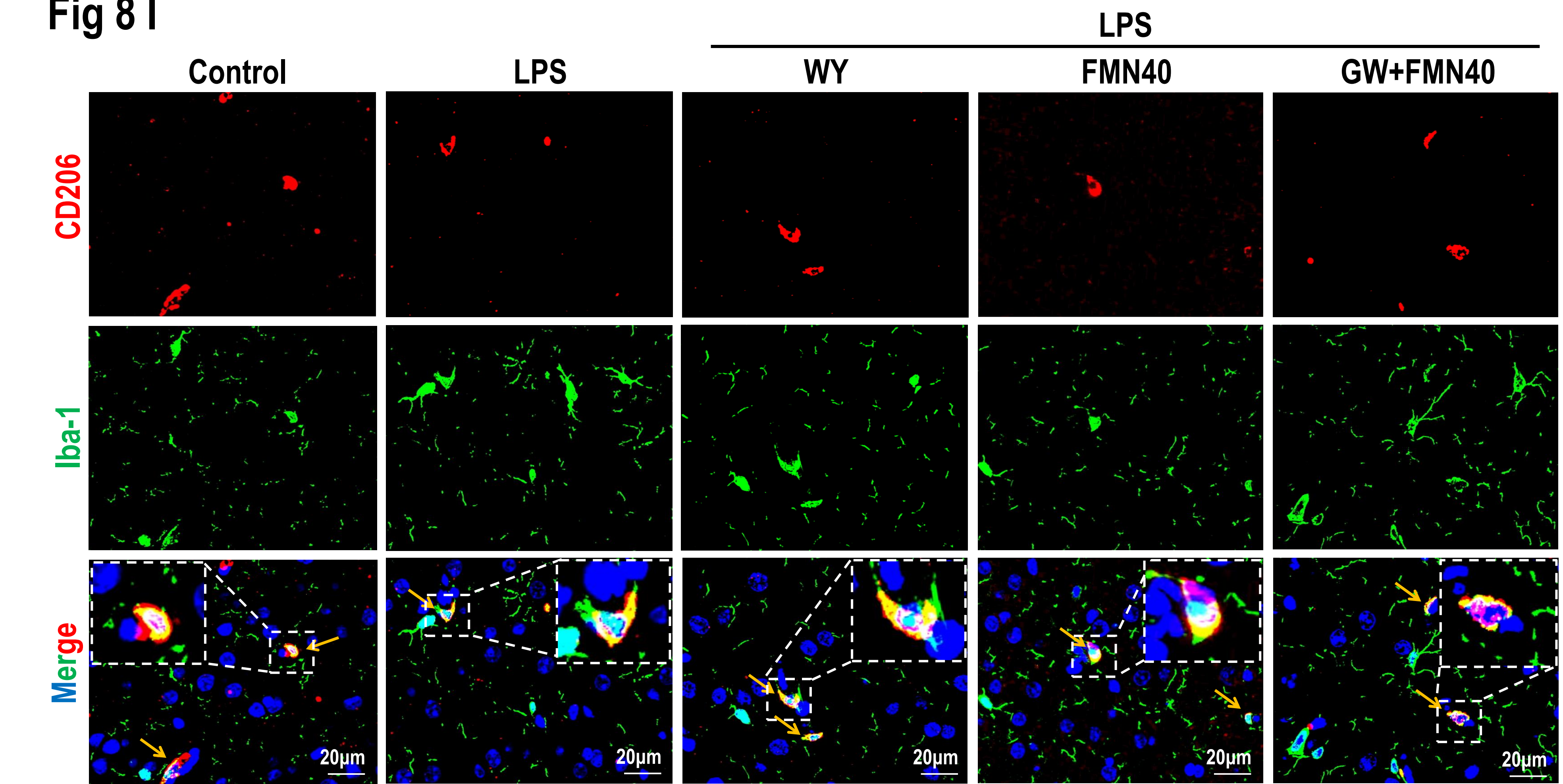

Fig. S2

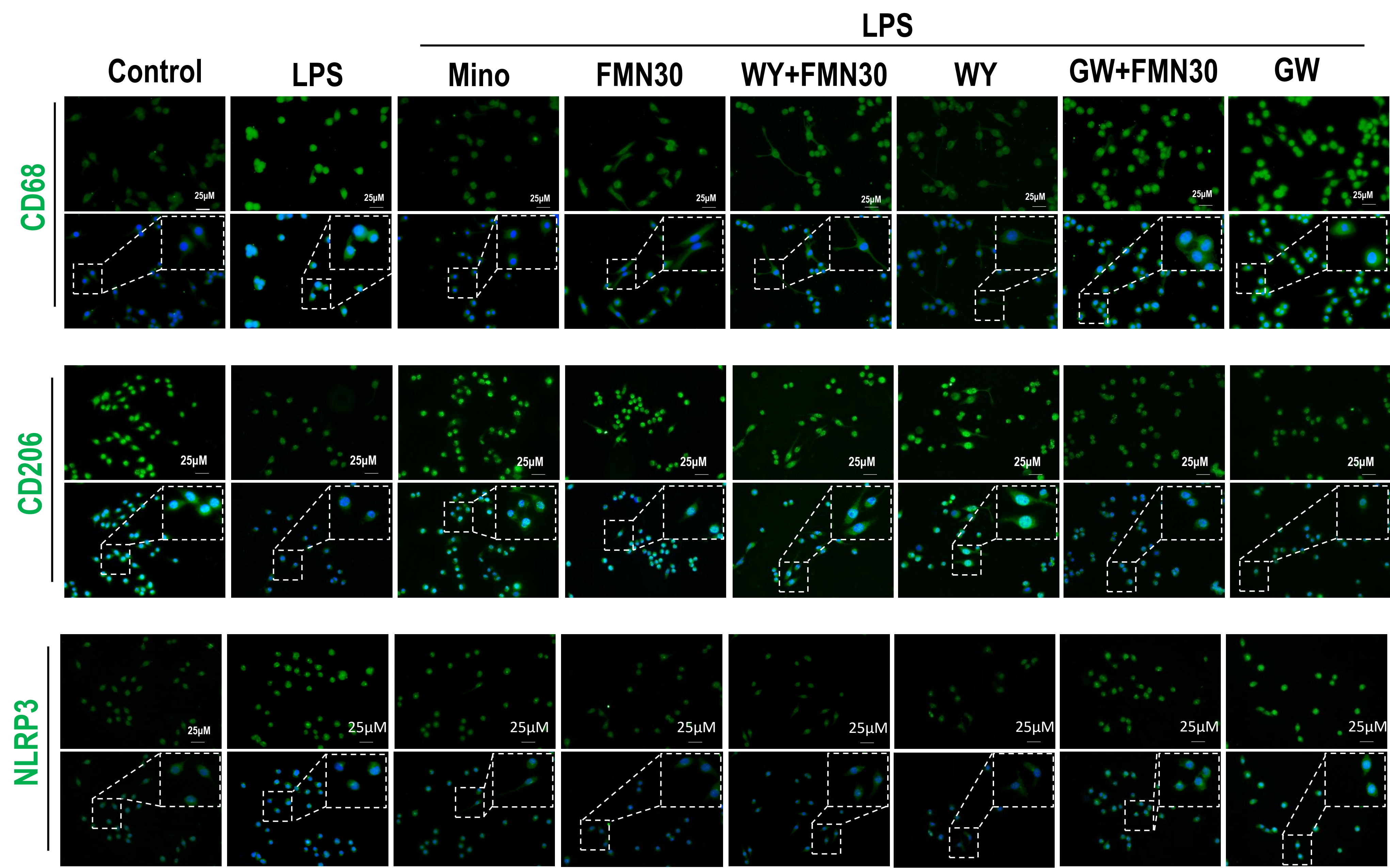

Fig. S3

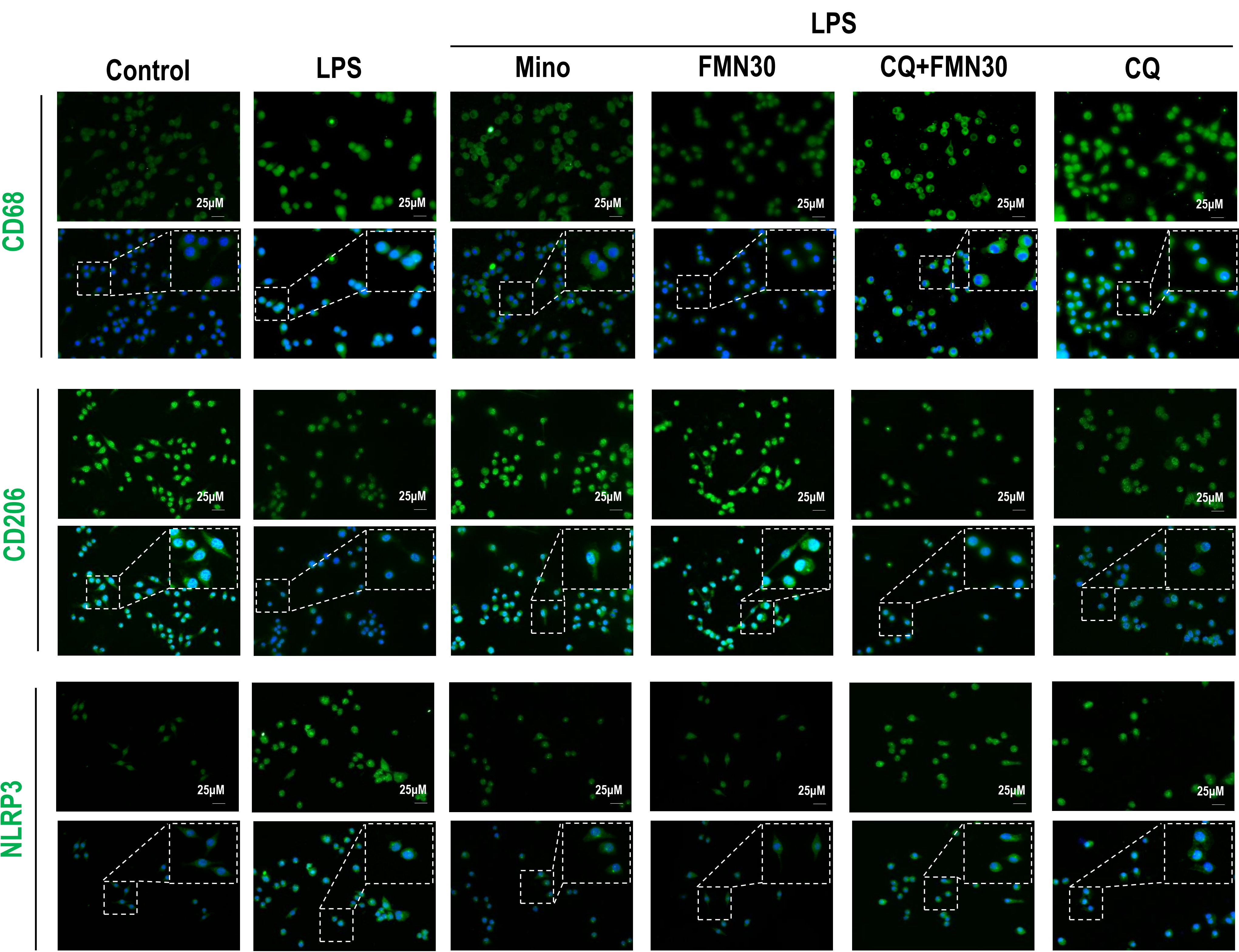

Supplement: Supplementary file 3 — Supplementary Material 3 [file 10020_2025_1217_MOESM3_ESM.pdf]
